# Activation of NF-κB and p300/CBP potentiates cancer chemoimmunotherapy through induction of MHC-I antigen presentation

**DOI:** 10.1073/pnas.2025840118

**Published:** 2021-02-18

**Authors:** Yixuan Zhou, Ingmar Niels Bastian, Mark D. Long, Michelle Dow, Weihua Li, Tao Liu, Rachael Katie Ngu, Laura Antonucci, Jian Yu Huang, Qui T. Phung, Xi-he Zhao, Sourav Banerjee, Xue-Jia Lin, Hongxia Wang, Brian Dang, Sylvia Choi, Daniel Karin, Hua Su, Mark H. Ellisman, Christina Jamieson, Marcus Bosenberg, Zhang Cheng, Johannes Haybaeck, Lukas Kenner, Kathleen M. Fisch, Richard Bourgon, Genevive Hernandez, Jennie R. Lill, Song Liu, Hannah Carter, Ira Mellman, Michael Karin, Shabnam Shalapour

**Affiliations:** ^a^Department of Pharmacology, School of Medicine, University of California San Diego, CA 92093;; ^b^Department of Biostatistics and Bioinformatics, Roswell Park Comprehensive Cancer Center, Buffalo, NY 14263;; ^c^Division of Medical Genetics, Health Sciences, Department of Biomedical Informatics, University of California San Diego, La Jolla, CA 92093;; ^d^Department of Medicine, University of California San Diego, La Jolla, CA 92093;; ^e^Laboratory of Gene Regulation and Signal Transduction, Department of Pharmacology, School of Medicine, University of California San Diego, La Jolla, CA 92093;; ^f^Department of Microchemistry, Proteomics, and Lipidomics, Genentech, Inc., South San Francisco, CA 94080;; ^g^Oncology Department, China Medical University Shengjing Hospital, 110004 Shenyang City, China;; ^h^Department of Cellular Medicine, Jacqui Wood Cancer Centre, University of Dundee, Dundee DD1 9SY, United Kingdom;; ^i^Biomedical Translational Research Institute and the First Affiliated Hospital, Jinan University, 510632 Guangzhou, Guangdong, China;; ^j^State Key Laboratory of Proteomics, Institute of Basic Medical Sciences, National Center of Biomedical Analysis, 100850 Beijing, China;; ^k^National Center for Microscopy and Imaging Research, Center for Research in Biological Systems, University of California San Diego, La Jolla, CA 92093;; ^l^Department of Urology, Moores Cancer Center, University of California San Diego, La Jolla, CA 92093;; ^m^Department of Immunobiology, Yale School of Medicine, New Haven, CT 06510;; ^n^Department of Dermatology, Yale School of Medicine, New Haven, CT 06510;; ^o^Center for Epigenomics, Department of Cellular and Molecular Medicine, School of Medicine, University of California San Diego, La Jolla, CA 92093;; ^p^Institute of Pathology, Medical University of Graz, A-8036 Graz, Austria;; ^q^Department of Pathology, Neuropathology and Molecular Pathology, Medical University of Innsbruck, A-6020 Innsbruck, Austria;; ^r^Department of Pathology, Christian Doppler Laboratory, Medical University of Vienna, 1090 Vienna, Austria;; ^s^Unit of Pathology of Laboratory Animals, University of Veterinary Medicine Vienna, 1210 Vienna, Austria;; ^t^Center for Computational Biology and Bioinformatics, Department of Medicine, University of California San Diego, La Jolla, CA 92093;; ^u^Department of Cancer Immunology, Genentech, Inc., South San Francisco, CA 94080;; ^v^Department of Cancer Biology, University of Texas MD Anderson Cancer Center, Houston, TX 77054

**Keywords:** histone acetylation, NF-κB, MHC-I, antigen presentation, immune checkpoint inhibitors

## Abstract

Many cancers evade immune rejection by suppressing major histocompatibility class I (MHC-I) antigen processing and presentation (AgPP). Such cancers do not respond to immune checkpoint inhibitor therapies (ICIT) such as PD-1/PD-L1 [PD-(L)1] blockade. Certain chemotherapeutic drugs augment tumor control by PD-(L)1 inhibitors through potentiation of T-cell priming but whether and how chemotherapy enhances MHC-I–dependent cancer cell recognition by cytotoxic T cells (CTLs) is not entirely clear. We now show that the lysine acetyl transferases p300/CREB binding protein (CBP) control MHC-I AgPPM expression and neoantigen amounts in human cancers. Moreover, we found that two distinct DNA damaging drugs, the platinoid oxaliplatin and the topoisomerase inhibitor mitoxantrone, strongly up-regulate MHC-I AgPP in a manner dependent on activation of nuclear factor kappa B (NF-κB), p300/CBP, and other transcription factors, but independently of autocrine IFNγ signaling. Accordingly, NF-κB and p300 ablations prevent chemotherapy-induced MHC-I AgPP and abrogate rejection of low MHC-I–expressing tumors by reinvigorated CD8^+^ CTLs. Drugs like oxaliplatin and mitoxantrone may be used to overcome resistance to PD-(L)1 inhibitors in tumors that had “epigenetically down-regulated,” but had not permanently lost MHC-I AgPP activity.

## Significance

T cells recognize their targets via their T-cell receptors (TCRs), which in the case of CD8^+^ T cells bind to MHC-I:antigen complexes on the surface of target cells. Many cancer cells evade immune recognition and killing by down-regulating MHC-I AgPPM. Here, we show how the histone acetyl transferases p300/CBP together with NF-κB epigenetically regulate expression of MHC-I molecules, immunoproteasome subunits, and peptide transporter to enable proper MHC-I antigen presentation. Notably, this pathway is frequently disrupted in human cancers. We now show that certain chemotherapeutics can augment MHC-I antigen presentation via NF-κB and p300/CBP activation, thereby enhancing cancer cell recognition and killing by effector CD8^+^ CTLs.

Immune checkpoint inhibitor therapy (ICIT) had transformed cancer treatment (1–4), but even in ICIT-responsive metastatic melanoma and nonsmall cell lung cancer (NSCLC), response rates rarely exceed 40% (5). Other malignances, including prostate cancer (PCa) and pancreatic ductal adenocarcinoma (PDAC), are ICIT refractory (6–9). For a given neoplasm to respond to immune checkpoint inhibition, in particular PD-(L)1 blockade, it needs to be populated by cytotoxic T cells (CTLs) that recognize tumor antigens (4). However, even CTL-populated tumors can evade immune elimination either through activation of immunosuppressive mechanisms that induce CD8^+^ T-cell suppression or restrain their entry into tumors (10), down-regulation of major histocompatibility class I (MHC-I) AgPP (11, 12), or antigen editing and loss (13). Various strategies have been used to enhance ICIT responsiveness, including induction of immunogenic cells death (ICD) by radiotherapy and chemotherapy (14). By enhancing the release of damage associated molecular patterns (DAMPs) and other molecules, ICD stimulates tumor antigen uptake by antigen-presenting cells (APCs) that prime T cells against tumor antigens, as demonstrated by vaccination experiments (15). Primed T cells may accumulate in the tumor and lead to immune rejection as long as they can recognize and kill their targets (16). Such strategies are ineffective in cancers with low MHC-I or HLA-A/B/C expression (11, 16–19).

PCa is a typical ICIT-refractory cancer, presumably due to low expression of HLA-A/B/C molecules that together with β2 microglobulin form MHC-I heterodimers, which present tumor antigens to CD8^+^ CTL (20, 21). Using mouse models of PCa we found that the platin-based DNA-crosslinker oxaliplatin (Oxali) potentiates immune rejection of autochthonous or engrafted tumors after genetic or pharmacological depletion of PD-L1–expressing immunosuppressive IgA^+^ plasmocytes, which cause CTL exhaustion (22). Low-dose Oxali also enhances mouse PCa regression in response to anti–PD-L1 treatment (22). Similar results were obtained with low-dose Oxali or photodynamic therapy in other cancer models (23, 24) but the underlying mechanisms have not been explored. As Oxali is known to induce ICD and T-cell priming, we investigated whether its ability to potentiate the immune rejection of IgA^+^ plasmocyte-depleted or anti–PD-L1–treated low MHC-I prostate tumors also entails effects on the recognition and killing step of the cancer-immunity cycle, which depends on CTL–MHC-I interactions (16, 25). Here we show that Oxali and the structurally unrelated topoisomerase II inhibitor mitoxantrone (Mito) transcriptionally up-regulate expression of MHC-I molecules and their cognate antigen presentation and processing machinery (AgPPM). This response, which takes place in human and mouse cancers, depends on activation of nuclear factor kappa B (NF-κB) and nuclear translocation of the closely related histone (and lysine) acetyltransferases p300 and CREB binding protein (CBP). Whereas p300 ablation abrogated MHC-I AgPP induction and the synergy between low-dose Oxali and PD-(L)1 blockade, it had no effect on induction of antitumor immunity by Oxali-killed PCa cells used as an immunogen.

## Results

### Oxaliplatin and Mitoxantrone Induce MHC-I AgPPM Genes.

**Fig. 1.**
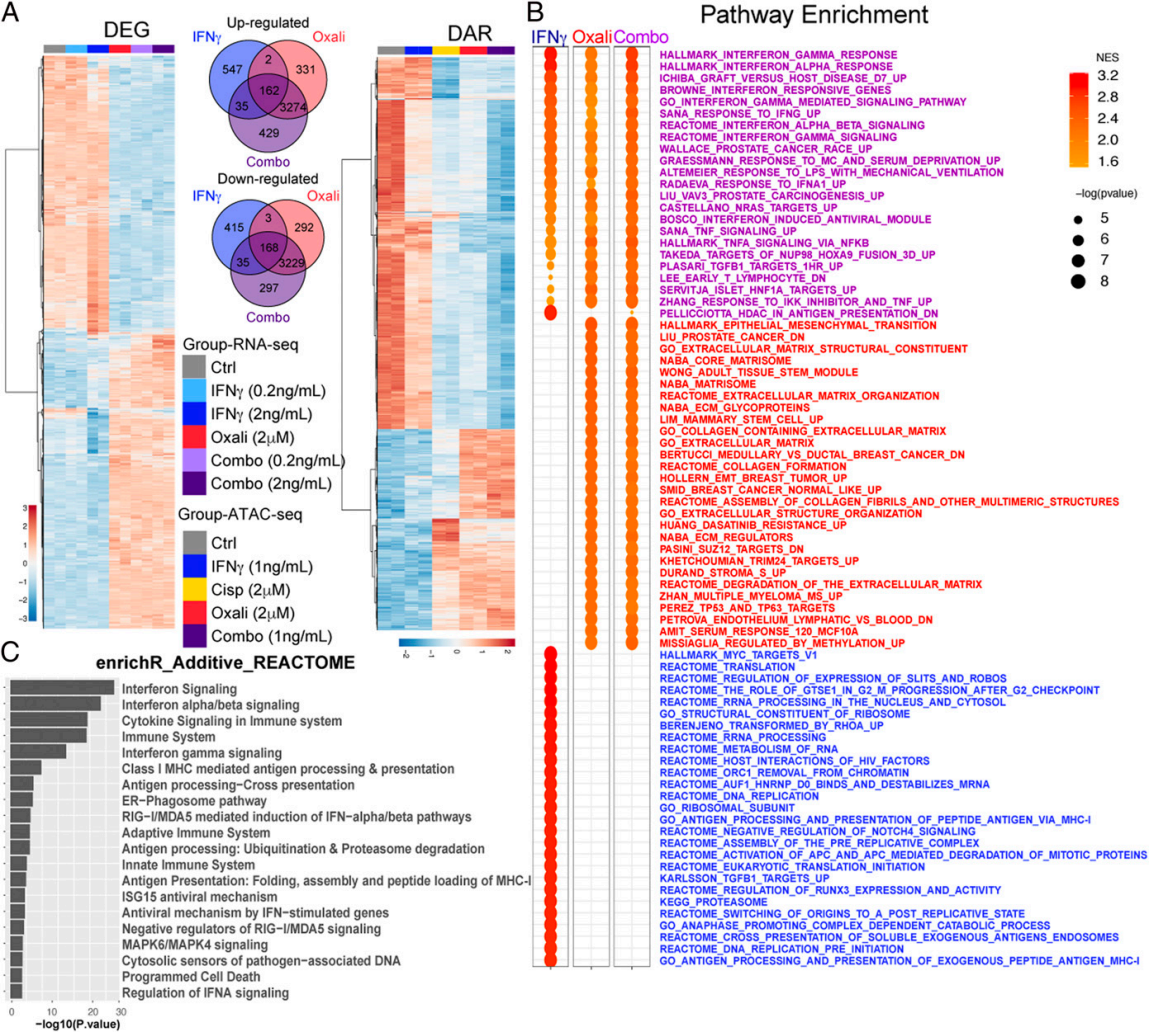
Chemotherapy induces MHC-I AgPPM genes. (*A*) Heatmap showing all DEGs identified in bulk RNA-seq of Myc-CaP cells treated with IFNγ (0.2 or 2 ng/mL), Oxali (2 μM), or both (combo) (*Left*). Venn diagram shows overlapping DEGs between IFNγ (2 ng/mL), Oxali, and combo treatment groups relative to Ctrl (*Middle*) and heatmap shows all DARs identified in bulk ATAC-seq of Myc-CaP cells treated as indicated relative to control (*Right*). (*B*) Gene set enrichment analysis (GSEA) was applied to expression profiles specific to each treatment group relative to Ctrl. Top 30 significantly enriched pathways for each respective comparison are shown. Some pathways were considered both IFNγ and Oxali driven (purple), while others were specific to either IFNγ (blue) or Oxali (red). (*C*) Functional enrichment was applied to genes classified with additive response to combination therapy. The top 20 enriched REACTOME pathways are shown.

To determine the effect of Oxali and related drugs on gene expression in PCa cell lines used in our previous study (22), Myc-CaP cells were treated with different drugs at doses that induce no more than 10% cell death, and vital cells (*SI Appendix*, Fig. S1 *A* and *B*) were analyzed by whole genome RNA sequencing (RNA-seq) and assay for transposase-accessible chromatin (ATAC-seq) (26). Since CTL reinvigoration by anti–PD-L1 induces IFNγ production (27, 28), we also examined the effect of IFNγ alone or together with chemotherapy. Low-dose chemotherapy, in particular Oxali, induced marked changes in gene expression and chromatin accessibility depicted as differentially expressed genes (DEGs) and differentially accessible DNA regions (DARs) (Fig. 1*A* and *SI Appendix*, Fig. S1 *C*–*F*). The platinoid-induced changes were usually augmented by IFNγ, although the effects of Oxali were broader than that of IFNγ, which mainly enhanced gene expression magnitude rather than breadth. Some of the Oxali or IFNγ-inducible gene sets were common to both agents (Fig. 1*A*). Pathway enrichment analysis (Fig. 1*B*) identified the most significantly enriched pathways, activated by Oxali (red, e.g., epithelial-mesenchymal transition, TP53), IFNγ (blue, e.g., Myc), or Oxali + IFNγ together (purple, e.g., IFN type I and II, AgPPM). Notably, while either Oxali or IFNγ significantly enriched genes involved in MHC-I AgPP and IFNγ signaling, these effects were strongly enhanced when Oxali and IFNγ were combined (Fig. 1*B* and *SI Appendix*, Fig. S2 *A*–*C*). However, Oxali did not induce IFNγ expression, indicating that its ability to induce MHC-I AgPPM components was not due to autocrine IFNγ signaling.

**Fig. 2.**
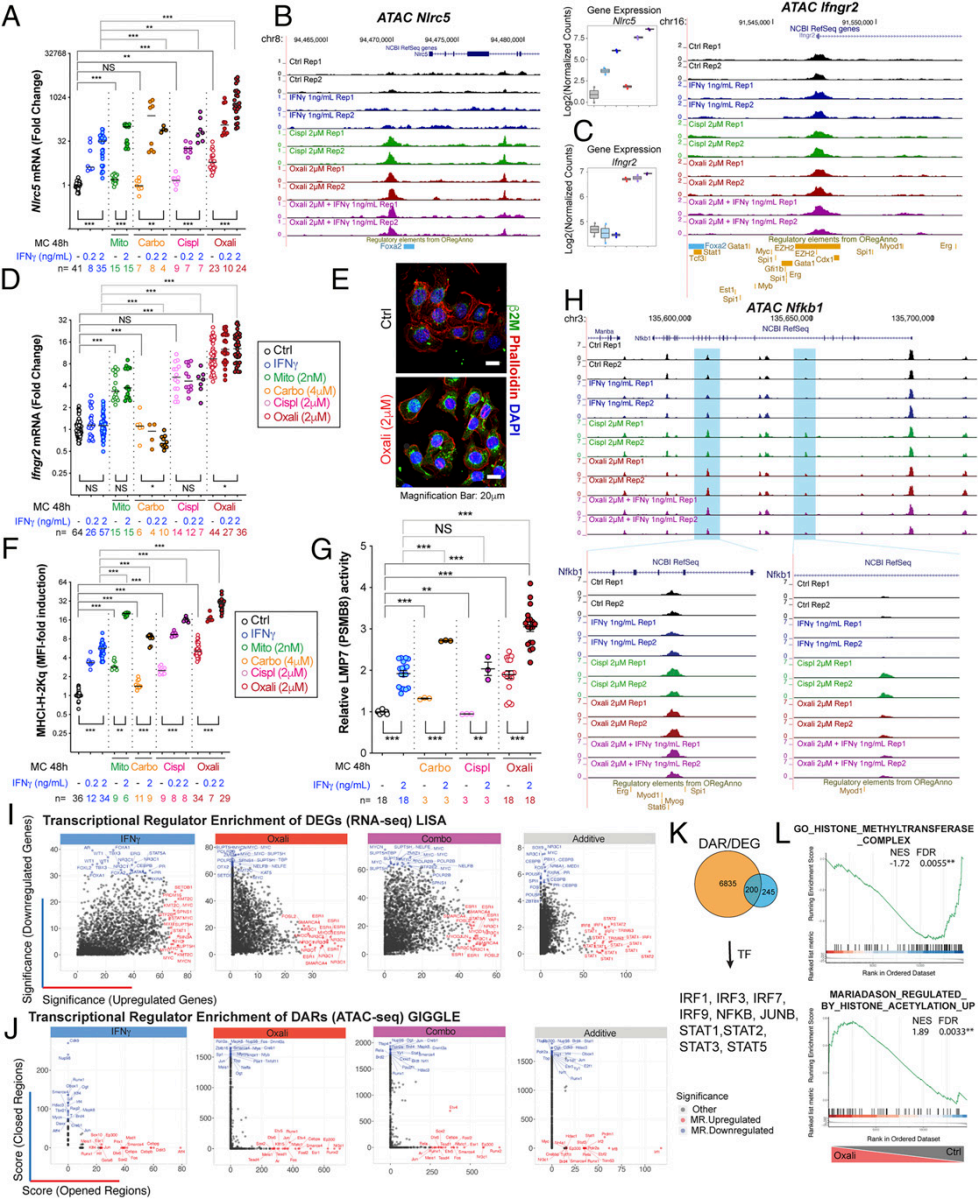
Transcriptional regulators of Oxali-induced MHC-I AgPPM genes. (*A*) RNAs from Myc-CaP cells incubated as indicated with IFNγ, Mito, Oxali, carboplatin (Carbo), or Cispl for 48 h were analyzed by qRT-PCR using *Nlrc5* primers. (*B* and *C*) Candidate genomic loci for *Nlrc5* (*B*) and *Ifngr2* (*C*), showing library-size normalized read pair pileup profiles determined by ATAC-seq across samples. Expression of respective genes determined by RNA-seq is also shown. (*D*) RNAs from Myc-CaP cells incubated as indicated were analyzed by qRT-PCR using *Ifngr2* primers. (*E*) Myc-CaP cells incubated with Oxali for 12 h were stained with β2M antibody (green) and phalloidin (red) and counterstained with DAPI. (Scale bar: 20 μm.) (*F*) Myc-CaP cells treated as indicated were analyzed for surface MHC-I (H-2Kq) expression by flow cytometry. (*G*) Myc-CaP cells were incubated as indicated and lysed. LMP7 (PSMB8) immunoproteasome activity was measured using a fluorogenic LMP7-specific substrate peptide Ac-ANW-AMC. (*H*) Candidate genomic locus for *Nfkb1* showing read density profiles determined by ATAC-seq across samples. (*I*) LISA was applied to DEGs identified in comparisons of IFNγ, Oxali, and both (combo)-treated cells relative to control, as well as to genes classified with additive response (top 500 up-regulated, down-regulated DEGs). The top 20 enriched regulators of up-regulated (red) and down-regulated (blue) DEGs are noted. (*J*) GIGGLE applied to DARs identified in comparisons of IFNγ-, Oxali-, and combo-treated cells relative to control, as well as to regions classified with additive response. The top 20 enriched regulators of opened (red) and closed (blue) DARs are noted. (*K*) Two hundred common genes were identified by comparing TFs found by DEG and DAR analysis. (*L*) Gene set enrichment analysis (GSEA) was applied to expression profiles determined in Oxali-treated cells relative to control. The literature-curated known regulatory elements from ORegAnno database are shown. Candidate enrichment plots for representative pathways related to histone methylation (*Top*) and acetylation (*Bottom*) are shown. Two-sided *t* test (means ± SEM), and Mann–Whitney test (median) were used to determine significance between two groups. One-way ANOVA analysis (*A*, *D*, *F*, *G*) and multiple comparison confirmed the results. *P < 0.05; **P < 0.01; ***P < 0.001; NS, not significant. Specific *n* values are shown in *A*, *D*, *F*, and *G*, each experiment includes at least three biological replicates.

Pathway enrichment analysis of DEGs that were responsive to Oxali plus IFNγ revealed strong induction of genes related to type I and II IFN signaling and MHC-I AgPPM components, involved in protein folding, MHC-I complex assembly, and peptide loading, as well as genes involved in the endoplasmic reticulum (ER)-phagosome pathway and antiviral responses (Fig. 1*C* and *SI Appendix*, Fig. S2*D*). Most of these genes were also induced by Oxali alone. To understand how these genes were induced we examined the ATAC-seq patterns of a gene cluster on mouse chromosome 17 harboring the *Psmb9*, *Tap1*, *Psmb8*, and *Tap2* genes, coding for immunoproteasome components and peptide transporters (*SI Appendix*, Figs. S1 *E* and *F* and S3*A*). Low-dose Oxali, and to a lesser extent cisplatin (Cispl), increased transcription factor (TF) accessibility at several sites within this locus (*SI Appendix*, Fig. S3*A*). Surprisingly, IFNγ alone had little effect, if any, on chromatin structure (*SI Appendix*, Fig. S1*F*). qRT-PCR analysis further confirmed induction of AgPPM genes by Oxali and Cispl and to a lesser extent by Mito, alone or together with IFNγ (*SI Appendix*, Fig. S3 *B*–*D*). A similar response pattern was displayed by the *Nlrc5* gene coding for NLRC5/CITA, the master activator of the MHC-I AgPPM (Fig. 2*A*). ATAC-seq revealed increased *Nlrc5* chromatin accessibility after low-dose Oxali or Cispl, but hardly any change after IFNγ treatment (Fig. 2*B*). Mito, Cispl, and Oxali, but not IFNγ, induced *Ifngr2* mRNA, but had little effect on chromatin accessibility of its gene (Fig. 2 *C* and *D*). Of note, the chromosome 17 region opened up by Oxali contains binding motifs recognized by BORIS and CTCF (*SI Appendix*, Fig. S3*A*), general TF responsible for chromatin opening (29). Low-dose Oxali also increased β2 microglobulin (β2M), and all tested chemotherapeutics induced surface and mRNA expression of H-2Kq, the predominant MHC-I molecule in Myc-CaP cells (Fig. 2 *E* and *F* and *SI Appendix*, Fig. S3*D*). Low-dose Oxali increased immunoproteasome activity measured with an LMP7/PSMB8-specific substrate, Ac-ANW-AMC, an effect that was potentiated by IFNγ (Fig. 2*G*).

### Putative Transcriptional Regulators of MHC-I AgPPM Induction.

We searched for signaling pathways and TF-mediating MHC-I AgPPM and IFNγR2 induction by low-dose Oxali. RNA-seq and pathway enrichment analyses suggested involvement of IRF, STAT, NF-κB, MYC family members, and androgen receptor (AR) (Fig. 2 *H*–*K* and *SI Appendix*, Fig. S3 *E* and *F*). Whereas the IRF, STAT, and NF-κB pathways were up-regulated by Oxali and potentiated by IFNγ, the MYC and to a lesser extent the AR pathway, both of which participate in PCa tumorigenesis (30–32), were down-regulated after Oxali + IFNγ treatment. Among IRF family members, IRF1, 7, and 9 were stimulated by Oxali and IFNγ, IRF2 was induced by Oxali, and IRF8 mainly responded to IFNγ (*SI Appendix*, Fig. S3*F*). Similarly, STAT1 and 2 were stimulated by Oxali, whereas IFNγ induced STAT1 and 3. JUN, ATF3, UBA7, CREB3, NFE2L1, and SOCS1 were induced by low-dose Oxali, along with NF-κB1 (p105) and NF-κB2 (p100) (*SI Appendix*, Fig. S3*F*). ATAC-seq confirmed that Oxali, but not IFNγ, enhanced chromatin accessibility of the *Nfkb1* locus (Fig. 2*H*).

We employed two additional analytic approaches to identify master TF-mediating treatment-induced expression changes [LISA (33)] and TF binding enrichment within regions of differential chromatin accessibility [GIGGLE (34)]. These analyses predicted the master regulators (MRs) most likely to influence the DEGs (Fig. 2*I*) and DARs (Fig. 2*J*) by leveraging the complete set of TF binding datasets available from the CistromeDB collection. The results further highlighted treatment-related directional TF associations (up/down-regulated DEGs, open/closed DARs); Oxali: ESR1, NR3C1, and JUN; IFNγ: MYC, STAT1, ATF4, and FOS; Oxali + IFNγ: STAT1, YAP1, ESR1, IRF1, RELA, and IRF8 (Fig. 2 *I* and *J*). DEG and DAR integration revealed 200 common TFs, including IRFs and STATs (Fig. 2*K*). Notably, Oxali treatment elicited marked changes in histone methylation- and acetylation-related gene signatures (Fig. 2*L*) in agreement with the ATAC-seq data (*SI Appendix*, Fig. S1 *E* and *F*). Subset analyses focusing on histone modifying factors implicated the involvement of the histone acetyltransferases (HATs) p300 and CBP and several histone deacetylases (HDACs) (*SI Appendix*, Fig. S4*A*).

To confirm induction and/or activation of some of the above TFs, PCa (Myc-CaP, TRAMP-C2/TRC2), and colon cancer (MC38) cell lines were treated as above with or without IFNγ. Protein immunoblotting (IB) and flow cytometric analyses confirmed induction of ER stress (P-eIF2α, CHOP) and DNA damage (p-p53, γH2AX, and p-ATM) markers, IRFs (IRF1, p-IRF3, and IRF7), STATs (P-STAT1 and STAT1), CREB1, JUNB, type I IFN-inducing proteins (cGAS and STING), PSMB9, and NF-κB signaling components (IκBα, RELA/p65, and P-p65) (*SI Appendix*, Fig. S4 *B*–*G*).

### Chemotherapy Stimulates HAT Nuclear Localization and Activity.

**Fig. 3.**
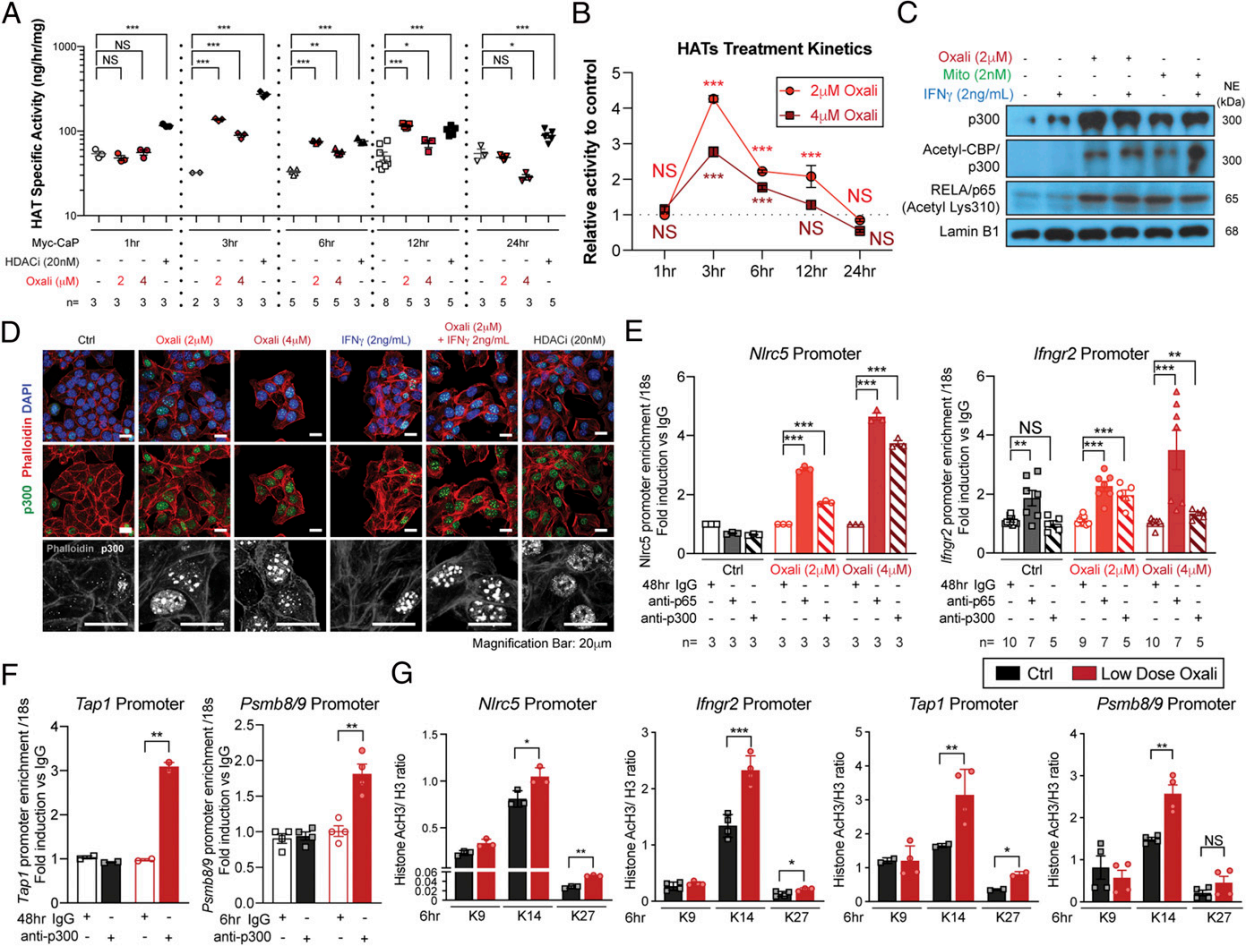
Oxaliplatin and mitoxantrone stimulate HAT activity and nuclear localization. (*A* and *B*) Myc-CaP cells incubated with Oxali (2 or 4 μM) or the HDACi panobinostat (LBH589; 20 nM) for the indicated times were lysed and analyzed for HAT activity using H3 as a substrate. (*C*) Nuclear extracts of Myc-CaP cells treated with Oxali, Mito, and/or IFNγ were IB analyzed for p300, acetylated-CBP/p300, acetylated-RELA/p65 (lysine K310), and lamin B1 (loading control). (*D*) Myc-CaP cells treated as indicated for 12 h were stained with anti-p300 (green) and phalloidin (red; actin cables). Nuclei were counterstained with DAPI (blue). (Scale bar: 20 μm.) (*E*–*G*) Untreated and Oxali-treated Myc-CaP cells were subjected to ChIP analysis with control IgG and antibodies to p65/RelA, p300 as indicated (*E* and *F*) or acetylated H3 (lysine K9, K14, and K27) (*G*). Precipitation of the indicated promoter regions was determined by PCR. Two-sided *t* test (means ± SEM), and Mann–Whitney test (median) were used to determine significance. *P < 0.05; **P < 0.01; ***P < 0.001; NS, not significant. Specific *n* values are shown in *A* and *E*; each experiment includes at least three biological replicates.

Chromatin structure opening, as revealed by ATAC-seq analysis, depends on histone acetylation (35). Importantly, Oxali treatment of Myc-CaP cells, increased HAT enzymatic activity within 3 h and its effect was comparable to that of a HDAC inhibitor (HDACi) (Fig. 3 *A* and *B*). Oxali and Mito also increased total p300, acetylated CBP/p300, and K310-acetylated RELA/p65 nuclear amounts (Fig. 3*C*). IFNγ also increased nuclear p300, but its effect was considerably weaker than that of Oxali (Fig. 3 *C* and *D*). Both Oxali and HDACi induced p300 nuclear translocation in murine PCa cells (Fig. 3*D*) and human PCa organoids (*SI Appendix*, Fig. S5*A*). Chromatin immunoprecipitation (ChIP) experiments showed that Oxali induced p300 and RELA/p65 recruitment to the *Nlrc5* and *Ifngr2* promoters and p300 recruitment to the *Tap1* and *Psmb8/9* promoters (Fig. 3 *E* and *F*). These promoter regions also exhibited increased H3K14 and K27 acetylation after Oxali treatment (Fig. 3*G*). Oxali-induced H3K14 acetylation at nuclear foci, similar to those revealed by p300 antibody staining, was also observed by immunofluorescence (IF) analysis (*SI Appendix*, Fig. S5*B*). Increased RELA/p65 K310 acetylation, which was attenuated after treatment by p300/CBP inhibitors, was confirmed by IB and IF analyses (*SI Appendix*, Fig. S5 *C* and *D*). Using HA- or Myc-tagged *p300* and Flag-tagged *Stat1* expression vectors followed by immunoprecipitation (IP), we confirmed binding of p300 to endogenous RELA/p65 and transfected STAT1 (*SI Appendix*, Fig. S5*E*), an interaction that stimulates p300 acetyltransferase activity (36). To investigate the basis for p300 nuclear translocation, we examined induction of HLA-B–associated transcript 3 gene product, BAT3, which controls intracellular p300 distribution (37). IF analysis confirmed Oxali-induced nuclear translocation of both p300 and BAT3 (*SI Appendix*, Fig. S5*F*).

### p300 and CBP Control MHC-I AgPPM Expression and Neoantigen Amounts.

**Fig. 4.**
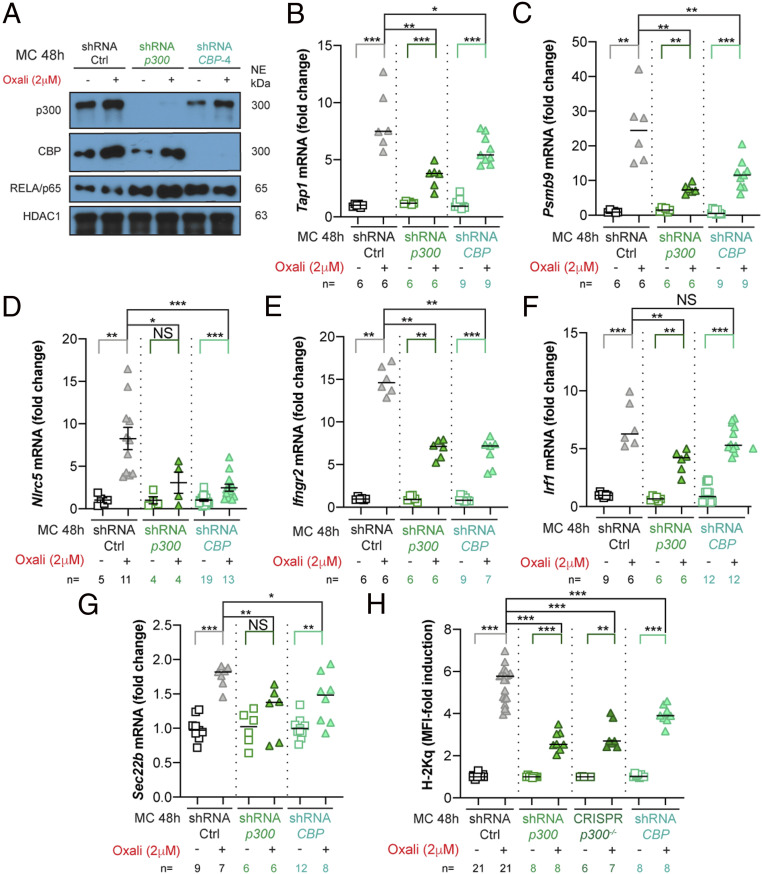
p300 and CBP control Oxali-induced MHC-I AgPPM genes. (*A*) Parental (shRNA-Ctrl) and *p300* or *CBP* silenced Myc-CaP cells were incubated with Oxali for 48 h. Nuclear extracts were IB analyzed for p300, CBP, p65/RELA, and HDAC1 (loading control). (*B*–*G*) RNA expression in above cells was analyzed by qRT-PCR with the indicated primers. (*H*) Parental and gene-edited Myc-CaP cells were incubated with Oxali and analyzed for surface MHC-I (H-2Kq) expression by flow cytometry. Two-sided *t* test (means ± SEM), and Mann–Whitney test (median) were used to determine significance unless indicated otherwise. *P < 0.05; **P < 0.01; ***P < 0.001; NS, not significant. Specific *n* values are shown in *B*–*H*. Each experiment includes at least three biological replicates.

We generated cell lines deficient in p300 or CBP (Fig. 4*A* and *SI Appendix*, Fig. S6*B*). p300^Δ^-Myc-CaP cells expressed CBP and up-regulated its expression upon Oxali treatment, and CBP^Δ^-Myc-CaP cells behaved similarly. Notably, p300^Δ^-Myc-CaP cell viability did not differ from that of parental cells and neither p300 nor CBP ablation reduced total or Oxali-induced total RELA/p65 protein or *Nfkbia* mRNA (Fig. 4*A* and *SI Appendix*, Fig. S6 *A*–*C*). However, *Tap1*, *Psmb9*, *Nlrc5*, *Infgr2*, and *Ifna* mRNA inductions were attenuated by both p300 or CBP ablations, whereas *Irf1* and *Erap1* mRNA inductions were only reduced in p300^Δ^-Myc-CaP cells (Fig. 4 *B*–*F* and *SI Appendix*, Fig. S6 *D* and *E*). Both the p300 and CBP deficiencies attenuated induction of *Sec22b* mRNA (Fig. 4*G*), coding for a vesicle-trafficking protein that regulates phagosomal maturation and antigen cross-presentation (38). Consequently, both deficiencies hampered Oxali- and Mito-induced H-2Kq mRNA and surface expression (Fig. 4*H* and *SI Appendix*, Fig. S6 *F* and *G*). A p300/CBP inhibitor also attenuated H-2Kq protein, and *Psmb9* and *Tap1* mRNA inductions (*SI Appendix*, Figs. S6 *H* and *I*). Conversely, treatment of Myc-CaP cells with nonlethal doses of the HDACi panobinostat (LBH589) induced *Nlrc5*, *Psmb9*, and *Tap1* mRNAs and surface H-2Kq (*SI Appendix*, Fig. S6 *J*–*L*).

**Fig. 5.**
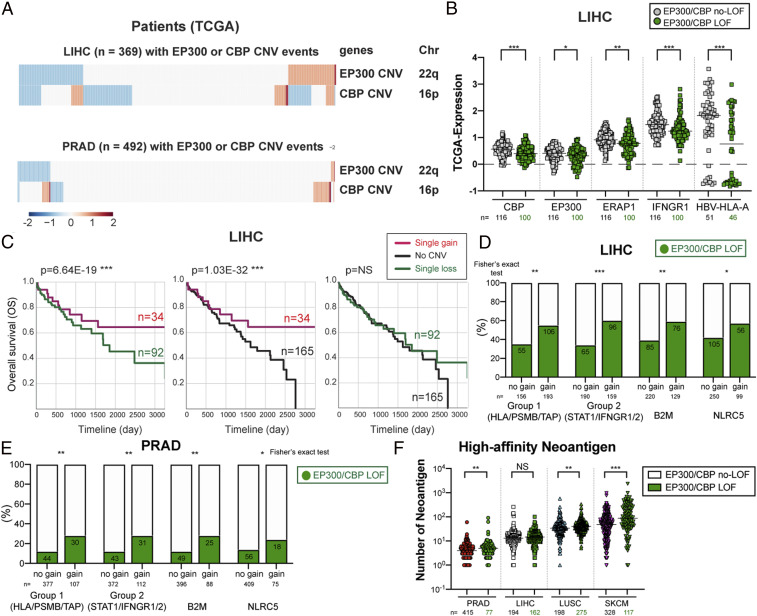
p300/CBP control MHC-I AgPPM expression in human cancers. (*A*) *EP300* and *CBP* CNV losses and gains in LIHC (*n* = 369) and PRAD (*n* = 492). Key: dark blue (homozygous deletion), light blue (one copy loss), white (no CNV), pink (one copy gain), and red (high amplification). (*B*) Comparison of immune gene expression between LIHC *EP300/CBP* LOF and non-LOF groups. Nonviral patients are included for comparison of *CBP*, *EP300*, *ERAP1*, and *IFNGR1* expression. HLA-A expression was compared between *EP300/CBP* LOF and non-LOF HBV-infected groups. (*C*) Kaplan–Meier survival curves of LIHC patients with single gain (*n* = 34), no CNV (*n* = 165), and single loss (*n* = 92) events in *EP300/CBP*. *P* values are based on log rank test. (*D* and *E*) Enrichment comparison of LIHC (*D*) and PRAD (*E*) with or without *EP300/CBP* LOF or no CNV versus CNV gain in the indicated gene groups: MHC-I AgPPM (HLA/PSMBs/TAPs), STAT1/IFNGR1/IFNGR2, β2M, and NLRC5. Fisher’s exact test was used to determine significance. (*F*) Landscape of high-affinity neoantigens in four tumor types: PRAD (*n* = 492), LIHC (*n* = 356), lung squamous cell carcinoma (LUSC; *n* = 473), skin cutaneous melanoma (SKCM; *n* = 445). Groups were separated into *EP300/CBP* LOF (green dots) and non-LOF for neoantigen analysis. Mutations receiving a rank score of <2 and <0.5 were considered binding and strong binding, respectively. *P* values are based on Wilcoxon rank-sum test. Expression of neoantigens was determined from TCGA mRNA-seq reads. Two-sided *t* test (means ± SEM), and Mann–Whitney test (median) were used to determine significance unless indicated otherwise. One-way ANOVA analysis and multiple comparison confirmed the results. *P < 0.05; **P < 0.01; ***P < 0.001; NS, not significant. Specific *n* values are shown in *A*–*F*. Each experiment includes at least three biological replicates.

To gather information about p300 and CBP in human cancer, we examined The Cancer Genome Atlas (TCGA) dataset and found significant correlations between *EP300* or *CBP* mRNAs and genes identified by our integrative RNA-seq and ATAC-seq analyses, including *RELA*, *STAT1*, *NFKB1*, *IFNGR2*, and *NLRC5* (Fig. 5 *A* and *B* and *SI Appendix*, Fig. S7*A*). Similar correlations were found between histone modifiers and genes involved in MHC-I AgPP and T-cell inflammation, particularly in human liver cancer (*SI Appendix*, Fig. S7 *B* and *C*). Cancers with *EP300/CBP* loss of function (LOF; deletion and/or copy number variants [CNV] loss) showed lower *ERAP1* and *IFNGR1* or HLA-A expression (Fig. 5*B*). Curiously, EP300 and CBP were described both as oncogenes and oncosuppressors (39, 40). Phenotypes associated with heterozygous alterations were described in B cell lymphoma and Rubinstein-Taybi syndrome 1 (41, 42), suggesting dosage-dependent EP300/CBP function. We found that multiple CNVs, both gains and losses, affected *EP300* and *CBP* in liver hepatocellular carcinoma (LIHC) and prostate adenocarcinoma (PRAD) (Fig. 5*A* and *SI Appendix*, Table S1) and correlated with their expression (*SI Appendix*, Fig. S7*D*). Interestingly, increased *EP300* and *CBP* copy numbers correlated with improved LIHC patient survival (Fig. 5*C*). We also analyzed CNVs of genes involved in MHC-I AgPPM and IFNγ signaling (Fig. 5 *D* and *E* and *SI Appendix*, Fig. S8*A* and Table S1). We found that LIHC and PRAD patients with gains in MHC-I AgPPM genes showed more frequent *EP300/CBP* LOF (Fig. 5 *D* and *E* and *SI Appendix*, Table S1), suggesting a compensatory mechanism that allows cancers with elevated MHC-I AgPPM to evade immune recognition. We also analyzed the number and the fraction of neoantigens in different cancers (*SI Appendix*, Fig. S8 *B* and *C*). Remarkably, cancers with *EP300/CBP* LOF showed higher neoantigen amount (Fig. 5*F* and *SI Appendix*, Fig. S8*D*), supporting the notion that tumor immunoediting shapes the neoantigen landscape (43, 44) and that EP300/CBP may be part of this process.

### NF-κB Signaling and MHC-I AgPPM Induction.

**Fig. 6.**
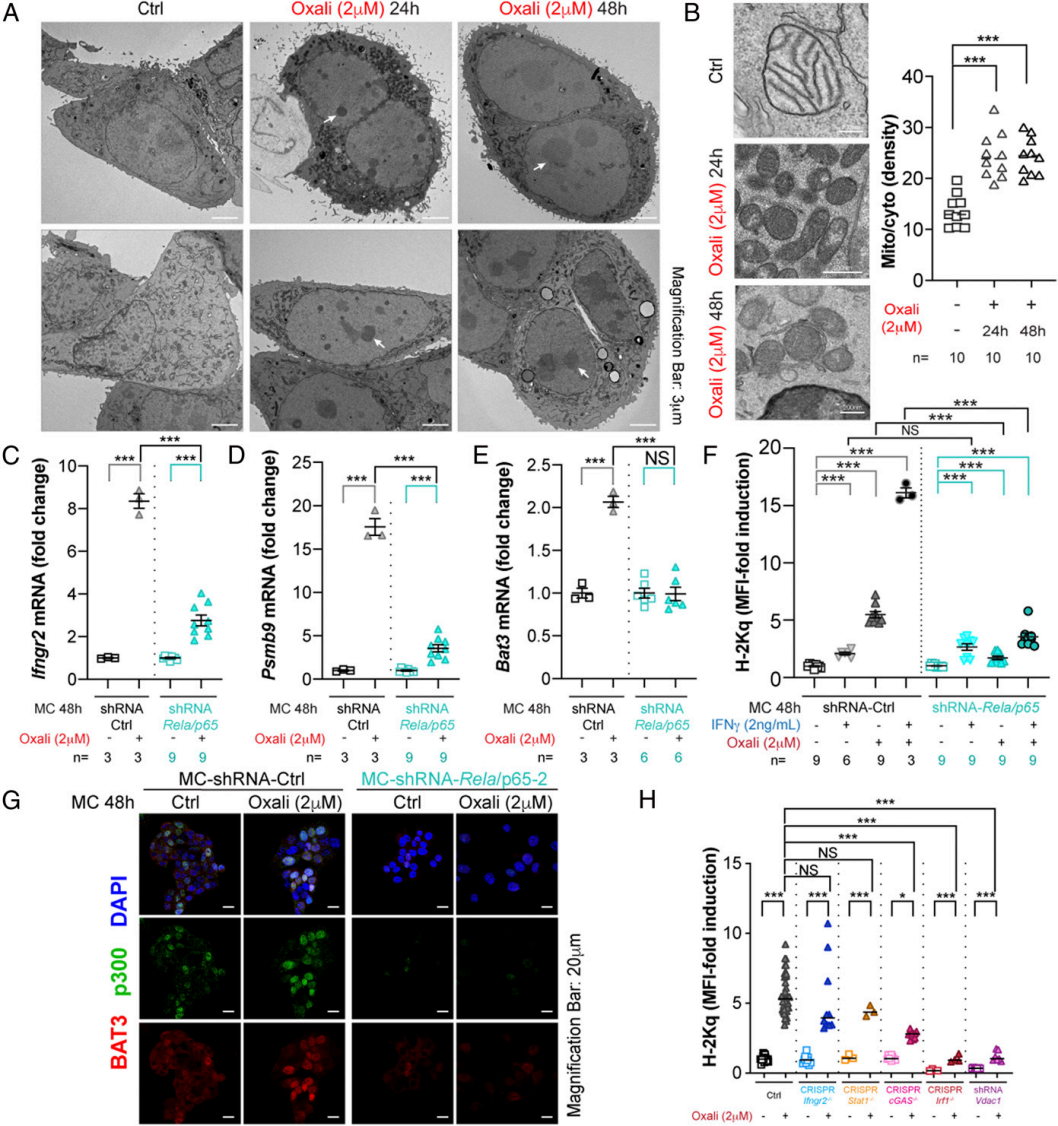
Role of NF-κB signaling in Oxali-induced MHC-I AgPPM expression. (*A* and *B*) Myc-CaP cells treated as indicated were fixed and examined by electron microscopy. Magnification bars are indicated in each image. White arrows indicate nucleolar stress. Ten representative images from each treatment group were analyzed and mitochondria/cytoplasm ratios were determined (*B*, *Right*). (*C*–*E*) Parental (shRNA-Ctrl) or *Rela/p65*-silenced Myc-CaP cells were incubated with Oxali as indicated. RNAs were analyzed by qRT-PCR with the indicated primers. The results were confirmed using three different *Rela/p65* shRNAs. (*F*) Myc-CaP cells described in *C* were treated as indicated and analyzed for surface MHC-I (H-2Kq) expression. (*G*) Parental or *Rela/p65*-silenced Myc-CaP cells were treated as indicated and stained with mouse anti-p300 (green) and rabbit anti-BAT3 (red). Nuclei were counterstained with DAPI (blue) (*n* = 3). (Scale bar: 20 μm.) (*H*) Parental and gene edited (Ctrl, CRISPR-Cas9) or shRNA silenced Myc-CaP cells were treated as indicated and analyzed for surface MHC-I (H-2Kq) expression. (*B*, *F*, and *H*) Two-sided *t* test (means ± SEM), and Mann–Whitney test (median) were used to determine significance. One-way ANOVA and multiple comparisons were used to confirm significance. *P < 0.05; **P < 0.01; ***P < 0.001; NS, not significant. Specific *n* values are shown in *B*–*F*. Each experiment includes at least three biological replicates.

Electron microscopy (EM) suggested that Oxali-treated cells underwent nucleolar/ribosomal and mitochondrial stress indicated by the condensed appearance of both organelles (Fig. 6 *A* and *B* and *SI Appendix*, Fig. S9*A*). However, the absence of nuclear or mitochondrial fragmentation confirmed that most of the stressed cells remained viable. Oxali-induced nucleolar/ribosomal stress (45, 46), which was confirmed by mass spectrometry (MS) and RNA-seq analyses (*SI Appendix*, Fig. S9 *B*–*E*), can account for NF-κB activation (47). To determine NF-κB’s role in the response to Oxali, we generated RELA/p65-deficient cell lines (*SI Appendix*, Fig. S10 *A* and *B*). Consistent with the ChIP experiments shown above (Fig. 3*E*), RELA/p65 was needed for full induction of *Ifngr2*, *Tap1*, *Psmab9*, *Nlrc5*, and *Bat3* mRNAs by low-dose Oxali (Fig. 6 *C*–*E* and *SI Appendix*, Fig. S10 *C* and *D*). RELA/p65 ablation strongly inhibited surface H-2Kq induction by Oxali but barely affected the response to IFNγ (Fig. 6*F*). BAT3 and p300 nuclear translocation was also attenuated in RELA-deficient cells (Fig. 6*G* and *SI Appendix*, Fig. S10*E*). Treatment of Myc-CaP cells with IKKβ inhibitors also reduced H-2Kq surface expression (*SI Appendix*, Fig. S10*F*). We also generated IRF1-, STING/cGAS-, STAT1-, IFNγR2-, and VDAC1-deficient Myc-CaP cells (Fig. 6*H* and *SI Appendix*, Fig. S11 *A*–*E*). VDAC1 (voltage-dependent anion channel 1) was recently shown to be required for the cytoplasmic release of mitochondrial (mt) DNA (48), which is considerably elevated in Oxali-stressed cells (*SI Appendix*, Fig. S11 *E* and *F*). Notably, VDAC-1 ablation strongly reduced *Rela*, *p300*, and *Irf-1* mRNA induction by Oxali (*SI Appendix*, Fig. S11*G*). Ablation of VDAC1 and IRF1, but not STAT1 or IFNγR2, abrogated Oxali-induced expression of surface H-2Kq and MHC-I AgPPM genes (Fig. 6*H* and *SI Appendix*, Fig. S11 *H*–*K*). Ablation of cGAS led to a small decrease in H-2Kq expression and no effect on induction of most AgPPM genes (Fig. 6*H* and *SI Appendix*, Fig. S11 *H*–*K*). Not surprisingly, STAT1, IRF-1, and IFNγR2 as well as VDAC1 and cGAS were required for H-2Kq surface expression in Myc-CaP cells treated with IFNγ alone or IFNγ + Oxali (*SI Appendix*, Fig. S11*L*). Oxali treatment also led to modest induction of PD-L1, a response that was enhanced by exogenous IFNγ and was IRF1 dependent (*SI Appendix*, Fig. S11*M*), which has previously been shown to contribute to efficacy of ICIT (49). PD-L1 induction was not affected by TAP1 ablation, which completely prevented H-2Kq surface expression.

### Chemotherapy-Induced Functional Antigen Presentation.

To confirm that Oxali stimulates neoantigen presentation, we used MS to determine the peptidomes of H-2Kb and H-2Db molecules isolated from MC-38 cells after treatments, as described previously (50). Treatment with IFNγ + Oxali induced higher amounts (based on area under the curve) of H-2Kb–bound peptides relative to Oxali or IFNγ alone (*SI Appendix*, Fig. S12*A*). Although IFNγ led to higher amounts of H-2Db–bound peptides than Oxali in this particular cell line, chosen for its high MHC-I expression, some peptides were more efficiently presented after IFNγ + Oxali treatment.

The T-cell activating ability of the Oxali-induced MHC-I bound peptides was confirmed using TRC2 PCa cells expressing high-, medium-, and low-affinity ovalbumin (Ova) variants. Oxali treatment stimulated H-2Kb presentation of the SIINFEKL epitope, especially in TRC2-N4 cells made to express the high-affinity (wild-type [WT]) variant (*SI Appendix*, Fig. S12*B*). When incubated with OT-I CD8^+^ T cells, whose T-cell receptor (TCR) is SIINFEKL specific, Oxali-treated TRC2-N4 cells were more readily killed by activated OT-I T cells (*SI Appendix*, Fig. S12 *C* and *D*). OT-I T cells enhanced presentation of the WT SIINFEKL epitope by TRC2-N4 cells in the absence of Oxali but had no effect on cells expressing the medium (TRC2-G4)- or low (TRC2-E1)–affinity variants. These results are consistent with a previous publication showing that only the high-affinity SIINFEKL epitope induces IFNγ secretion by OT-I cells (51), and further establish that the effect of Oxali is mechanistically distinct from that of IFNγ and dependent on neoantigen affinity and TCR activation.

We examined mouse and human cancer cell lines that differ in basal MHC-I expression. As described above, cells with high basal MHC-I such as MC-38 and B16 melanoma showed a weak response to platinoids alone but that response, including *Nlrc5* mRNA and surface MHC-I, was augmented by IFNγ (*SI Appendix*, Fig. S13 *A* and *B*). In other cancer cells, e.g., the mouse melanoma YUMM cell lines, we observed a considerable variation in the response (*SI Appendix*, Fig. S13*C*). Strong Oxali-induced MHC-I surface expression was detected in the human PC3 PCa cell line, PCSD1 cells, a three-dimensional (3D) organoid culture from a patient-derived xenograft (PDX) of bone metastatic PCa, certain primary melanoma cells, and MIA PaCa-2 cells, representing ICIT-refractory PDAC (*SI Appendix*, Fig. S13 *D*–*G*).

### Activation of p300/CBP and NF-κB Is Needed for Oxali + Anti–PD-L1 Synergy.

**Fig. 7.**
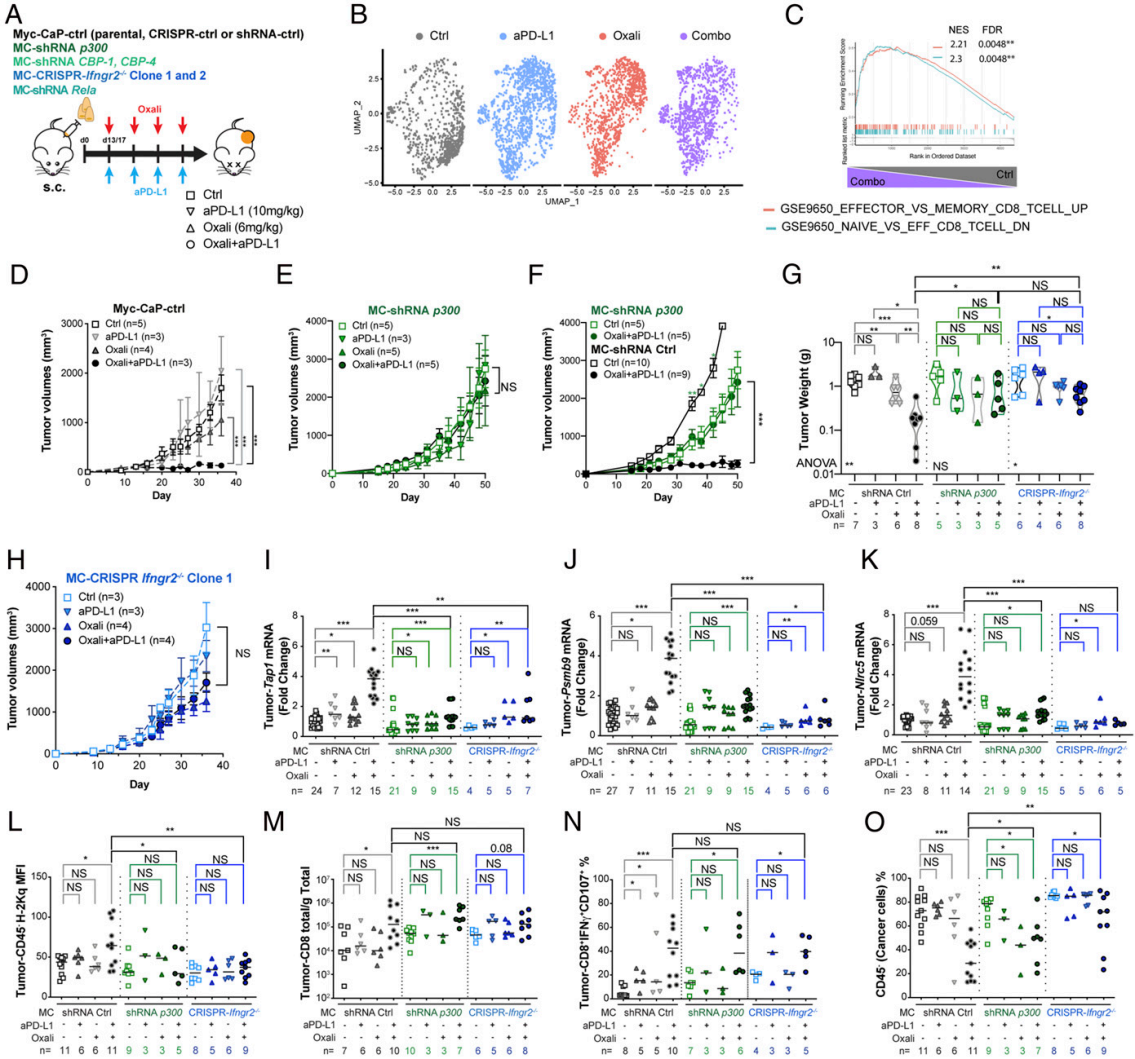
Oxali + anti–PD-L1 synergy depends on p300/CBP and IFNγR2 expression. (*A*) Schematic description of in vivo experiments. Mice bearing s.c. Myc-CaP tumors were allocated into four treatment groups: 1) control, 2) Oxali (6 mg/kg; weekly), 3) anti-PD-L1 (10 mg/kg; weekly), and 4) Oxali + anti–PD-L1 (weekly). After four treatment cycles, during which tumor size was measured, the mice were killed and analyzed. (*B*) Uniform manifold approximation and projection (UMAP) representation of total T-cell populations profiled by scRNA-seq. Eleven distinct clusters were identified (*SI Appendix*, Fig. S14 *B* and *C*). Proportional contributions of each cluster to sample-specific T-cell populations is shown. Total cell numbers in each cluster are noted in *SI Appendix*, Fig. S14*D*. (*C*) Enrichment plots for candidate pathways defining CD8^+^ Teff over Tmem and Tnaïve cells. Enrichment plots for the comparison between control and combo treatment is shown (other comparisons are shown in S14H). (*D*, *F*, and *H*) Mice bearing s.c. Myc-CaP tumors generated by control (*D*), *p300*-silenced (*E* and *F*), and *Ifngr2* ablated (clone 1) (*H*) cells were treated as described in *A*. Specific *n* values are shown in each panel. Transient Cas9 expression and stable shRNA transfectants were used to avoid immune responses. Each dot is a treatment group, mean ± SEM. (*G*) Tumor weights for the indicated experimental groups (*n* = 3 to 8). (*D* and *H*) Two-sided *t* test (means ± SEM) and two-way ANOVA were used to determine significance. (*I*–*K*) Total tumor RNA was analyzed by qRT-PCR for expression of the indicated genes. (*L*–*N*) Single-cell tumor suspensions were analyzed for H-2Kq (*L*) expression on CD45^−^ cells, total number of TI-CD8^+^ (*M*) and effector CD8^+^IFNγ^+^CD107^+^ T-cell subsets (*N*). (*O*) Solid tumor composition (% cancer cells) was determined by gating on CD45^−^ cells. (*G* and *I*–*O*) Each dot represents a mouse. Mann–Whitney test (median) was used to determine significance. One-way ANOVA and multiple comparisons were used to confirm significance. Specific *n* values are shown in *D*–*O*.

We sorted tumor-infiltrating CD8^+^ T cells (TI-CD8^+^) from subcutaneous (s.c.) Myc-CaP tumors, treated with either Oxali, anti–PD-L1, Oxali + anti–PD-L1 (combo), or left untreated (control [Ctrl]) and performed single-cell (sc)RNA-seq (Fig. 7*A* and *SI Appendix*, Fig. S14*A*). Several clusters of TI-CD8^+^ cells with distinguishable gene expression and cluster-specific pathway enrichment patterns were detected (Fig. 7*B*, and *SI Appendix*, Fig. S14 *B*–*H*). Notably, elevated *Gzmb*, *Gzam*, *Prf1*, and *Tbx21(Tbet)* mRNAs were detected in TI-CD8^+^ from combo-treated mice (*SI Appendix*, Fig. S14*E*). Only combo therapy was associated with a significantly higher Teff signature (Fig. 7*C* and *SI Appendix*, Fig. S14 *F*–*H*).

**Fig. 8.**
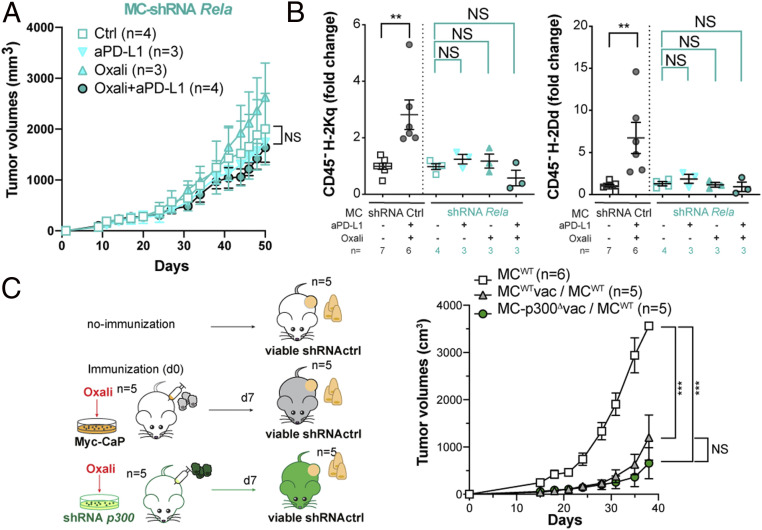
Oxali-enhanced immune rejection requires NF-κB signaling. (*A*) Mice bearing s.c. Myc-CaP tumors generated by control and *RelA*-silenced cells were treated and analyzed as described in Fig. 7*A*. Each dot represents a treatment group mean ± SEM. (*B*) Single tumor cell suspensions were analyzed for H-2Kq (*Left*) and H-2Dd (*Right*) expression on CD45^−^ cells. (*C*) Scheme of vaccination experiments (*Left*). Two groups of mice were immunized with lysates of Oxali-killed shRNA-ctrl (MC^wt^) or *p300*-silenced (MC-p300^Δ^) Myc-CaP cells. After 7 d, mice were s.c. inoculated with live shRNA-ctrl (MC^wt^) cells. Live shRNA-ctrl (MC^wt^) cells were also implanted into nonimmunized mice as a control. Tumor growth curves are shown (*Right*). (*A*–*C*) Two-sided *t* test (means ± SEM) and two-way ANOVA were used to determine significance. *P < 0.05; **P < 0.01; ***P < 0.001; NS, not significant. Specific *n* values are shown in *A*–*C*.

Next, we examined the involvement p300, CBP, IFNγR2, and NF-κB/RelA in Oxali-enhanced and CTL-mediated rejection of Myc-CaP tumors (Fig. 7*A*). The synergistic inhibition of tumor growth by Oxali + anti–PD-L1 was completely abrogated by p300 and CBP ablation in Myc-CaP cells (Fig. 7 *D*–*G* and *SI Appendix*, Fig. S15*A*). IFNγR2 ablation also abolished the response to Oxali + anti–PD-L1 (Fig. 7 *G* and *H* and *SI Appendix*, Fig. S15*B*). As found in vitro, low-dose Oxali induced expression of *Ifngr2*, *Tap1*, *Psmb9*, and *Nlrc5* mRNA in Myc-CaP tumors (Fig. 7 *I*–*K* and *SI Appendix*, Fig. S15*C*). PD-L1 blockade did not affect *Ifngr2* mRNA expression, although it potentiated *Tap1*, *Psmb*9, and *Nlrc5* mRNA induction by Oxali, probably through IFNγ secretion by reinvigorated CTLs (Fig. 7 *I*–*K* and *SI Appendix*, Fig. S15*C*). Indeed, IFNγR2 ablation had little effect on the response to Oxali alone while abrogating the response to Oxali + anti–PD-L1. *Tap1*, *Psmb9*, and *Nlrc5* induction by Oxali or Oxali + anti–PD-L1 was abrogated after p300 ablation (Fig. 7 *I*–*K*). IFNγR2 and p300 ablation also attenuated therapy-induced MHC-I (H-2Kq and H-2Dd) surface expression on CD45^−^ cancer cells (Fig. 7*L* and *SI Appendix*, Fig. S15 *D* and *E*), but had no effect on PD-L1 expression (*SI Appendix*, Fig. S15*F*). IFNγR2 and p300 ablations also had no effect on H-2Kq expression by tumor-infiltrating CD11c^+^ dendritic cells (*SI Appendix*, Fig. S15*G*). In accordance with scRNA-seq data, the Oxali + anti–PD-L1 combo increased the percentage and/or total numbers of tumor-infiltrating CD8^+^ and CD4^+^ T cells, CD107^+^IFNγ^+^ CTLs, IFNγ^+^ CD8^+^, and TNF^+^IFNγ^+^ CD8^+^ T cells, CD8^+^CD44^+^ Teff cells, and CD8^+^CD44^+^PD1^+^TIM-3^+^ T cells analyzed by flow cytometry (Fig. 7 *M* and *N* and *SI Appendix*, Fig. S16 *A* and *C*–*H*). Similar results were obtained for splenic CD8^+^ T cells (*SI Appendix*, Fig. S16 *B* and *I*–*K*). Of note, p300 or IFNγR2 ablation had little effect on tumor-infiltrating effector CD8^+^ T cells, whose numbers were similarly increased after Oxali + anti–PD-L1 treatment in p300- or IFNγR2-expressing and nonexpressing tumors (Fig. 7 *M* and *N* and *SI Appendix*, Fig. S16 *C*–*H*). By contrast, the Oxali + anti–PD-L1 combo decreased the fractions of each tumor occupied by CD45^−^ “cancer” cells, an effect that was most pronounced in WT tumors relative to p300 or IFNγR2 ablated tumors (Fig. 7*O*). NF-κB/RelA ablation also abolished the response to Oxali + anti–PD-L1 (Fig. 8*A*), consistent with its requirement for MHC-I and IFNγR2 induction (Fig. 8*B* and *SI Appendix*, Fig. S17*A*), Thus, Oxali-induced up-regulation of MHC-I AgPPM genes in malignant cells is important for the final recognition and killing stage of the cancer-immunity cycle (25) but has no role in ICIT-induced CTL reinvigoration.

Of note, ICD-mediated T-cell priming proceeded normally in the absence of p300. FVB mice were immunized with Oxali-killed p300-proficient or -deficient Myc-CaP cells and challenged 1 wk later with vital p300-proficient or -deficient Myc-CaP cells (Fig. 8*C* and *SI Appendix*, Fig. S17*B*). Myc-CaP cells grew significantly slower in FVB mice immunized with either p300-proficient or -deficient Myc-CaP cells compared to nonvaccinated mice, indicating that p300 has no effect on ICD-mediated T-cell priming.

## Discussion

ICIT-induced tumor rejection depends on activation of the cancer-immunity cycle, initiated by priming of tumor-directed T cells and terminated by killing of the targeted cancer cells by effector CTLs (16, 25). T-cell priming can be enhanced by certain chemotherapeutic drugs capable of inducing ICD (14) and ICIT (4, 27). Nonetheless, even highly effective T-cell priming and ICIT do not ensure successful CTL-mediated tumor killing, which requires MHC-I–mediated presentation of tumor-specific antigens (2, 14, 17, 52). Many cancers, especially PCa (*SI Appendix*, Fig. S7*E*) (53), evade immune elimination by down-regulating MHC-I molecules or essential AgPPM components (11). Here we show that two different chemotherapeutic drugs, Oxali and Mito used at rather low concentrations, enhance CTL-mediated cancer cell recognition and killing through transcriptional induction of MHC-I AgPP (schematic summary, *SI Appendix*, Fig. S17*C*). Although induction of MHC-I antigen presentation by chemotherapy and radiotherapy was described (54–56), the underlying mechanisms were only partly explored and attributed to type I IFN signaling. However, recent reports showing that sustained type I IFN signaling contributes to anti–PD-(L)1 resistance (57, 58) cast doubt on the role played by type I IFN in chemotherapy- or radiotherapy-induced immune stimulation. Our results show that Oxali renders low MHC-I–expressing PCa cells responsive to anti–PD-(L)1 therapy through transcriptional activation of the MHC-I AgPPM by NF-κB and p300/CBP, but not via the IFN-responsive TF STAT1. Ablation of p300 (or CBP) or NF-κB/RelA abolished the ability of low-dose Oxali to synergize with anti–PD-L1 and induce rejection of Myc-CaP tumors. Consistent with their direct involvement in transcriptional activation of MHC-I AgPPM genes, ablation of p300 or RelA abrogated *Tap1*, *Psmb9*, and *Nlrc5* induction in Myc-CaP tumors, but had no effect on tumor infiltration by effector CD8^+^ cells. Tumor-infiltrating CTLs, however, were strongly increased after anti–PD-L1 + Oxali treatment as indicated by scRNA-seq and flow cytometry. In contrast, ablation of p300 had no effect on the ability of Oxali-killed Myc-CaP cells to prime antitumor immunity, as indicated by vaccination experiments.

Oxali treatment triggers nucleolar/ribosomal stress (45, 46), possibly through its preferential interaction with rDNA or inhibition of rRNA synthesis, which represents almost half of the human genome (59). By virtue of its highly repetitive nature, rRNA integrity and expression are also sensitive to loss of topoisomerase II activity (60), a sequelae of Mito treatment. EM analysis of Oxali-treated Myc-CaP cells confirmed altered nucleolar morphology, consistent with nucleolar stress, which can trigger NF-κB activation (47). By inducing *Bat3* transcription, RelA/NF-κB supports p300/CBP nuclear translocation, further increasing its own activity and stimulating histone acetylation. Oxali treatment can also enhance NF-κB activity via mitochondrial stress, whose presence in Myc-CaP cells is suggested by increased mitochondrial density and appearance of fragmented mtDNA in the cytosol. Ablation of VDAC1, through which mtDNA exits the mitochondrion (48), reduced *p300* and *Rela* mRNA expression and abrogated induction of NLRC5 and different MHC-I AgPPM components. NF-κB is also needed for induction of IFNγR2. Although IFNγR2 ablation had no effect on Oxali-induced MHC-I surface expression in cultured cells, it abrogated the rejection of Myc-CaP tumors, and inhibited induction of MHC-I AgPPM genes in mice treated with anti–PD-L1 + Oxali. We postulate that IFNγR2 induction in Myc-CaP cells makes them more responsive to IFNγ secreted by tumor-infiltrating CTLs.

Neither Oxali nor Mito were developed as immunostimulatory drugs. It is therefore understandable that their immunogenic activity depends on multiple signaling pathways that are activated on induction of sublethal DNA damage and nucleolar and mitochondrial stress. Given the number of different signaling pathways activated by Oxali or Mito, it is rather surprising that ablation of either p300/CBP or RelA results in almost complete inhibition of the drug-induced immunogenic response. These findings parallel the cardinal importance of p300/CBP and NF-κB in activation of the MHC-I AgPP system. p300 plays a key role in assembly of the NLRC5 transcriptional activation complex and NF-κB recruitment to MHC-I genes (61). Notably, down-regulation of NLRC5 has been observed in multiple cancer types, resulting in evasion of immune elimination (12). Conversely, we found that cancers with NLRC5 gain were more likely to undergo *CPB/EP300* loss. We also found that *EP300* and *CBP* LOF mutations and CNVs are rather common in certain types of cancer, and that their presence correlates with reduced MHC-I AgPPM expression. These genetic alterations seem more common than HLA loss mutations. Moreover, hepatocellular carcinoma and PCa with gain of *HLA*, *PSMB*, and *TAP* genes, possibly due to chromosome 6p amplification (62), show higher frequency of *CBP/EP300* loss, which may allow them to undergo immune evasion. Based on its loss in several types of cancer, *EP300* was suggested to behave as a tumor suppressor gene (63, 64). We suggest that *CBP/EP300* loss promotes tumor growth by enabling immune evasion. One way to restore recognition of tumors with monoallelic *EP300/CBP* loss is treatment with low-dose Oxali or Mito or more potent and specific EP300/CBP activators.

## Materials and Methods

Detailed information about the animal models, in vivo and in vitro studies, flow cytometry, qRT-PCR, immunoblot analysis, bioinformatic analysis, statistics, and materials is provided in *SI Appendix*, *Material and Methods* and Table S2.

## Supplementary Material

Supplementary File

## Data Availability

This study did not generate new unique materials. The sequencing data are available in National Center for Biotechnology Information’s Gene Expression Omnibus (GEO) database: mouse ATAC-seq (GSE126287) and RNA-seq (GSE126274). Data from in vitro experiments are available under the GEO accession no. GSE126288. Single-cell RNA-seq data from live CD8^+^CD3^+^ tumor-infiltrating cells are available under the GEO accession no. GSE151611. The results shown here are in part based upon data generated by the TCGA Research Network: https://www.cancer.gov/about-nci/organization/ccg/research/structural-genomics/tcga.

## Change History

March 2, 2021: The license for this article has been updated.
