# Supplementary material for: Activation of NF-κB and p300/CBP potentiates cancer chemoimmunotherapy through induction of MHC-I antigen presentation
Source: Proc Natl Acad Sci U S A. 2021 Feb 18;118(8):e2025840118. doi: 10.1073/pnas.2025840118 (PMC7923353; doi:10.1073/pnas.2025840118)
Supplement: Supplementary File [file pnas.2025840118.sapp.pdf]

PNAS
www.pnas.org

Supplementary Information for

Activation of NF-κB and p300/CBP potentiates cancer chemoimmunotherapy through induction of MHC-I antigen presentation

Yixuan Zhou[1], Ingmar Niels Bastian[1], Mark D Long[1], Michelle Dow[1], Weihua Li[1], Tao Liu, Rachael Katie Ngu, Laura Antonucci, Jian Yu Huang, Qui T Phung, Xi-he Zhao, Sourav Banerjee, Xue-Jia Lin, Hongxia Wang, Brian Dang, Sylvia Choi, Daniel Karin, Hua Su, Mark H. Ellisman, Christina Jamieson, Marcus Bosenberg, Zhang Cheng, Johannes Haybaeck, Lukas Kenner, Kathleen M. Fisch, Richard Bourgon, Genevive Hernandez, Jennie R. Lill, Song Liu, Hannah Carter, Ira Mellman, Michael Karin[2], Shabnam Shalapour [2,3]

[1]contributed equally to this work.

[2]Correspondence: Michael Karin, Shabnam Shalapour. Email: karinoffice@ucsd.edu, sshalapour@mdanderson.org.
[3]Lead Contact

This PDF file includes:

Supplementary text: Material and Methods
Figures S1 to S15
Tables S1 to S2

Material and Methods

Animal models

C57BL/6 and *FVB/N* control mice were purchased from Charles River Laboratories. *OT-I* mice (Taconic) were used for cell culture experiment. All mice were maintained in filter-topped cages on autoclaved food and water at the UCSD animal facility and were used in accordance with UCSD and NIH guidelines and regulations, particularly for experimental Neoplasia, which includes tumor size limits (not exceeding 2 cm for a single tumor) and monitoring parameters.

Subcutaneous tumor models

2-3 × 10^6 mouse cancer cells as indicated were subcutaneously (s.c.) injected into the right flank. Tumors were measured every 2-3 days using a caliper. Tumor volumes were calculated as $V = (\text{width}^2 \times \text{length}) / 2$. Male mice were used for prostate cancer (PCa) models and both genders were used for melanoma models. Mouse treatment studies were matched design control trials as described previously (1). Accordingly, mice were randomly chosen and paired based on sex (e.g. male for PCa), age and tumor size. For transplanted tumor models, tumor size was defined by the median tumor volume. An identification code was assigned to each tumor-bearing mouse.

Vaccination experiments

Myc-CaP cells or their genetic-modified variants were treated with 100 µM Oxaliplatin and harvested 48 h later. Aliquots of cells were subjected to flow cytometric analysis to ensure that 90 ± 10% of were dead. Dead cells were subcutaneously inoculated (5x10^6 in 200 µl PBS, pH 7.4) into the lower flank of 8-week-old male FVB mice. Seven days later, 2-3 x 10^6 vital Myc-CaP cells or their genetic-modified variants (in 200 µL PBS, pH 7.4) were inoculated into the contralateral flank, as previously described (2). Mice were then monitored for tumor appearance and tumor size was measured three times a week.

Treatment with chemotherapy or antibodies

Oxaliplatin was diluted in 5% dextrose and i.p. injected weekly at 6 mg/kg as indicated. Anti-PD-L1 antibody was i.p. injected at 10 mg/kg weekly. Mice were treated for 3-5 weeks.

Flow cytometry

Cell suspensions were prepared from prostate, tumor, and spleens as described (3). For lymphocyte isolation, tissues were cut into small pieces and incubated in dissociation solution [DMEM medium supplemented with 5% FBS, collagenase type I (200 U/mL), collagenase type IV (100 U/mL), and DNase I (100 µg/mL)] for 30 min at 37°C. After incubation, cell suspensions were passed through a 40 µm cell strainer and washed twice. To block Fc-mediated interactions, mouse cells were pre-incubated with 0.5-1 µg of purified anti-mouse CD16/CD32 per 100 µL. Isolated cells were stained with labeled antibodies in PBS with 2% FBS and 2 mM EDTA or cell staining buffer (Biolegend). Dead cells were excluded based on staining with Live/Dead fixable dye (FVD-eFluor780, eBioscience). For intracellular cytokine staining, cells were restimulated with cell stimulation cocktail [eBioscience; containing phorbol 12-myristate 13-acetate (PMA) and ionomycin (Ion)], in the presence of a protein transport inhibitor cocktail containing Brefeldin A and Monensin (eBioscience). For CD107a measurement, a staining antibody was added to the culture during stimulation. After 4-5 h, cells were fixed and permeabilized either with BD™ Cytofix/Cytoperm reagent for cytokine staining or eBioscience™ Foxp3/Transcription Factor staining buffer for combined staining of cytokines with transcription factors. After fixation/permeabilization, cells were stained with labeled antibodies of interest. Cells were analyzed on a Beckman Coulter Cyan ADP flow cytometer. Data was analyzed using FlowJo 10.2 software (Treestar). The absolute numbers of particular immune cells in spleens were calculated by multiplying the total splenic cell number by the percentage of the particular cell type. Absolute numbers of particular immune cells in tumors were calculated by multiplying the cell number of the tumor portion by the percentages of the corresponding cell type in total tumor cells divided by the weight of the analyzed tumor fragment (cell number per g).

For *in-vitro* experiments, adherent cells were detached using accutase or non-enzymatic cell dissociation solution (CellStripper Dissociation Reagent), and stained as described above. 4′,6-diamidino-2-phenylindole (DAPI) or Live/Dead fixable dye (FVD-eFluor780, eBioscience) were used to calculate and exclude death cells. Gating strategies of different stainings are provided in Figure S1B and Figure S6J-L.

Cell culture experiments

Mammalian cells (see Key Resource Table) were all grown in a humidified incubator with 5% CO_2 at 37°C. Myc-CaP, HEK293T and MIA PaCa-2 cells were grown in Dulbecco's Modified Eagle Media (DMEM, Gibco) supplemented with 10% FBS, 2 mM L-glutamine, and 100 U/mL penicillin and streptomycin. TRAMP-C2 cells were grown in DMEM supplemented with 5% FBS, 0.4 mM L-glutamine, 100 U/mL penicillin and streptomycin (Gibco), 5% NuSerum IV (Corning), 0.005 mg/mL bovine insulin (Sigma-Aldrich), and 10 nM dehydroisoandrosterone (Acros Organics). MC38, PC3, and all YUMM cells (4) were grown in DMEM/F12 (Gibco) supplemented with 10% FBS, 2 mM L-glutamine, and 100 U/mL penicillin and streptomycin. B16 and WM793 cells were grown in RPMI 1640 (Gibco) supplemented with 5-10% FBS, 0.4 mM L-glutamine, and 100 U/mL penicillin and streptomycin. Human cell lines were cultured as indicated by the provider (Key Resource Table). Inducible ovalbumin (Ova)-P2A-red fluorescence protein (RFP)-expressing TRAMP-C2 cell lines were developed as previously described (3). Ovalbumin processing results in presentation of SIINFEKL peptide on MHCI (H-2Kb), or variants SIIGFEKL (G4) and EIINFEKL (E1). The SIINFEKL peptide can be recognized by OT-I $CD8^+$ T cells that express a high affinity TCR specific for ovalbumin. OT-I cells were isolated from *OT-I* mice (5) with CD8 selection beads and cocultured with TRAMP-C2 cells as previously described (3).

Cells were regularly tested using a mycoplasma PCR kit (EZ-PCR Mycoplasma Test Kit, Biological Industries) and visualized for their characterization. Cells were counted by Trypan Blue (Gibco) exclusion and seeded into 12 or 24 well plates (Flow Cytometry and RNA processing) or into 6 well and 10 cm culture plates for Immunoblot analysis. Cells were treated in fresh media with agents as indicated for 3-48 h after seeding and collected from plates using Acutase (STEMCELL Technologies) for flow cytometry analysis, TRIzol Reagent (Ambion) for RNA processing, or Immunoblot (IB) Lysis Buffer for IB analysis at indicated time points.

qRT-PCR analysis

Total RNA was extracted using an AllPrep DNA/RNA mini Kit (Qiagen) or TRIzol Reagent (Ambion). RNA was reverse transcribed using a Superscript VILO cDNA synthesis kit (Invitrogen) or RTScript cDNA Synthesis Kit (Empirical Bioscience). Quantitative real-time PCR (qRT-PCR) was performed using Sso Advanced Universal SYBR Green Supermix (Biorad) on Biorad CFX96 machine. The relative expression levels of target genes were measured and normalized against levels of housekeeping genes *GAPDH* or *ACTB*. Fold-difference (as relative mRNA expression) was calculated by the comparative C_t method ($2^{(Ct(\text{housekeeping gene} - \text{gene of interest}))}$). Primer sequences were obtained from the following PrimerBank site (https://pga.mgh.harvard.edu/primerbank/) or designed using the following site (https://www.ncbi.nlm.nih.gov/tools/primer-blast/index.cgi) and are listed in Supplementary Table 2.

Nuclear, Cytoplasmic Extraction and Immunoblot (IB) Analysis

NE-Per Nuclear and cytoplasmic extraction kit and EpiQuik Nuclear Extraction Kit I (Epigentek, Farmingdale, NY, USA) were used for acquiring nuclear and cytosolic fractions of cells and tumor tissues and the buffers were prepared according to the manufacturer's protocol. Whole cell or whole tissue lysates were made using IB lysis buffer (20 mM pH 7.5 Tris-HCl, 10 mM EDTA, 150 mM NaCl, 1% Triton, 1% sodium deoxycholate) with protease and phosphatase inhibitors. IB analysis was performed on cell or tissue lysates separated by SDS-PAGE and transferred to PVDF membranes (Millipore and Trans-Blot Turbo). Antibody details are provided in Key Resource Table. For quantitative analysis of IB, ImageJ was used and targeted protein bands were normalized to loading controls.

HAT Activity Assay

HAT activity assays were performed with EpiQuik HAT Activity/Inhibition Assay Kit (Epigentek, Farmingdale, NY, USA). Solutions were prepared according to manufacturer's protocol.

Immunoproteasome activity assay

Immunoproteasome subunit LMP7 activity assays were analyzed directly on cell lysates from Myc-CaP cells using LMP7 specific fluorogenic peptide substrate Ac-ANW-AMC (Boston Biochem) (6). Cells were treated with or without indicated concentration of oxaliplatin (2 μM), cisplatin (2 μM), or carboplatin (4 μM) alone or in combination with IFNγ (2 ng/mL) for 48 h. One set of cells treated with IFNγ alone or in combination with each of the platinoids was treated with 200 nM ONX-0914 (Selleckchem) (7) for 2 h prior to lysis to determine background non-specific AMC fluorescence. Cells were lysed directly in proteasome assay buffer (50 mM Tris-HCl, pH 7.5, 40 mM KCl, 5 mM $MgCl_2$, 1 mM ATP, 0.1% NP-40, phosphatase inhibitors, 1 mM dithiothreitol). 100 μM substrate peptide Ac-ANW-AMC was added to the lysate and reaction was carried out for 30 min-1 h at 30°C in an orbital shaker. Fluorogenic AMC release into solution was assessed by a Tecan M200 plate reader (excitation: 380 nm, emission: 440-460 nm) which provides a direct measurement of LMP7 activity in the cell lysates. The measured LMP7 activity was normalized against total protein concentration of cell lysates and represented as fold LMP7 activity to control untreated Myc-CaP cell lysates. Data are represented as dot scatter plots using Graphpad Prism.

Immunoprecipitation (IP)

293T cells were transfected with EP300 (pCMVβ-p300-myc, pSG5-HA-p300-DY1399, pSG5-HA-p300, Addgene) and STAT1 (Stat1 alpha Flag pRc/CMV, Stat1 beta Flag pRc/CMV, Addgene) overexpression vectors for 24 h and then treated with Oxali (2 μM) for 24 h. Whole cell lysates of 293T cells were incubated with anti-HA magnetic beads (MedChemExpress Cat#HY-K0201) at 4 °C overnight and prepared according to manufacturer's protocol. The HA-tagged protein bound to the beads was denatured and eluted in 1x Laemmli Sampler Buffer (Bio-Rad, Cat#161-0747). The beads were removed from the samples using the magnetic stand. The immunoprecipitants were then IB analyzed.

Chromatin Immunoprecipitation Assays

Cells were crosslinked for 10 min with 1% formaldehyde. The reaction was stopped by 5 min incubation with 0.125 M Glycine. Cells were washed, harvested with PBS supplemented with protease and phosphatase inhibitors, and cytoplasmic membranes lysed with lysis buffer (5 mM PIPES, 85 mM KCl, 0.5% NP40). After centrifugation, nuclei were lysed for 10 min on ice with sonication buffer (1% SDS, 10 mM EDTA, 50 mM TRIS pH 8 supplemented with protease inhibitors) and sonicated to obtain chromatin fragments of about 400-600 nucleotides. The lysates were precleared for 1 h at 4°C with 30 μL of protein A agarose (Upstate), spun at 5000 rpm for 1 min, and then the supernatants were collected and stored at 10% of input. Chromatin diluted with 9 volumes of dilution buffer (0.01% SDS, 1.2 mM EDTA, 16.7 mM Tris HCl pH 8, 1.1% Triton X-100, 167 mM NaCl, protease inhibitors) was incubated overnight with 20 μL of Protein A Dynabeads (Invitrogen) coated with the antibodies as indicated. The day after, the immunocomplexes were washed five times with Buffer A (0.1% SDS, 2 mM EDTA, 20 mM Tris-HCl pH 8, 1% Triton X-100, 150 mM NaCl), four times with Buffer B (0.1% SDS, 2 mM EDTA, 20 mM Tris-HCl pH 8, 1% Triton X-100, 500 mM NaCl), and once with Buffer T.E. (10 mM Tris-HCl pH 8, 1 mM EDTA). After the final wash, the immunocomplexes were eluted twice with 250 μL elution buffer (1% SDS, 100 mM $NaHCO_3$) for 15 min in rotation at RT and, upon addition of 200 mM NaCl, the crosslinking reversed with an overnight incubation at 65°C. After de-crosslinking, the samples were digested with proteinase K (Thermo Fisher Scientific) and RNAse A (Thermo Fisher Scientific) for 2 h at 42°C, and the DNA was purified and precipitated. Eluted DNA was analyzed by real time PCR as previously described (8, 9) using the indicated primers in Key Resource Table.

CRISPR-Cas9 cloning, packaging, transfection

Stable and transient CRISPR/Cas9 plasmids were used for *in vitro* and *in vivo* experiments, respectively. For stable CRISPR/Cas9 plasmid production, 2 μg of the expression lentiCRISPR v2 (Addgene) (10) was digested for 1 h at 37°C with 1 μL BsmBI (NEB) and 5 μL NEBuffer 3.1 (NEB) into a total volume of 60uL and was gel purified (Qiagen). For gRNA insertion, a pair of 25 nt oligos containing the appropriate overhangs were annealed using 1 μL of each primer at a 100 μM stock concentration, 0.5 μL T4 DNA Ligase (NEB), and 1 μL T4 Ligase Buffer (NEB) into a total ligation volume of 10 μL. Primers were annealed using the following parameters: 37°C for 30 min then 95°C for 5 min and ramped down to 25°C at 5°C/min. The oligos were ligated into the vector for 10 min at room temperature by mixing 1 μL of the digested plasmid, 1 μL annealed oligos, 1 μL T4 Buffer (NEB), and 0.5 μL T4 DNA Ligase (NEB) into a total ligation volume of 10 μL. Plasmids were transformed into 25 μL Max Efficiency Stbl2 (ThermoFisher Scientific) bacteria. Resulting colonies were minipreped (Qiagen) and verified by sanger sequencing before transfection into mammalian cells. Transient CRISPR/Cas9 plasmids were constructed exactly as mentioned above using the expression vector pSpCas9(BB)-2A-GFP (PX458) (Addgene) (11) and a BbsI (NEB) restriction enzyme. gRNA sequences were found on Chopchop and DNA oligos were synthetized. The gRNA sequences are shown in Key Resource Table. MAX Efficiency Stabl2 bacteria containing proper CRISPR/Cas9 were expanded in LB broth at 37°C overnight. Plasmids were extracted from bacteria using QIAGEN Plasmid Mini Kit or Maxi Kit.

For stable CRISPR/Cas9, lentivirus was produced in HEK293T cells transfected at 80-90% confluency using Lipofectamine 3000 (Invitrogen) as recommended by the manufacturer and psPAX2 (Addgene) and pMD2.G (Addgene) packaging vectors. Medium was changed 6-8 h after transfection and supernatant was collected after 48-72 h. Viral media was passed through a pre-wetted 0.45 μm PVDF filter (Millipore) and mixed with 10 μg ml^{-1} Polybrene (Sigma Aldrich) before being added to recipient cells. Infected cells were treated with puromycin to generate stable populations.

Transfection with transient CRISPR/Cas9 in Myc-CaP and TRAMP-C2 cells was carried out using Lipofectamine 3000 (Invitrogen) as recommended by the manufacturer. Cells were transfected at 70-80% confluency using 12 μg of the plasmid of interest. Medium was changed 36 h after transfection and cells were sorted 48 h after transfection. Cell sorting was performed on a BD FACS Jazz. For each transfected cell line, at least 4 million cells were collected using 0.5 % Trypsin (Gibco), counted by Trypan Blue exclusion, and resuspended in PBS. Cells were sorted into 96 well plates and GFP negative populations were sorted as control lines. Resulting colonies were expanded and verified by immunoblot analysis, flow cytometry, and qRT-PCR.

shRNA cell lines

Bacteria containing desired shRNA Lentivirus-plasmids were obtained from La Jolla Institute for Immunology (LJI)-Functional Genomics lab. Sequences are shown in Key Resource Table (https://rnai-reagent.liai.org/scrm_mm_trc/). Bacteria were expanded in LB broth overnight at 37°C and plasmids were extracted using Mini-prep or Maxi-prep (QIAGEN) according to manufacturer's instruction. shRNA plasmids were delivered using lentivirus as described above. After lentiviral infection, cells were selected using puromycin and knockdown was confirmed using qRT-PCR and IB.

Immunostaining and Histology

Tissues were embedded in Tissue-Tek OCT compound (Sakura Finetek, Torrance, CA, USA) and snap-frozen. Tissue sections were fixed in cold acetone/methanol or 3-4% PFA for 3-10 min and washed with PBS. Cultured cells were cultured on coverslips and fixed in 4% paraformaldehyde for 10 min at room temperature. After washing twice in PBS, cells were incubated in PBS containing 10% FBS or 0.2% BSA and 1% donkey serum or goat serum for surface staining or added 0.2% gelatin/0.2% BSA (from cold water fish skin; Sigma-Aldrich) or 0.1% saponin for intracellular staining for 15-30 min to block nonspecific sites

of antibody adsorption. Sections were incubated with primary antibodies for 1 h or overnight at RT or 4°C, respectively. After washing, secondary antibodies were added for 1 h at RT. As negative controls, samples were incubated with isotype-matched control antibodies or secondary antibodies only. After staining with DAPI, sections were covered with Vectashield Mounting Medium (Vector Laboratories, Burlingame, CA, USA). Confocal images were captured in multitracking mode on a SP5 confocal microscope (Leica) with 40 x or 63 × Plan Apochromat 1.3 NA objective.

Paraffin-embedded specimens from a total of 118 Prostate cancer patients were integrated into a tissue microarray system (TMA) constructed at the Clinical Institute of Pathology at the Medical University of Vienna (MUV). All of the human prostate specimens used for TMA construction were approved by the MUV Research Ethics Committee (1753/2014), as previously described (1). Human liver biopsies were obtained from the Biobank of the Medical University of Graz. Biopsies were registered in the biobank and kept anonymous. The research project was authorized by the ethical committees of the Medical University of Graz (ref. no. 1.0 24/11/2008). The study protocol was in accordance with the ethical guidelines of the Helsinki Declaration. All human samples were de-identified prior to use in our study.

Paraffin-embedded tissue sections were subjected to de-paraffinization and rehydration, and then were immersed in a pre-heated antigen retrieval water bath with a pH 6.1 citrate buffer or Dako Target Retrieval Solution for 20 min at 95-96°C. Sections were then incubated with antibodies against as indicated, and as previously described (1, 3). All staining was done according to manufacturer's protocols (ImmPRESS, Vector Laboratories). DAB (Vector Laboratories, SK-4100) and ImmPACT Vector Red (Vector Laboratories, SK-5105) were used for detection. Nuclei were lightly counterstained with a freshly made haematoxylin solution then further washed in water. Sections were examined using an Axioplan 200 microscope with AxioVision Release 4.5 software (Zeiss, Jena, Germany) or TCS SPE Leica confocal microscope (Leica, Germany). Sections were imaged under a Hamamatsu 2.0-HT Digital slide scanner (Hamamatsu Photonics, EU, Japan, and USA). For measurement, ImageJ 1.49v was used. Serial cut slides were used to show adjacent tissues. Prostate cancer cells were shown by PSA staining and their HLA abundance was measured according to the PSA positive location. Prostate cancer cells were specifically selected using ImageJ, and mean optical density was obtained and statistically compared among groups. For liver cancer slides, cancer cells were the most abundant cell type and could be identified by their histopathological features.

Transmission Electron Microscopy (EM) and determining Mitochondria/Cytoplasm Density

Myc-CaP cells were plated on poly-l-lysine treated MatTek dishes and treated with Oxali (2 μM) for 24 or 48 h. After treatment, cells were fixed with 2% glutaraldehyde (18426, Ted Pella Inc.) in 0.1 M sodium cacodylate buffer, pH 7.4 (18851, Ted Pella Inc.) containing 2 mM $CaCl_2$ for 5 minutes at 37°C and then incubated on ice for 1 h. Cells were washed with 0.1 M sodium cacodylate and posted fixed with 2% osmium tetroxide (19150, Electron Microscopy Sciences) in 1.5% potassium ferrocyanide, 1 mM $CaCl_2$ and 0.1 M sodium cacodylate buffer, pH 7.4 for 30 minutes on ice and washed with double distilled water (ddH_2O) at RT. Cells were then treated with 0.5% thiocarbohydrazide solution using a 0.22 μm Millex 33 mm PES sterile filter (SLGSR33RS, Sigma-Aldrich) and incubated for 10 minutes at RT. Cells were then washed with ddH_2O, treated with 2% osmium tetroxide in ddH_2O for 30 minutes and rinsed with ddH_2O. The plate was treated with 1% aqueous uranyl acetate (22400, Electron Microscopy Sciences) and incubated at 4°C overnight. Cells were then washed with ddH_2O and treated with en-bloc Walton's lead aspartate staining for 5 minutes at 60°C. Afterwards, cells were washed with ddH2O at RT followed by an ice cold graded dehydration ethanol series of 20%, 50%, 70%, 90%, 100% (anhydrous) and then washed with 100% (anhydrous) at RT. Cells were infiltrated with one part Durcupan ACM epoxy resin (44610, Sigma-Aldrich) to one part anhydrous ethanol for 30 minutes then with 100% Durcupan resin, a final change of Durcupan resin and immediately placed in a vacuum oven at 60°C for 48 h. Cells were identified, cut out by jewel saw and mounted on dummy blocks with Krazy glue. Coverslips were removed and 70-80 nm specimen sections were created with a Leica Ultracut UCT ultramicrotome and Diatome Ultra 45° 4 mm wet diamond knife.

Sections were picked up with 50 mesh gilder copper grids (G50, Ted Pella, Inc). The sections were imaged by FEI Spirit transmission electron microscope at 80kV. Images and montages were collected by a Tietz TemCam F-224 2k by 2k CCD camera and by Serial EM software version 3.1.1a. Mitochondrial volume to cytoplasm volume ratio was collected at a resolution of 11.5 nm per pixel. Mitochondria and cytoplasm counting was done using Adobe Photoshop CS5 Extended version 12.0X64. Image resolution for setting the grid spacing was 160. Images were then reset to 2048 by 2048 pixels. Counting was done using the number of grid cross-hairs that fell on mitochondria and on the cytoplasm. Nuclei were not counted. Mitochondria/cytoplasm density was calculated by dividing mitochondria counts by cytoplasm counts X100. Mean and standard deviation were used for t-test analysis.

cytosolic mitochondria DNA (mtDNA) quantification

Cells were treated with Oxali (2 μM) for 48 h. Total DNA was isolated using Allprep DNA/RNA Mini Kit (QIAGEN) according to manufacturer's protocol. mtDNA was measured by qRT-PCR using primers specific for the mitochondrial D-loop region or a specific region of mtDNA that is not inserted into nuclear DNA (non-NUMT). Tert and β2m were used for normalization. To quantify the cytosolic released mtDNA, cytosolic fractions were depleted from mitochondria by Mitochondria Isolation Kit for Cultured Cells (Thermo Fisher Scientific) following manufacturer's protocol as described previously (12). The quality of the cytosolic fractions were confirmed by IB analysis using VDAC antibodies (Figure S4L). The amount of mtDNA in cytosolic fraction were measured using qRT-PCR, as described above.

Organoid cultures from Patient-derived xenograft (PDX) model of bone metastatic PCa

This study was carried out in strict accordance with the recommendations in the Guide for the University of California San Diego (UCSD) Institutional Review Board (IRB). Approval was received from the UCSD institutional review board (IRB) to collect surgical specimens from a patient for research purposes. A surgical PCa bone metastasis specimen was harvested from a patient who had progressed to castrate resistant bone metastatic prostate cancer and labelled as Prostate Cancer San Diego 1 (PCSD1) for the purpose of de-identification of patient data, as described previously (13). PCSD1 cells were injected into the femur endosteal space of Rag2$^{-/-}\gamma_c^{-/-}$ male mice to establish a patient-derived xenograft (PDX) model representing a preclinical model of bone metastatic PCa as previously shown (13). PCSD1 cells were maintained as intra-femoral tumors in male Rag2$^{-/-}\gamma_c^{-/-}$ mice prior to establishing 3D organoid cultures. All experiments involving xenograft models were conducted under an IACUC approved protocol at the University of California, San Diego. Tumors were processed according to previously established methods (13) with the additional step of immuno-depletion of mouse cells (Miltenyi Biotec) to enrich for human cells. PCSD1 3D organoid cultures were established as previously described (14) with modifications as described in Mendoza and Lee et al. PCSD1 cells were resuspended in growth factor reduced Matrigel (Corning/Fisher) at a concentration of 25,000 cells per 20 μL of Matrigel and organoids treated with Oxali after one week.

Single cell RNA-seq (scRNA-seq) processing and analysis

Tumors from the same treatment groups were pooled (n = 7-8 / group). Tumor single cell suspensions were prepared as decribed in the flow cytometry section. Isolated cells were stained with labeled antibodies for CD45-PE, CD4-FITC, CD8a-PE-Cy7 and CD3-APC in cell staining buffer (Biolegend). Dead cells were excluded with Live/Dead fixable dye (FVD-eFluor780, eBioscience). CD45$^+$CD3$^+$CD8$^+$ T cells were sorted in PBS with 0.5% BSA and 2 mM EDTA. Droplet-based 3' end massively parallel single-cell RNA sequencing (scRNAseq) was performed by encapsulating sorted live CD8$^+$CD3$^+$ tumor infiltrating T cells into droplets and libraries prepared using Chromium Single Cell 30 Reagent Kits V3 according to manufacturer's protocol (10x Genomics).

Raw sequence data demultiplexing, barcode processing, alignment and filtering were performed using the Cell Ranger Single-Cell Software Suite (v3.1.0). Subsequent filtering and downstream analyses were

performed using Seurat (15). Genes expressed in less than 3 cells and cells that express less than 300 genes were excluded from further analyses. Additional filtering of cells was determined based on the overall distribution of mitochondrial gene expression (< 10%) to eliminate dying cells, respectively. Assessment and removal of multiplets was performed using Scrublet (16). Normalization and variance stabilization of remaining data was applied using regularized negative binomial regression (sctransform (17)), including regression of mitochondrial gene expression prior to principle component analysis (PCA). Optimal dimensionality of the dataset was decided after examination of the JackStraw procedure and Elbow plot. The FindNeighbors function was utilized that implements a graph based nearest neighbor clustering approach, and the FindClusters function was used to identify final cell clusters. UMAP was applied for non-linear dimensional reduction to obtain a low dimensional representation of cellular states. Differential expression between clusters or between samples was determined using the MAST method via the FindMarkers function, using a minimum expression proportion of 25% and a minimum log fold change of 0.25. Unbiased cell type annotation was performed using SingleR (18). Briefly, this framework allows for the annotation of scRNA-seq data to reference transcriptome data sets (ImmGen) of known origin to infer the cellular state of each input cell. Cell annotations in combination with marker gene expression were used to eliminate non-immune and myeloid lineage cells so that subsequent analyses included only T-cell populations. Normalized expression was used for subsequent analyses including heatmap visualization and pathway analysis. Functional pathway enrichment analysis was performed on cluster specific marker gene expression (enrichR) or at the individual cell level (AUCell), and GSEA (clusterProfiler) was used to interrogate pathway enrichment based on differential expression analyses. Query pathways included all hallmark, canonical and gene ontology (GO: Biological Processes) pathways available through MSigDB.

RNA-seq processing and analysis

For *in vitro* experiments, RNA-seq data was processed and analyzed as described below. Quality control (QC) of sequencing reads was performed using FastQC (v0.11.9) (Andrews et al., 2010). Sequencing reads were aligned to the mouse genome build GRCm38 using annotations from GENCODE (vm12) with the splice-aware RNA-seq aligner STAR (v2.7.3a) (20, 21). Following alignment, the raw counts relative to genes were generated by featureCounts (v2.0.0). The RSeQC (v3.0.1) was used to evaluate the quality of alignments and complete QC report compiled with MutliQC (v1.8). Raw feature counts were normalized and differential expression analysis performed using DESeq2 (22). Significance of differential expression was defined by using an adjusted p-value cut-off of 0.05 after multiple testing correction. Gene set enrichment analysis was performed from rank ordered differential expression using the clusterProfiler (23) package in R. Within sample pathway activity was determined by gene set variation analysis (GSVA) (24). Gene sets queried for functional enrichments included the Hallmark, Canonical pathways, and GO Biological Processes Ontology collections available through the Molecular Signatures Database (MSigDB) (25). Transcriptional regulator analysis on the top 500 determined differentially expressed genes was performed using LISA (26).

ATAC-seq processing and analysis

Permeabilized nuclei were obtained by resuspending cells in 250 µL Nuclear Permeabilization Buffer [0.2% IGEPAL-CA630 (Sigma-Aldrich), 1 mM DTT (Sigma-Aldrich), Protease inhibitor (Roche), 5% BSA (Sigma-Aldrich) in PBS (Thermo Fisher Scientific)], and incubating for 10 min on a rotator at 4°C. Nuclei were then pelleted by centrifugation for 5 min at 500 xg at 4°C. The pellet was resuspended in 25 µL ice-cold Tagmentation Buffer [33 mM Tris-acetate (pH = 7.8) (Thermo Fisher Scientific), 66 mM K-acetate (Sigma-Aldrich), 11 mM Mg-acetate (Sigma-Aldrich), 16 % DMF (EMD Millipore) in Molecular biology water (Corning)]. An aliquot was then taken and counted by hemocytometer to determine nuclei concentration. Approximately 50,000 nuclei were resuspended in 20 µL ice-cold Tagmentation Buffer and incubated with 1 µL Tagmentation enzyme (Illumina) at 37°C for 30 min with shaking 500 rpm. The tagmentated DNA was purified using MinElute PCR purification kit (Qiagen). The libraries were amplified using NEBNext High-

Fidelity 2X PCR Master Mix (New England Biolabs, NEB) with primer extension at 72°C for 5 min, denaturation at 98°C for 30s, followed by 8 cycles of denaturation at 98°C for 10 s, annealing at 63°C for 30 s and extension at 72°C for 60 s. Amplified libraries were then purified using MinElute PCR purification kit (Qiagen), and two size selection steps were performed using SPRIselect bead (Beckman Coulter) at 0.55X and 1.5X bead-to-sample volume rations, respectively. Each library was then sequenced on an Illumina NextSeq500 or HiSeq4000 to a depth of >= 25 million usable reads pairs (i.e. after mapping, filtering, and elimination of PCR duplicates as described in the data processing section).

We processed the raw fastq files with the ENCODE ATAC-seq pipeline (https://github.com/ENCODE-DCC/atac-seq-pipeline). In detail, we first auto-detected then applied cutadapt "-m 5 -e 0.10" to trim the adapter sequences from the raw fastq files. Then we aligned the trim sequences to mouse reference genome mm10 using bowtie2 (27) with parameter "-X2000 --mm -k 5". Next, the improper mapped, poorly mapped and unmated reads were filtered from the resultant raw bam files using samtools (27) view with parameter "-F 1804 -q30". Then, duplicates were marked by Picard (27) "MarkDuplicate" and removed by samtools "-F 1804". In the end, the final bam files were acquired after removing mitochondrial reads, sorted, and indexed with samtools. For each condition, we pooled the two replicates then used MACS (v2.1.0) (28) with "--shift -75 --extsize 150 -p 0.01 --nomodel --keep-dup all" to call the accessible regions with enriched ATAC-seq signals. Peaks were further filtered against the ENCODE blacklist regions (29).

To find differentially accessible regions (DARs), we first merged the called regions from all conditions, then used htseq-count (30) to generate the read count in each region for each condition and each replicate. The count table was subsequently processed with DESeq2 (22) to call the DARs. DARs were called using an absolute log2 fold change cut-off of 1 and a false discovery rate (FDR) cut-off of 10^{-5}. We specifically called the DARs: 1) between IFNγ-1 ng/ml vs Control; 2) between Oxaliplatin-2 µM vs Control; 3) between Cisplatin-2 µM vs Control; and 4) between Combo of IFNγ-1 ng/ml + Oxaliplatin-2 µM vs Control. Additionally, we tried to identify the 'additive' effect of combination treatment by using a reduced cut-off (log2 fold change of 0.585 and FDR of 0.05) to call DARs between Combo and Oxaliplatin only and between Combo and IFNγ only. These DARs were then intersected with those identified between Combo vs Ctrl. In another words, the 'additive' effect only occurs when the combination caused significant chromatin accessibility changes over control or either individual treatments alone.

In order to find potential transcription factor binding events within DARs, we utilized GIGGLE (31) to query the complete mouse transcription factor ChIP-seq dataset collection (6,751 datasets across 570 transcription factors) in Cistrome DB (32). For each ChIP-seq dataset in Cistrome DB, we asked how many DARs overlap with the top 1,000 most significant peaks of that dataset, then calculate a 'Giggle score' equal to the product of the -log10 p-value and log2 odds-ratio from a Fisher's Exact two-tailed test. We queried the DARs of IFNγ vs Ctrl, Oxaliplatin vs Ctrl, Cisplatin vs Ctrl, Combo vs Ctrl, and additive DARs separately.

EP300 and CBP LOF analysis

TCGA MAF and CNV files for PRAD, LIHC, LUSC, and SKCM were downloaded directly from TCGA Genomic Data Commons (GDC) Data Portal in July, 2019 and May, 2020, respectively. Patients were labelled as LOF if they fit one or both criteria: (1) they had at least one CNV loss event, and/or (2) they had a LOF mutation in either *CREBBP (CBP)* or *EP300*. We defined LOF mutations as nonsense, frameshift, inframe insertion, inframe deletion, splice site/ splice region mutations and missense mutations not associated with increased *EP300/CBP* expression. Specifically, the mean of z-scored *CREBBP(CBP)* and *EP300* expression was calculated for each patient and the distributions were compared between patients with and without any *p300/CBP* mutations. Missense mutations that resulted in a mean expression level greater than the 75th percentile of non-mutated patients were considered GOF and excluded from the LOF analysis. In the end, the numbers of LOF patients in PRAD (n = 497), LIHC (n = 369), LUSC (n = 486), and SKCM (n = 448) were 79, 170, 280, and 120, respectively.

Human TCGA expression and survival analysis

To compare tumor and normal gene expression in liver and prostate cancer, raw human RNAseq fastq files were downloaded from The Cancer Genome Atlas (TCGA) for samples with clinical and molecular annotations (33). In total, TCGA data from 369 liver tumors (LIHC) and 50 tissue-matched normal samples, 497 prostate tumors (PRAD) and 52 tissue-matched normal samples, 486 lung tumors (LUSC), and 448 skin tumors (SKCM) were downloaded. Transcript levels were quantified by Sailfish v.7.4 in Transcripts per Million (TPM), and we then constructed a matrix describing gene expression (TPM) for each tumor type (LIHC and PRAD) with patient as the columns, genes as the rows. Expression matrices were subsequently log2 transformed after adding a pseudocount of 1. The log2 transformed expression values were then z-score transformed for the two tumor types separately. The associated heatmap for selected genes was generated with Python package seaborn heatmap (Figure S3B) and clustermap in Python (Figure S3E). Pearson correlation coefficients and corresponding p-values of gene expressions were calculated and plotted using Python package seaborn jointplot in Python (Figure S3A). Pairwise Pearson correlation coefficients were calculated using Python package pandas corr function (Figure S3A, S3G).

The latest clinical annotations from all TCGA patients were downloaded (34). Overall survival (OS) was used to plot the Kaplan-Meier survival curves of TCGA LIHC patients with different CNV status (Figure 4N) with Python package lifelines. Significance was assessed using the log-rank test from the same Python package.

Neoantigen analysis

HLA genotyping and mutation calling was performed for *HLA-A*, *HLA-B*, and *HLA-C* genes, which encode the human MHC-I complex. TCGA samples available for LIHC, PRAD, LUSC, and SKCM were typed with Broad Institute's Polysolver (35). Missense and small insertions and deletions (indel) mutations were taken from the MAF files described above. We used the netMHCpan4.0 tool (36, 37) to obtain mutation affinity scores for all TCGA patients' HLA alleles. To determine whether a mutation would be effectively bound as a neoantigen to the MHC-I complex, we calculated Patient Harmonic-mean Best Rank (PHBR) scores (38). For binarizing affinity or PHBR scores, we used score cutoffs of <= 2 and <= 0.5 for binding and strong binding MHC-I neoantigens, respectively (36, 38). Mutations with scores > 2 we considered non-binding (36, 38). Expression of neoantigens were estimated using the transcriptomic reads of patient-specific TCGA mRNAseq bam files using bam-readcounts (39). Fraction of neoantigens was calculated as (number of neoantigen)/(total number of mutations). *P* values were calculated using the Wilcoxon ranksum test to determine the significance for each neoantigen attribute (number of neoantigens and fraction of neoantigens) between patients with each tumor type (PRAD, LIHC, LUSC, and SKCM) or between LOF and non-LOF (Figure 4P, Figure S3H, S3I). The Wilcoxon tests implemented in the scipy.stats Python package were used for these analyses.

Mass spectrometry

MHCI peptide profiling was performed for the H-2Db and H-2Kb ligandome of the murine adenocarcinoma cell line MC-38 treated with four different conditions, (1) an untreated control, (2) IFNγ 2 ng/mL, (3) Oxali 4 μM, and (4) a combination of IFNγ 2 ng/mL and Oxali 4 μM. Briefly, 6.7×10^8 cells were lysed, split into two equal groups and MHCI molecules were immunoprecipitated in parallel using two different antibodies: the H-2Kb-specific antibody derived from the Y3 hybridoma, and the H-2Db-specific antibody from the B22.249 clone, as described previously (40); both were crosslinked to Protein A sepharose resin (Repligen) via DMP chemistry (Thermo Fisher Scientific). After overnight immunoprecipitation at 4°C, MHCI peptides were eluted from the antibody-resin with 0.1 M acetic acid/0.1% trifluoroacetic acid and purified via solid phase extraction (Empore C18) before mass spectrometric analysis. Peptides were loaded onto a trapping column, washed and eluted onto a 75 μm analytical column, both were packed with Luna C18 resin (Phenomenex) and separated by reversed-phase chromatography (nanoAcquity UPLC system, Waters, Milford, MA) using a 120 min gradient. The gradient, composed of solvent A (0.1% formic acid in water)

and solvent B (0.1% formic acid in acetonitrile), went from 5% to 25% B in 90 min, 25% to 50% B in 20 min and 50% to 90% B in 10 min. The flow rate was 350 nL/min. The eluted peptides were analyzed by data-dependent acquisition (DDA) in EThcD mode on a Fusion Lumos mass spectrometer (Thermo Fisher Scientific). Mass spectral data was acquired using methods comprising of a full scan (survey scan) of high mass accuracy in the Orbitrap at 60,000 FWHM resolution followed by MS/MS scans at 15,000 FWHM resolution. The instrument was run with a 3s cycle from MS and MS/MS. Data were processed through the MaxQuant software v1.5.3.17. Andromeda database search results were filtered at the 1% PSM false discovery rate (FDR) and allowing for one unique peptide per protein.

MS analysis on differentially expressed proteins between Oxaliplatin-treated and control Myc-CaP cells was performed using SWATH strategy. Proteins were extracted from control and Oxaliplatin-treated Myc-CaP cells after 24 h using lysis buffer (50 mM Hepes, 6 M urea, 2 M thiourea and 1× protease inhibitor cocktail), and then digested by trypsin (Promega, sequence grade) using FASP (Filter Aided Sample Preparation). The resulting peptides were analysed with an AB Sciex 5600+TripleTOF mass spectrometer (Concord, Ontario, Canada) interfaced to an EkspertTMNanoLC 425 system (Dublin, CA). Data acquisition parameters and SWATH-MS Data Analysis method were described previously [Scientific RepoRts | 7:45913 | DOI: 10.1038/srep45913], except that proteins were identified by searching mass data against UniProt mouse instead of the human database (containing 50190 sequences). Proteins with significant expression level changes after treatment with adjusted $p < 0.05$ and FC $\geqslant$ 1.2 or FC $\leqslant$ 1/1.2 in at least two out of three biological replicates were regarded as differentially expressed proteins and subjected to further pathway enrichment analysis.

Quantification and statistical analysis

Data is presented as either mean ± s.e.m. or median of continuous values and was analyzed by two-sided Students' t-test or Mann-Whitney test for comparison of two groups, respectively D'Agostino & Pearson test and/or Shapiro-Wilk test were used to test the normality of sample distribution. One-way ANOVA or Kruskal-Wallis test was used to compare three or more groups data analyses. Bonferroni's or Dunn's multiple comparison test was applied to compare all pairs of groups. Fisher's exact Chi-square t test was used to calculate statistical significance of categorical values between groups. Two-way ANOVA test was used for tumor growth analysis. Two tailed *P* values of ≤ 0.05 were considered significant. Linear regression was used to determine the correlation between two different variables. Power calculation was used to confirm ($p < 0.05$ with a 95% probability, two-tailed) the accurate sample size. Experiments were repeated independently at least two or three times with similar results. GraphPad Prism software was used for statistical analyses.

SI Figures

Fig. S1

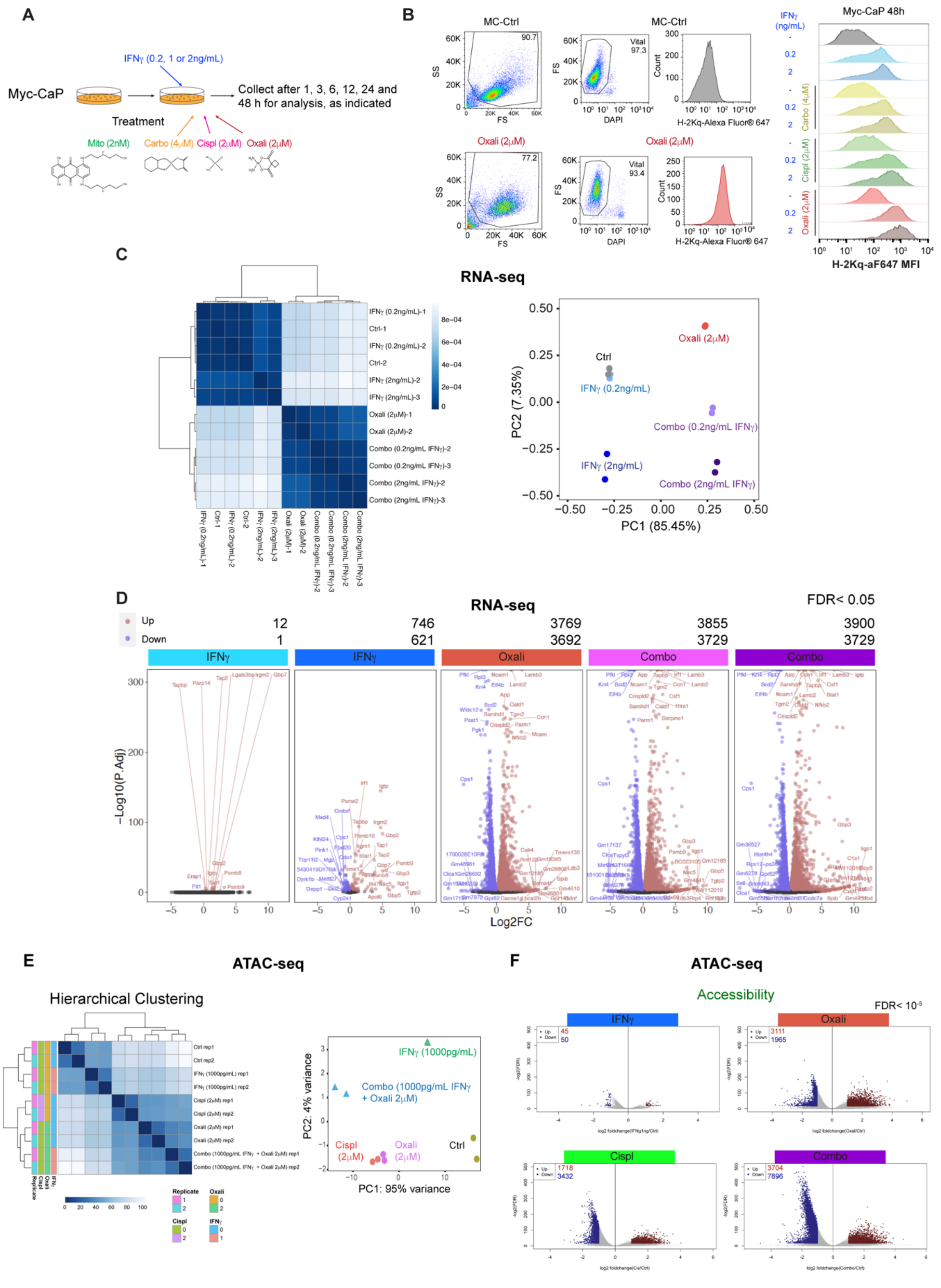

Fig. S1. RNA-seq and ATAC-seq analysis of cancer cells treated with chemotherapeutics**.** (A) A schema describing the cell culture experiments. (B) Flow cytometric analysis confirming induction of MHC-I expression in viable cells subjected to the indicated treatments. (C) Distance (Pearson correlation) matrix (left) and PCA analysis (right) determined from bulk RNA-seq data. Duplicates were used for each condition. (D) Volcano plots depicting DEG analyses that compare each treatment to control. The number of significantly (FDR < 0.05) upregulated (red) and downregulated (blue) genes is shown. (E) Distance (Pearson correlation) matrix (left) and PCA analysis (right) determined from bulk ATAC-seq data. Duplicates were used for each condition. (F) Volcano plots depicting DAR analyses that compare each treatment to control. The number of significantly (FDR < 10^{-5}) opened (red) and closed (blue) regions is shown. Two-sided t-test (means ± s.e.m), and Mann–Whitney test (median) were used to determine significance. *P < 0.05; **P < 0.01; ***P < 0.001; NS, not significant.

Fig. S2

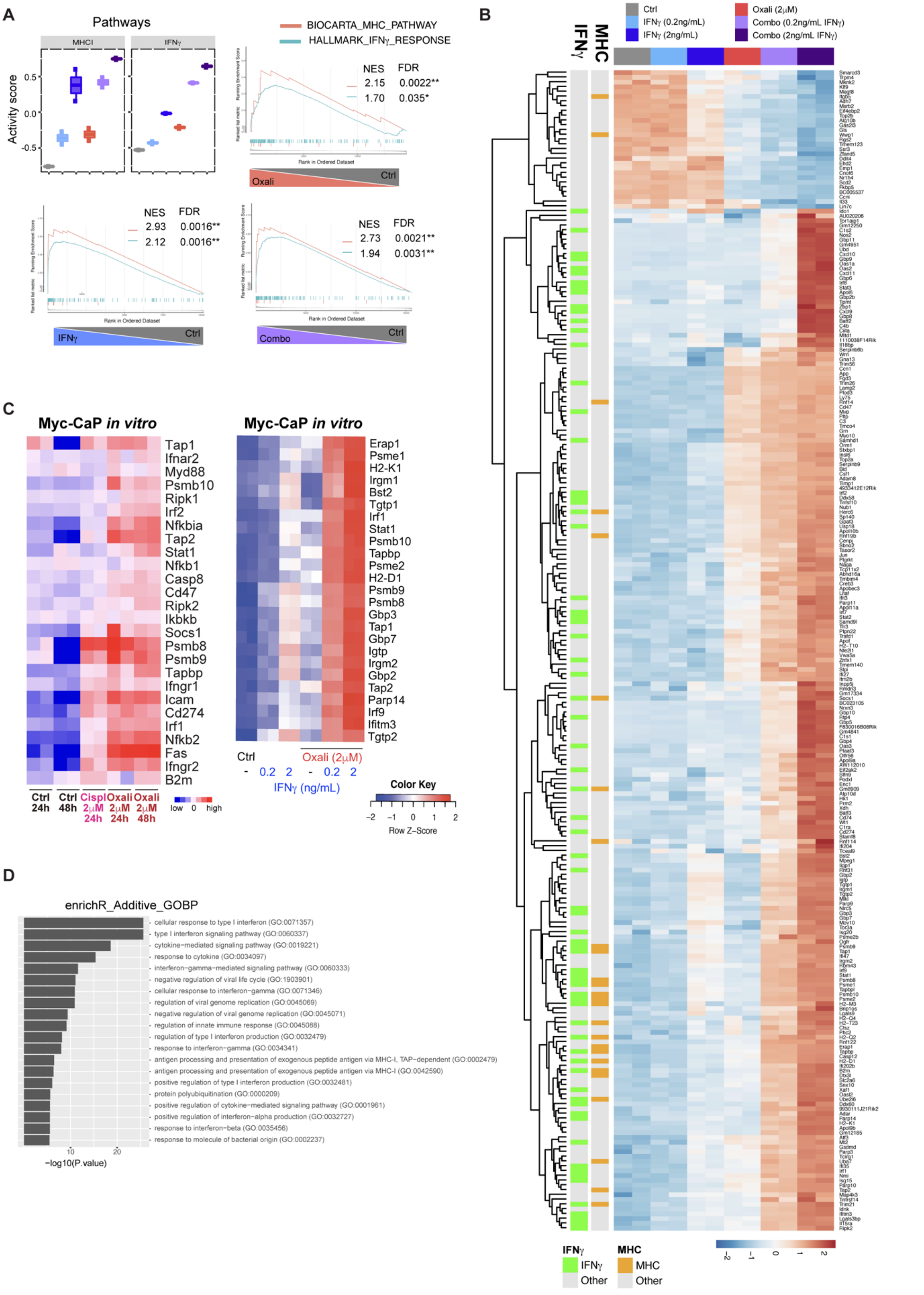
A
Pathways
MHCI
IFNγ
Activity score
0.5
0.0
-0.5
BIOCARTA_MHC_PATHWAY
HALLMARK_IFNγ_RESPONSE
NES FDR
2.15 0.0022**
1.70 0.035*
Oxali
Ctrl
NES FDR
2.93 0.0016**
2.12 0.0016**
IFNγ
Ctrl
NES FDR
2.73 0.0021**
1.94 0.0031**
Combo
Ctrl
Rank in Ordered Dataset
B
IFNγ
MHC
Ctrl
IFNγ (0.2ng/mL)
IFNγ (2ng/mL)
Oxali (2μM)
Combo (0.2ng/mL IFNγ)
Combo (2ng/mL IFNγ)
IFNγ
IFNγ
Other
MHC
MHC
Other
C
Myc-CaP in vitro
Tap1
Ifnar2
Myd88
Psmb10
Ripk1
Irf2
Nfkbia
Tap2
Stat1
Nfkb1
Casp8
Cd47
Ripk2
Ikbkb
Socs1
Psmb8
Psmb9
Tapbp
Ifngr1
Icam
Cd274
Irf1
Nfkb2
Fas
Ifngr2
B2m
Ctrl 24h
Ctrl 48h
Cispl 2μM 24h
Oxali 2μM 24h
Oxali 2μM 48h
low 0 high
Myc-CaP in vitro
Erap1
Psme1
H2-K1
Irgm1
Bst2
Tgtp1
Irf1
Stat1
Psmb10
Tapbp
Psme2
H2-D1
Psmb9
Psmb8
Gbp3
Tap1
Gbp7
Igtp
Irgm2
Gbp2
Tap2
Parp14
Irf9
Ifitm3
Tgtp2
Ctrl
Oxali (2μM)
- 0.2 2 - 0.2 2
IFNγ (ng/mL)
Color Key
-2 -1 0 1 2
Row Z-Score
D
enrichR_Additive_GOBP
cellular response to type I interferon (GO:0071357)
type I interferon signaling pathway (GO:0060337)
cytokine-mediated signaling pathway (GO:0019221)
response to cytokine (GO:0034097)
interferon-gamma-mediated signaling pathway (GO:0060333)
negative regulation of viral life cycle (GO:1903901)
cellular response to interferon-gamma (GO:0071346)
regulation of viral genome replication (GO:0045069)
negative regulation of viral genome replication (GO:0045071)
regulation of innate immune response (GO:0045088)
regulation of type I interferon production (GO:0032479)
response to interferon-gamma (GO:0034341)
antigen processing and presentation of exogenous peptide antigen via MHC-I, TAP-dependent (GO:0002479)
antigen processing and presentation of exogenous peptide antigen via MHC-I (GO:0042590)
positive regulation of type I interferon production (GO:0032481)
protein polyubiquitination (GO:0000209)
positive regulation of cytokine-mediated signaling pathway (GO:0001961)
positive regulation of interferon-alpha production (GO:0032727)
response to interferon-beta (GO:0035456)
response to molecule of bacterial origin (GO:0002237)
10
20
-log10(P.value)

Fig. S2. Chemotherapy induces MHC-I AgPPM genes. (A) Enrichment plots for candidate pathways defining IFNγ response and MHC-I complex expression in treated cells relative to control (Top left). For these pathways, concordant GSVA was applied to each sample to reveal sample specific enrichment patterns. (B) Heatmap depicting scaled expression of genes identified as additive (expression upon combination treatment significantly different from individual treatments and control). Genes included in the IFNγ response (green) and MHC (orange) pathways are noted. (C) RNA from Myc-CaP cells treated as indicated was subjected to RNA-seq analysis. Relative expression of genes involved in antigen presentation is depicted by heat map representation. Duplicates were used for each time point (24 and 48 h). (D) Functional enrichment was applied to genes classified with additive response to combination therapy. The top 20 enriched GO: Biological Processes and Pathways are shown.

Fig. S3

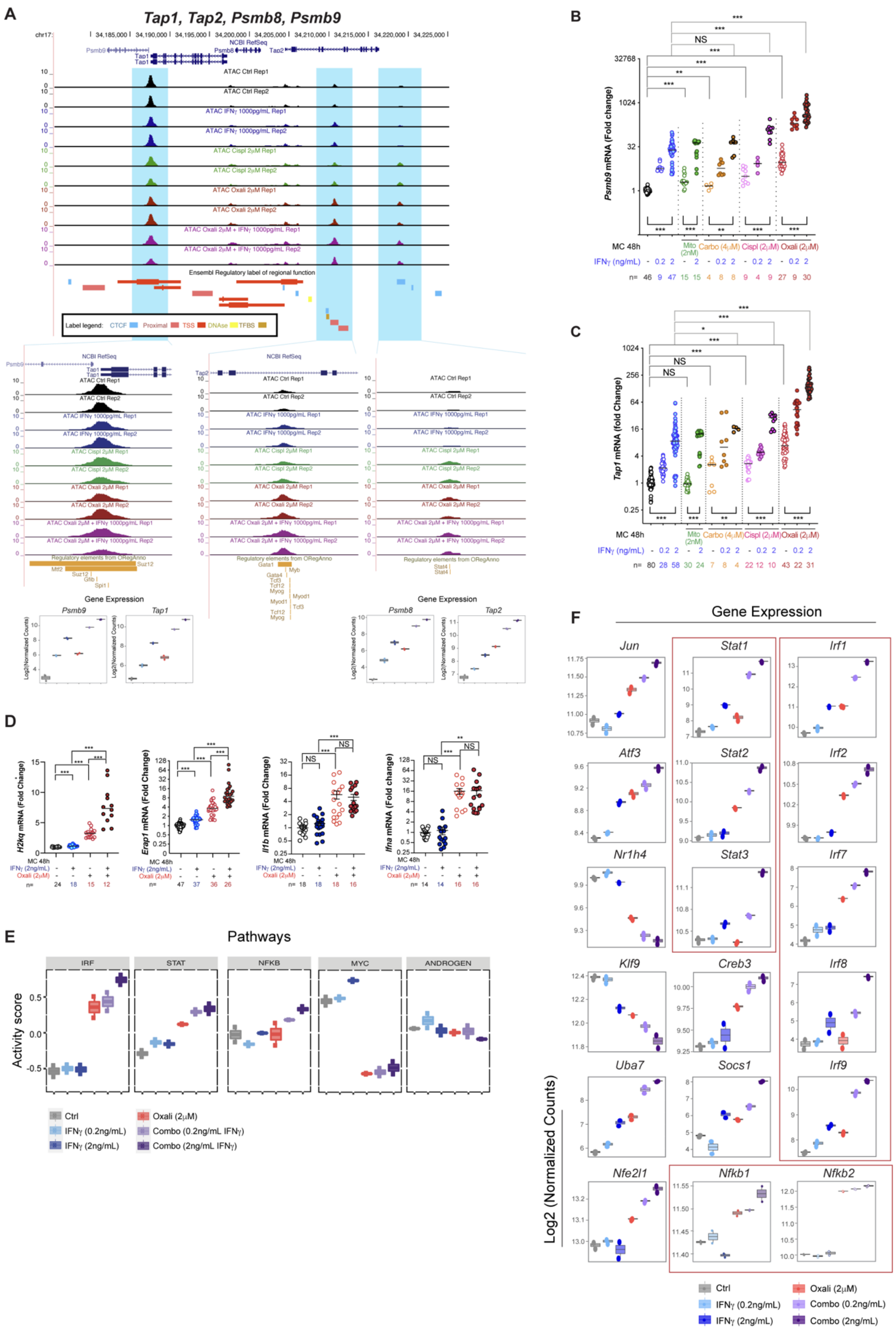

Fig. S3. Oxaliplatin and Mitoxantrone induce MHC-I AgPPM genes. (A) Candidate genomic loci for *Tap1*, *Tap2*, *Psmb8*, and *Psmb9*, showing library-size normalized read pair pileup profiles determined by ATAC-seq across samples (top). Expression of respective genes determined by RNA-seq is also shown (bottom). Three regions with differential accessibilities are highlighted. The literature-curated known regulatory elements from ORegAnno database are shown. (B-C) RNAs from Myc-CaP cells (n = 4-80) incubated as indicated with IFNγ, Mito, Oxali, Carbo, or Cispl for 48 h were analyzed by qRT-PCR using primers for *Psmb9* (B) and *Tap1* (C). (D) RNA from Myc-CaP (MC) cells, treated as indicated, was analyzed by qRT-PCR using primers for *H2kq, Erap1, Il1b* and *Ifna.* (E) GSVA was applied to each sample to reveal sample specific enrichment patterns of candidate pathways (IRF = GRANDVAUX_IRF3_TARGETS_UP, STAT = GO_RECEPTOR_SIGNALING_PATHWAY_VIA_STAT, NFKB = BIOCARTA_NFKB_PATHWAY, MYC = HALLMARK_MYC_TARGETS_V1, ANDROGEN = HALLMARK_ANDROGEN_RESPONSE). (F) Gene expression (RNA-seq) for candidate genes classified with additive response. Genes include TF and other molecules involved in MHC-I AgPP. Two-sided t-test (means ± s.e.m), and Mann–Whitney test (median) were used to determine significance. $^*P < 0.05$; $^{**}P < 0.01$; $^{***}P < 0.001$; NS, not significant. Specific n values are shown in (B-D).

Fig. S4

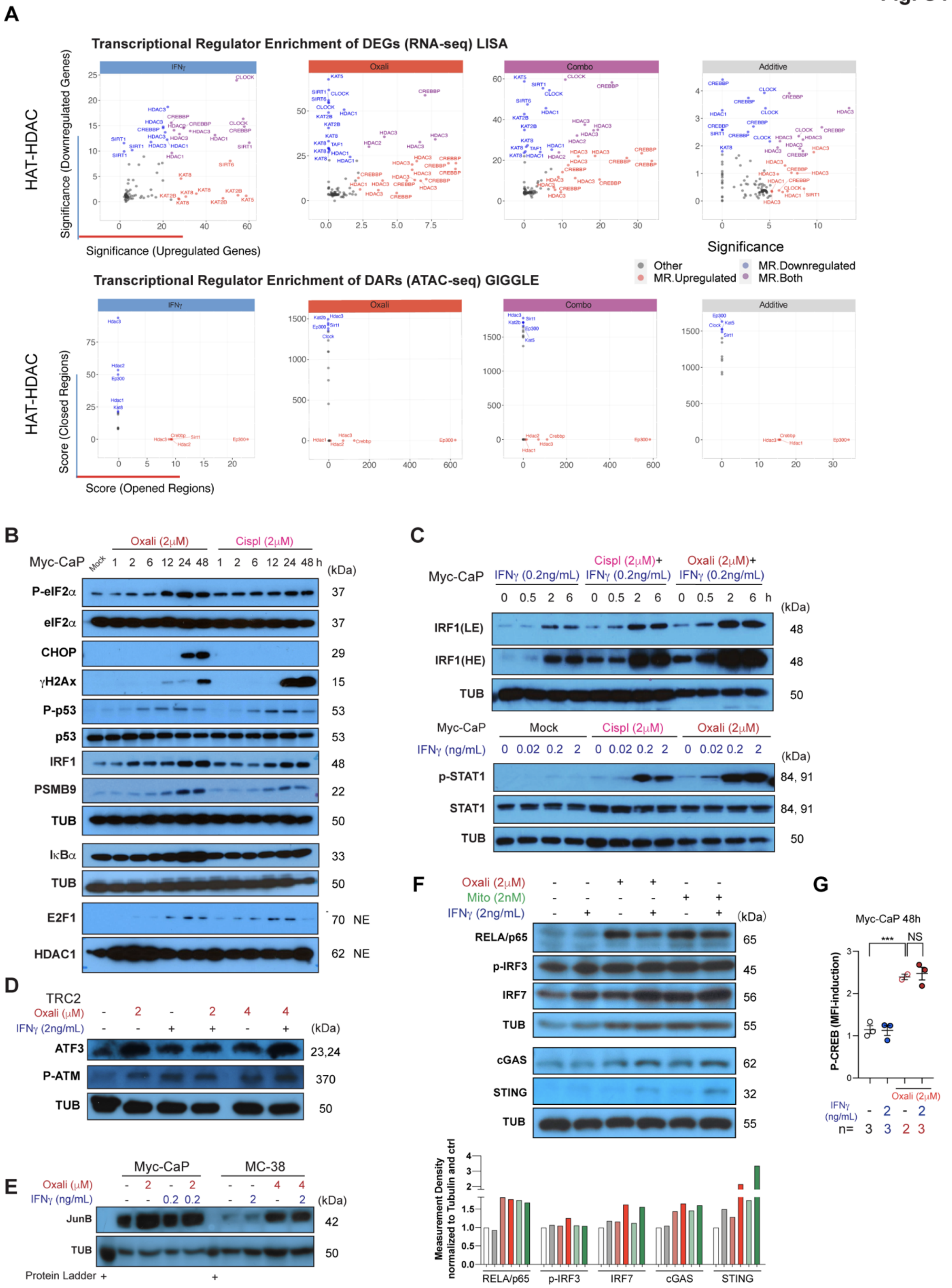
A
Transcriptional Regulator Enrichment of DEGs (RNA-seq) LISA
HAT-HDAC
Significance (Downregulated Genes)
Significance (Upregulated Genes)
IFNγ
Oxali
Combo
Additive
Significance
Other
MR.Upregulated
MR.Downregulated
MR.Both
Transcriptional Regulator Enrichment of DARs (ATAC-seq) GIGGLE
Score (Closed Regions)
Score (Opened Regions)
B
Myc-CaP
Mock
Oxali (2μM)
Cispl (2μM)
1 2 6 12 24 48 1 2 6 12 24 48 h
(kDa)
P-eIF2α 37
eIF2α 37
CHOP 29
γH2Ax 15
P-p53 53
p53 53
IRF1 48
PSMB9 22
TUB 50
IκBα 33
TUB 50
E2F1 70 NE
HDAC1 62 NE
C
Myc-CaP
IFNγ (0.2ng/mL)
Cispl (2μM)+ IFNγ (0.2ng/mL)
Oxali (2μM)+ IFNγ (0.2ng/mL)
0 0.5 2 6 0 0.5 2 6 0 0.5 2 6 h
(kDa)
IRF1(LE) 48
IRF1(HE) 48
TUB 50
Myc-CaP
Mock
Cispl (2μM)
Oxali (2μM)
IFNγ (ng/mL) 0 0.02 0.2 2 0 0.02 0.2 2 0 0.02 0.2 2
(kDa)
p-STAT1 84, 91
STAT1 84, 91
TUB 50
D
TRC2
Oxali (μM) - 2 - 2 4 4
IFNγ (2ng/mL) - - + + - +
(kDa)
ATF3 23,24
P-ATM 370
TUB 50
E
Myc-CaP
MC-38
Oxali (μM) - 2 - 2 - - 4 4
IFNγ (ng/mL) - - 0.2 0.2 - 2 - 2
(kDa)
JunB 42
TUB 50
Protein Ladder + +
F
Oxali (2μM) - - + + - -
Mito (2nM) - - - - + +
IFNγ (2ng/mL) - + - + - +
(kDa)
RELA/p65 65
p-IRF3 45
IRF7 56
TUB 55
cGAS 62
STING 32
TUB 55
Measurement Density normalized to Tubulin and ctrl
RELA/p65
p-IRF3
IRF7
cGAS
STING
G
Myc-CaP 48h
P-CREB (MFI-induction)
***
NS
Oxali (2μM)
IFNγ (ng/mL) - 2 - 2
n= 3 3 2 3

Fig. S4. Analysis of chemotherapy-induced histone modifiers and transcription factors. (A) LISA and GIGGLE were applied to DEGs and DARs identified in comparisons of IFNγ (2 ng/mL), Oxali (2 μM), and Combo treated cells relative to control, as well as to genes classified with additive response, as described in Fig 2I and 2J. The subset of regulators with HAT or HDAC activity are shown. The top enriched regulators of upregulated (red) and downregulated (blue) DEGs and DARs are noted. (B-E) Myc-CaP, TRAMP-C2 and MC-38 cells treated as indicated were IB analyzed for the indicated proteins. Both whole cell lysates (WL) and nuclear extracts (NE) were analyzed. HE and LE: high and low exposure, respectively. (F) Myc-CaP cells treated as indicated were lysed and IB analyzed for the indicated proteins using tubulin as a loading control (top). The induction of each protein upon different treatments were quantified using ImageJ (bottom). (G) Myc-CaP cells treated as indicated were analyzed for phosphorylated CREB1 by flow cytometry. Two-sided t-test (means ± s.e.m) was used to determine significance. *$P < 0.05$; **$P < 0.01$; ***$P < 0.001$; NS, not significant. Specific *n* values are shown in (G).

Fig. S5

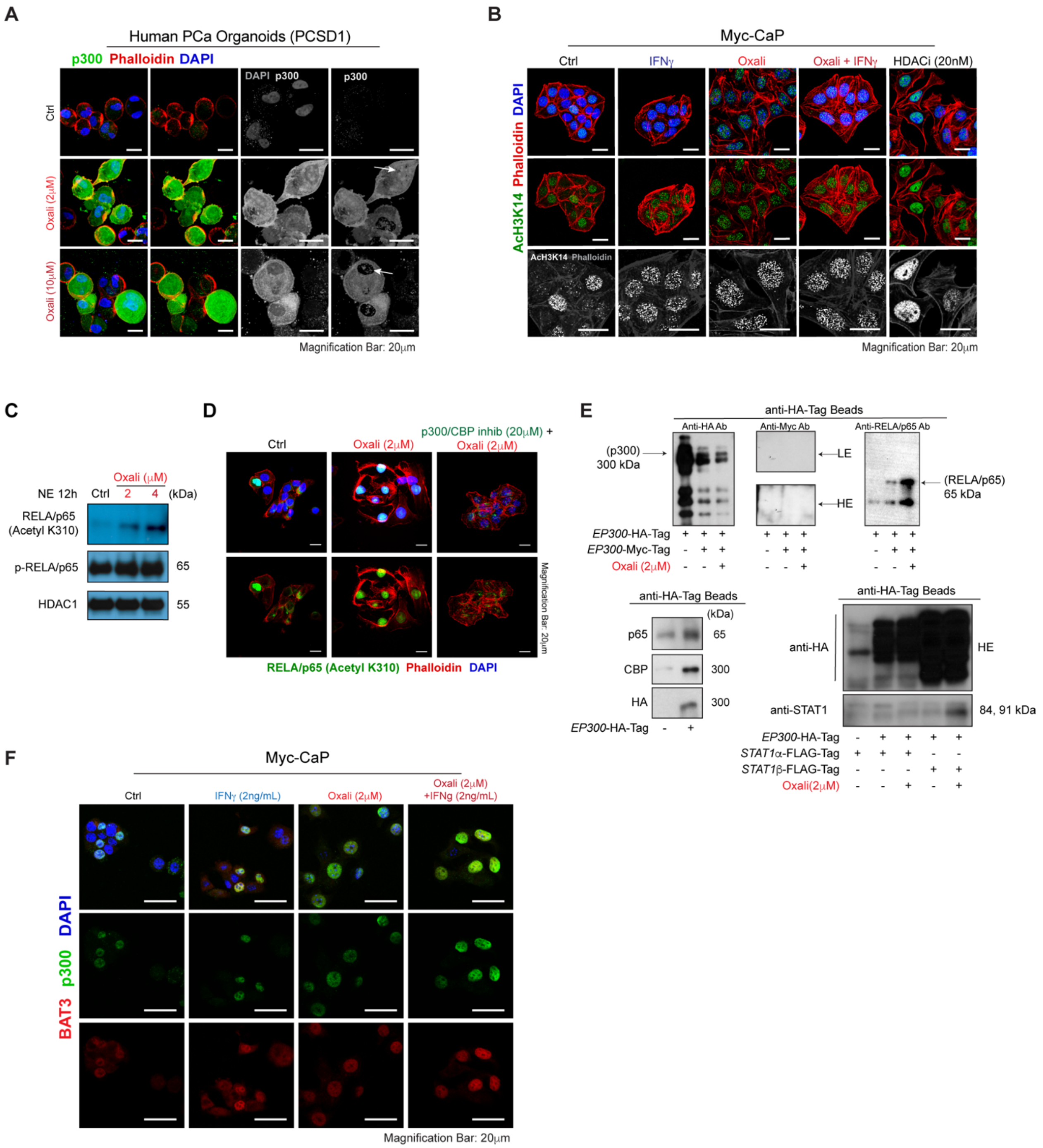

A
Human PCa Organoids (PCSD1)
p300 Phalloidin DAPI
DAPI p300
p300
Ctrl
Oxali (2μM)
Oxali (10μM)
Magnification Bar: 20μm
B
Myc-CaP
Ctrl
IFNγ
Oxali
Oxali + IFNγ
HDACi (20nM)
AcH3K14 Phalloidin DAPI
AcH3K14 Phalloidin
Magnification Bar: 20μm
C
NE 12h
Oxali (μM)
Ctrl 2 4 (kDa)
RELA/p65 (Acetyl K310)
p-RELA/p65 65
HDAC1 55
D
Ctrl
Oxali (2μM)
p300/CBP inhib (20μM) + Oxali (2μM)
Magnification Bar: 20μm
RELA/p65 (Acetyl K310) Phalloidin DAPI
E
anti-HA-Tag Beads
Anti-HA Ab
Anti-Myc Ab
Anti-RELA/p65 Ab
(p300) 300 kDa
LE
HE
(RELA/p65) 65 kDa
EP300-HA-Tag
EP300-Myc-Tag
Oxali (2μM)
anti-HA-Tag Beads
(kDa)
p65 65
CBP 300
HA 300
EP300-HA-Tag
anti-HA-Tag Beads
anti-HA
HE
anti-STAT1
84, 91 kDa
EP300-HA-Tag
STAT1α-FLAG-Tag
STAT1β-FLAG-Tag
Oxali(2μM)
F
Myc-CaP
Ctrl
IFNγ (2ng/mL)
Oxali (2μM)
Oxali (2μM) +IFNg (2ng/mL)
DAPI p300 BAT3
Magnification Bar: 20μm

Fig. S5. Chemotherapy stimulates HAT nuclear localization and activity. (A) PCSD1 organoids were treated with Oxali (2 or 10 μM) for 12 h and stained for p300 (green) and phalloidin (red; actin cables). Nuclei were counter stained with DAPI (blue) (n = 4). Arrows indicate nucleolar p300. Magnification bar: 20 μm. (B) Myc-CaP cells treated with low-dose Oxali, IFNγ, alone or together, and HDACi for 12 h were stained with antibodies against K14 acetylated H3 (green) and phalloidin (red). Nuclei were counterstained with DAPI (blue). Magnification bar: 20 μm. (C) Nuclear extracts of Myc-CaP cells treated as indicated were IB analyzed for acetylated p65/RelA (lysine 310), phosphorylated p65/RelA, and HDAC1 (loading control). (D) Myc-CaP cells treated as indicated were stained for acetylated p65/RelA (lysine K310) (green) and phalloidin (red). Nuclei were counterstained with DAPI (n = 2). Magnification bar: 20 μm. (E) 293T cells were transfected with indicated expression vectors coding for EP300 with a C-terminal Myc or N-terminal HA tags or STAT1 with a C-terminal Flag tag. After 24 h the cells were treated with Oxali for 24 h. EP300 protein was immunoprecipitated with an HA antibody and the disrupted immunocomplexes were IB analyzed with antibodies against the HA and Myc tags, p65/RelA, CBP and STAT1. LE and HE: low and high exposure, respectively. Arrows indicate the positions of expected bands. (F) Myc-CaP cells treated with Oxali and/or IFNγ for 24 h were stained with antibodies to p300 (green) and BAT3 (red) and DAPI (blue) (n = 3). Magnification bar: 20 μm.

Fig. S6

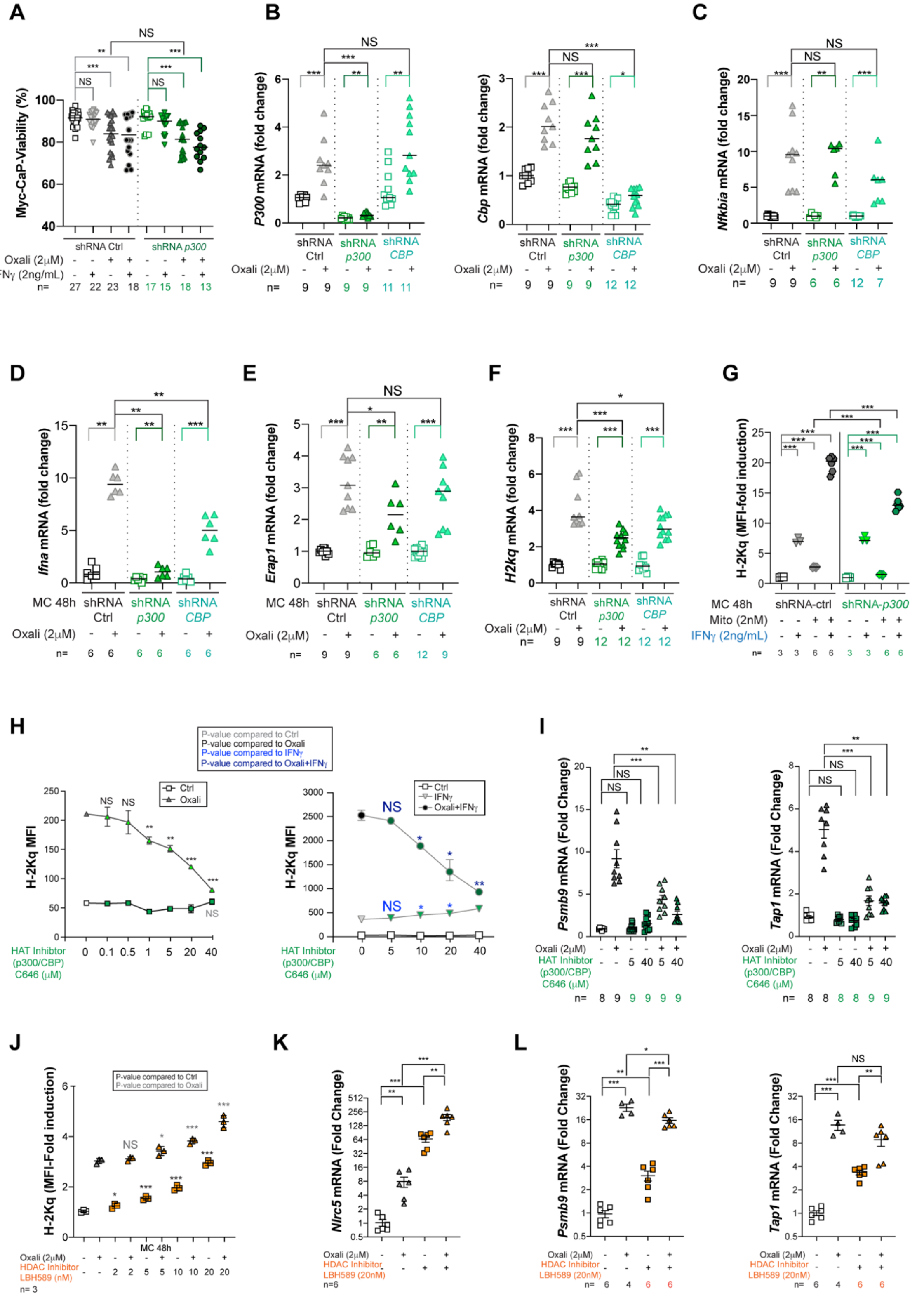

Fig. S6. p300 and CBP control Oxali and Mito-induced MHC-I AgPPM genes. (A) Control and *p300*-silenced Myc-CaP cells were stained with DAPI after each treatment and analyzed by flow cytometry to determine cell viability. (B) Control and *p300* or *CBP* silenced Myc-CaP cells were treated with Oxali and analyzed by qRT-PCR for *p300* and *C*bp mRNA expression to confirm successful silencing. (C-F) Myc-CaP cells with indicated gene edits were treated with Oxali and analyzed by qRT-PCR for *Nfkbia*, *Ifna*, *Erap1*, and *H2kq* mRNA expression. (G) Parental (shRNA-Ctrl) and *p300*-silenced Myc-CaP cells were incubated with Mito and/or IFNγ and analyzed for H-2Kq by flow cytometry. (H) Myc-CaP cells incubated with Oxali (2 μM), IFNγ (2 ng/mL), Oxali + IFNγ and different concentrations of the HAT inhibitor C646 were analyzed by flow cytometry for H-2Kq surface expression. (I) Myc-CaP cells treated as above were qRT-PCR analyzed for *Psmb9* and *Tap1* mRNA expression. (J-L) Myc-CaP cells treated with panobinostat (LBH589) as indicated for 48 h were analyzed by flow cytometry for H-2Kq (J) or by qRT-PCR for *Nlrc5* (K), *Psmb9* and *Tap1* (L) mRNA expression. Two-sided t-test (means ± s.e.m), and Mann–Whitney test (median) were used to determine significance. One-way ANOVA analysis and multiple comparison confirmed the results. *P < 0.05; **P < 0.01; ***P < 0.001; NS, not significant. Specific *n* values are shown in (A-G, I-L).

Fig. S7

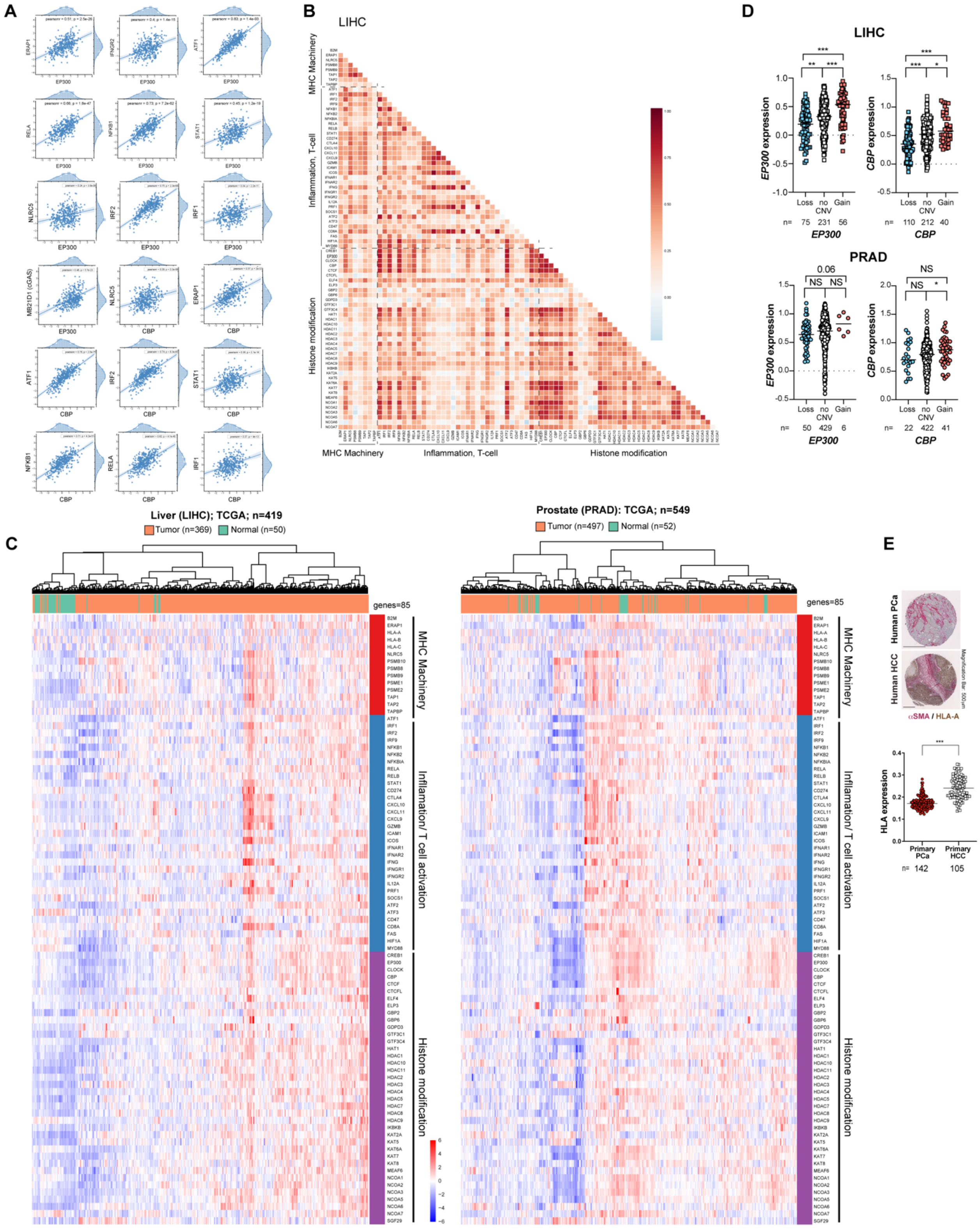
A
B
LIHC
MHC Machinery
Inflammation, T-cell
Histone modification
D
LIHC
EP300 expression
CBP expression
Loss
no CNV
Gain
n= 75 231 56
EP300
n= 110 212 40
CBP
PRAD
0.06
NS
*
n= 50 429 6
n= 22 422 41
Liver (LIHC); TCGA; n=419
Tumor (n=369)
Normal (n=50)
Prostate (PRAD): TCGA; n=549
Tumor (n=497)
Normal (n=52)
C
genes=85
MHC Machinery
Inflammation/ T cell activation
Histone modification
E
Human PCa
Human HCC
αSMA / HLA-A
HLA expression
Primary PCa
Primary HCC
n= 142 105

Fig. S7. p300/CBP control MHC-I AgPPM expression in human cancers. (A) Pearson correlations of gene expression in LIHC (n = 369). Pearson correlation coefficients and corresponding p-values show the correlation of *EP300* or *CBP* expression with expression of *ERAP1, IFNGR2, NLRC5, IRF1, IRF2, ATF1, RELA, STAT1*, *MB211D (cGAS),* or *NFKB1*. Linear lines (blue) and confidence intervals (light blue shade) depict the results of the linear regression analyses. Histograms for each gene are shown on the side (top and right bars). (B) Pairwise Pearson correlations of MHC genes, inflammation/T-cell genes, and histone modification genes in the LIHC data set. Dashed lines separate the three gene subgroups. (C) Expression of genes involved in immune response, antigen presentation, and histone modification in non-tumor and tumor samples of LIHC (left) or PRAD (right) was analyzed through un-supervised mining of the TCGA data base. (D) Correction of CNV status and gene expression for *EP300* and *CBP* in LIHC (n = 362) and PRAD (n = 485) TCGA data sets. (E) Tumor microarrays encompassing 142 primary PCa and 105 HCC patients (5-6 spots per patient = 3-4 tumor and 2 non-tumor) were stained for HLA-A (brown) and αSMA (red). Nuclei were counterstained with haematoxylin. Representative samples (top) and quantification performed by ImageJ (bottom) are shown. Each dot represents one patient. Magnification bar: 500 μm. Mann–Whitney test (median) was used to determine significance (D, E). *$P < 0.05$; **$P < 0.01$; ***$P < 0.001$; NS, not significant. Specific *n* values are shown in (D-E).

Fig. S8

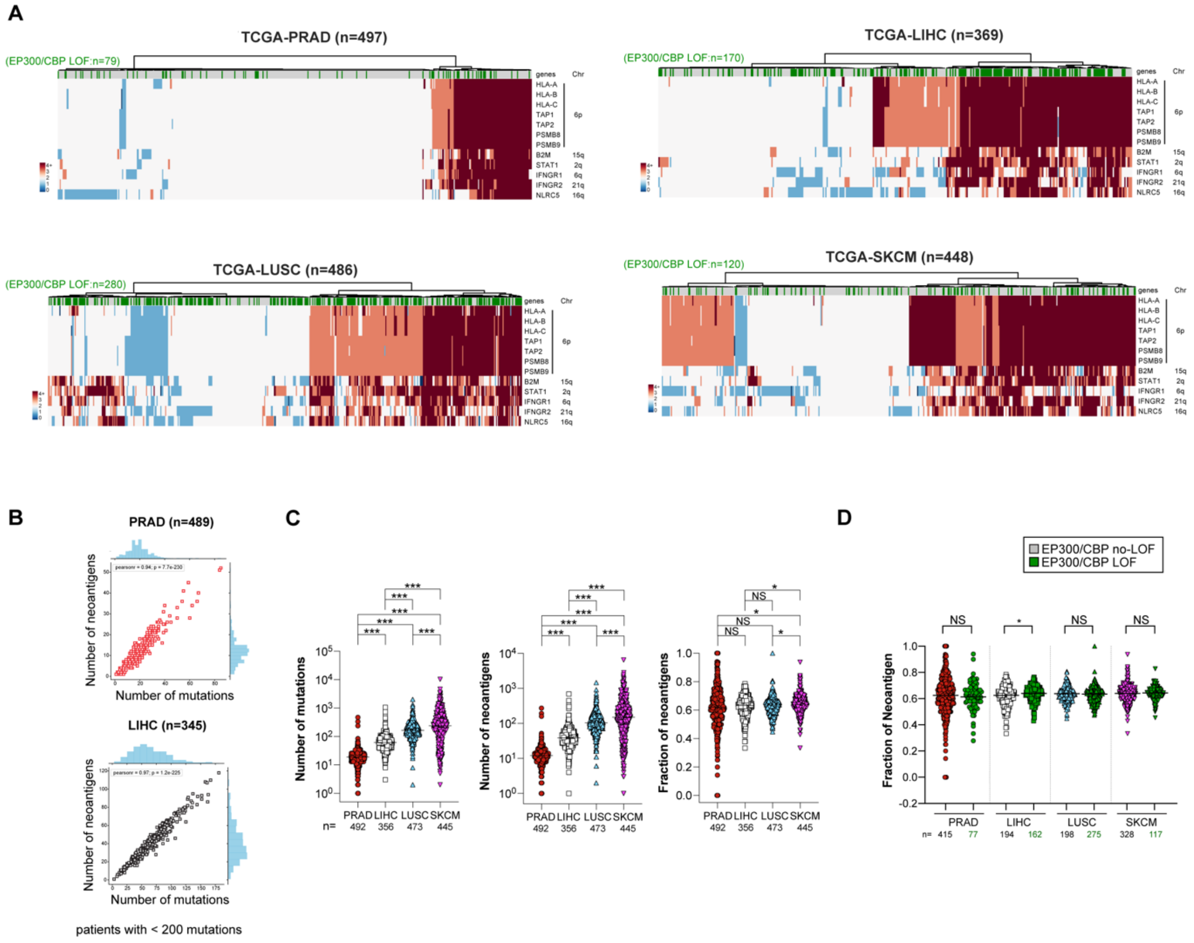

Fig. S8. p300/CBP control MHC-I AgPPM genes and neoantigen generation/expression in human cancers. (A) CNV losses and gains of AgPPM genes (*HLA-A/B/C*, *TAP1/2*, *PSMB8/9*, *B2M*, *STAT1*, *IFNGR1/2*, *NLRC5*) in PRAD (n = 497), LIHC (n = 369), LUSC (n = 486) and SKCM (n = 448) are shown as indicated. CNV key: dark blue (homozygous deletion), light blue (one copy loss), white (no CNV), pink (one copy gain), and red (high amplification). Each green line on the top bar represents a patient with *EP300/CBP* LOF. (B) Pearson correlations of number of neoantigens (y-axis) and number of total mutations (x-axis) in the LIHC and PRAD data sets. Outliers with high mutation burdens were excluded and only tumors with less than 200 mutations were considered Linear smoothed line (blue lines) and confidence intervals (light blue shade) are shown based on linear regression analyses. Histograms representing each gene expression are shown on the sides (top and right bars). (C) Neoantigen landscape in four tumor types: PRAD (n = 492), LIHC (n = 356), LUSC (n = 473), and SKCM (n = 445). Neoantigens were determined based on peptide binding affinity predicted by NetMHCPan 4.0 analysis of TCGA mRNA-seq reads. P-values are based on Wilcoxon rank-sum test. (D) Fraction of neoantigens among the above tumor types divided into *EP300/CBP* LOF (green dots) and non-LOF groups. P-values are based on Wilcoxon rank-sum test. Mann–Whitney test (median) was used to determine significance (C, D). *P < 0.05; **P < 0.01; ***P < 0.001; NS, not significant. Specific *n* values are shown in (A-D).

Fig. S9

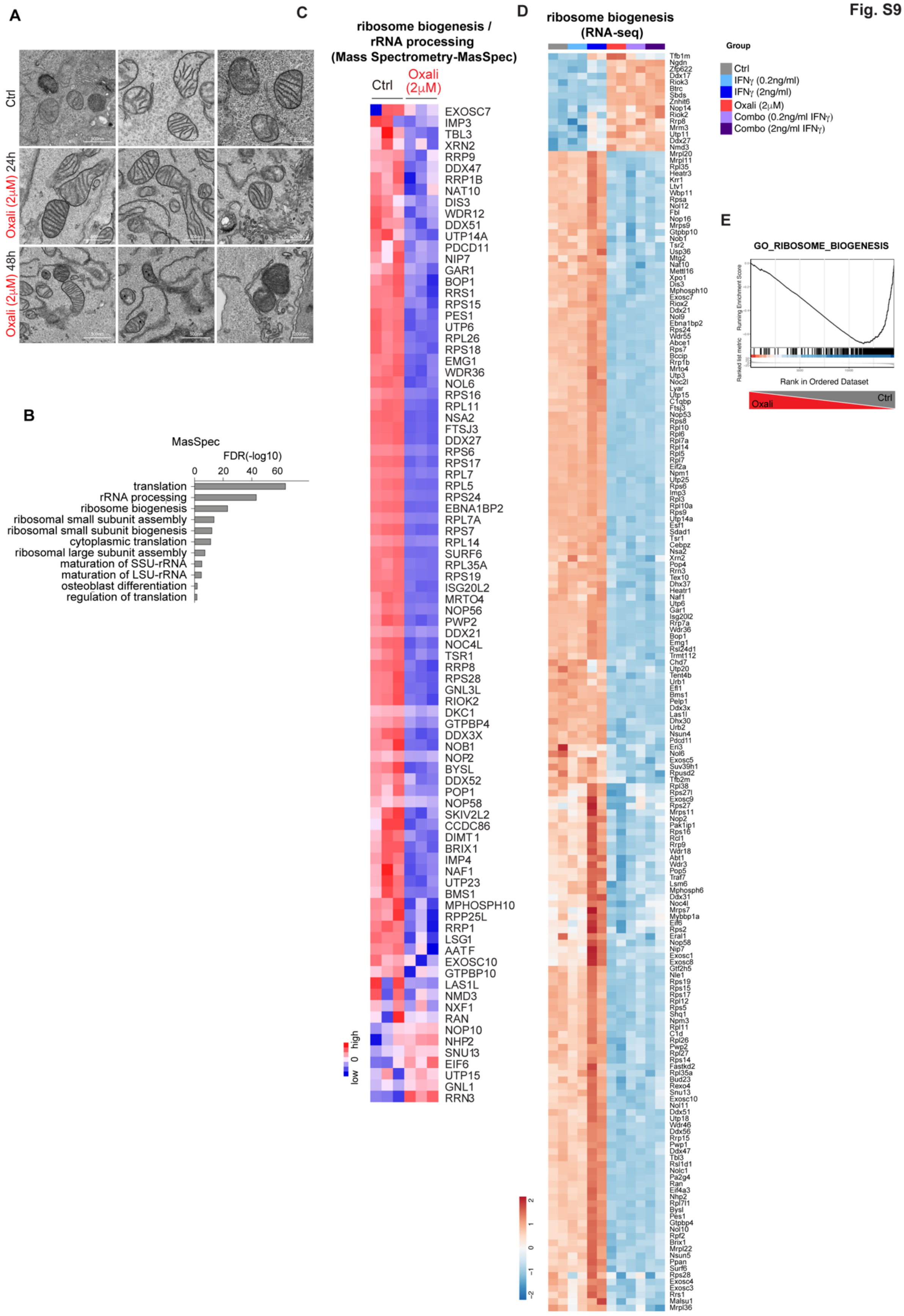
A
Ctrl
Oxali (2μM) 24h
Oxali (2μM) 48h
B
MasSpec
FDR(-log10)
0
20
40
60
translation
rRNA processing
ribosome biogenesis
ribosomal small subunit assembly
ribosomal small subunit biogenesis
cytoplasmic translation
ribosomal large subunit assembly
maturation of SSU-rRNA
maturation of LSU-rRNA
osteoblast differentiation
regulation of translation
C
ribosome biogenesis / rRNA processing (Mass Spectrometry-MasSpec)
Ctrl
Oxali (2μM)
EXOSC7
IMP3
TBL3
XRN2
RRP9
DDX47
RRP1B
NAT10
DIS3
WDR12
DDX51
UTP14A
PDCD11
NIP7
GAR1
BOP1
RRS1
RPS15
PES1
UTP6
RPL26
RPS18
EMG1
WDR36
NOL6
RPS16
RPL11
NSA2
FTSJ3
DDX27
RPS6
RPS17
RPL7
RPL5
RPS24
EBNA1BP2
RPL7A
RPS7
RPL14
SURF6
RPL35A
RPS19
ISG20L2
MRTO4
NOP56
PWP2
DDX21
NOC4L
TSR1
RRP8
RPS28
GNL3L
RIOK2
DKC1
GTPBP4
DDX3X
NOB1
NOP2
BYSL
DDX52
POP1
NOP58
SKIV2L2
CCDC86
DIMT1
BRIX1
IMP4
NAF1
UTP23
BMS1
MPHOSPH10
RPP25L
RRP1
LSG1
AATF
EXOSC10
GTPBP10
LAS1L
NMD3
NXF1
RAN
NOP10
NHP2
SNU13
EIF6
UTP15
GNL1
RRN3
low 0 high
D
ribosome biogenesis (RNA-seq)
Group
Ctrl
IFNγ (0.2ng/ml)
IFNγ (2ng/ml)
Oxali (2μM)
Combo (0.2ng/ml IFNγ)
Combo (2ng/ml IFNγ)
2
1
0
−1
−2
E
GO_RIBOSOME_BIOGENESIS
Running Enrichment Score
Ranked list metric
Rank in Ordered Dataset
Oxali
Ctrl

Fig. S9. Oxali induces nucleolar/ribosomal stress. (A) Myc-CaP cells treated as indicated were fixed and examined by electron microscopy. Magnification bars are indicated in each image. (B-C) Myc-CaP cells were subjected to mass spectrometry analysis before and after 24 h Oxali (2 μM) treatment (n = 3). Top 10 hallmark gene sets were identified by gene set enrichment analysis (B). The relative expression values are presented by a heatmap (C). (D-E) GSEA was applied to RNA-seq data of Myc-CaP cells before and after Oxali treatment. Candidate enrichment plots for pathways related to ribosomal biogenesis are shown in (E) with a heatmap depicting the corresponding genes in (D).

Fig. S10

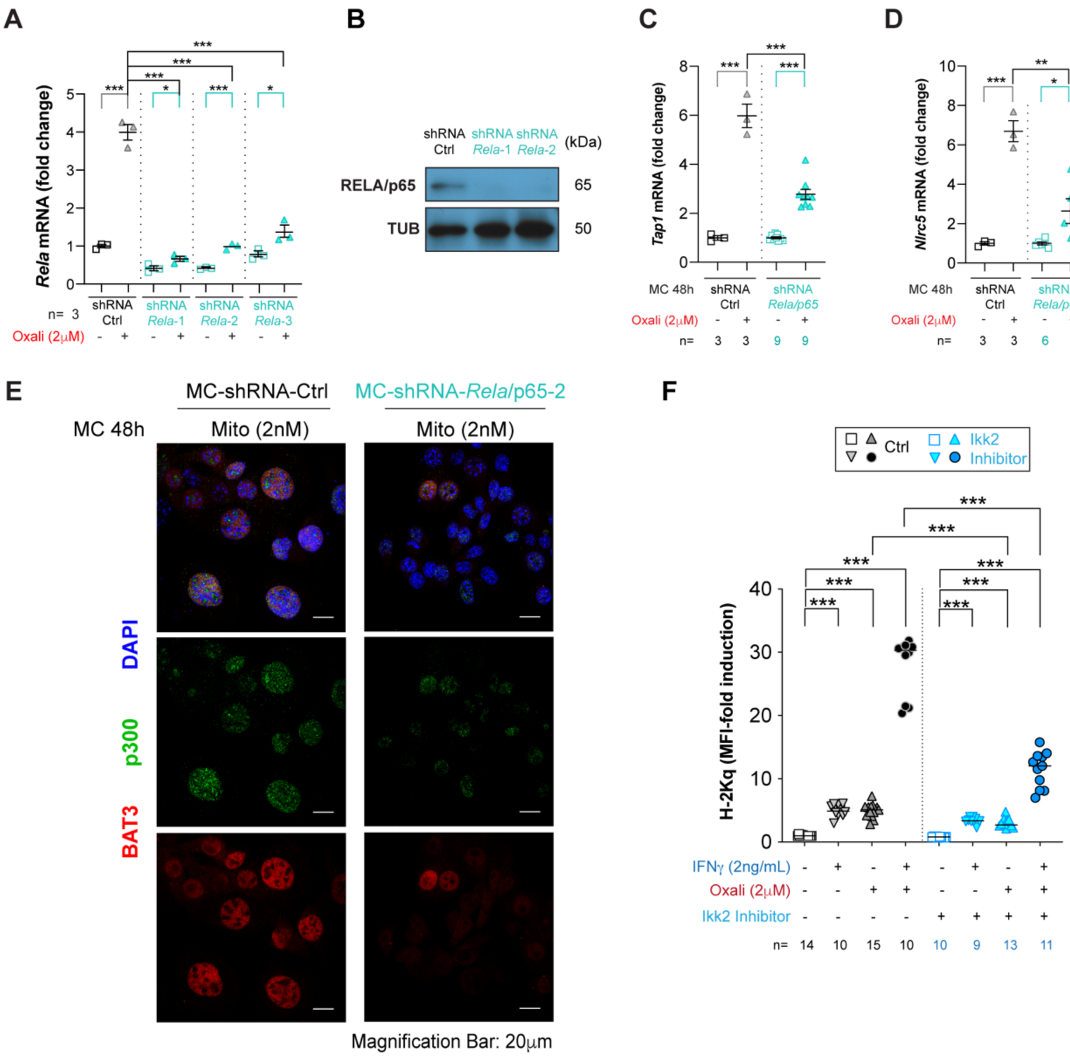

Fig. S10. NF-κB signaling and MHC-I AgPPM induction. (A-B) Control or *Rela/p65* silenced Myc-CaP cells were treated with Oxali for 48 h and analyzed by qRT-PCR (A), and IB (B) to confirm knockdown. Different *Rela/p65* shRNAs were used. Tubulin was used as a loading control. (C-D) Parental (shRNA-Ctrl) or *Rela/p65*-silenced Myc-CaP cells were incubated with Oxali as indicated. RNAs were analyzed by qRT-PCR with the indicated primers. (E) Parental or *Rela/p65*-silenced Myc-CaP cells were treated with Mito and stained with mouse-anti p300 (green) and rabbit-anti BAT3 (red). Nuclei were counterstained with DAPI (blue) (n = 3). Magnification bar: 20 μm. (F) Myc-CaP cells treated as indicated with the IKKβ inhibitors ML120B and IV and analyzed by flow cytometry for H-2Kq surface expression. Two-sided t-test (means ± s.e.m), and Mann–Whitney test (median) were used to determine significance. One-way ANOVA analysis and multiple comparison confirmed the results. *P < 0.05; **P < 0.01; ***P < 0.001; NS, not significant. Specific *n* values are shown in (A, C, D, F).

Fig. S11

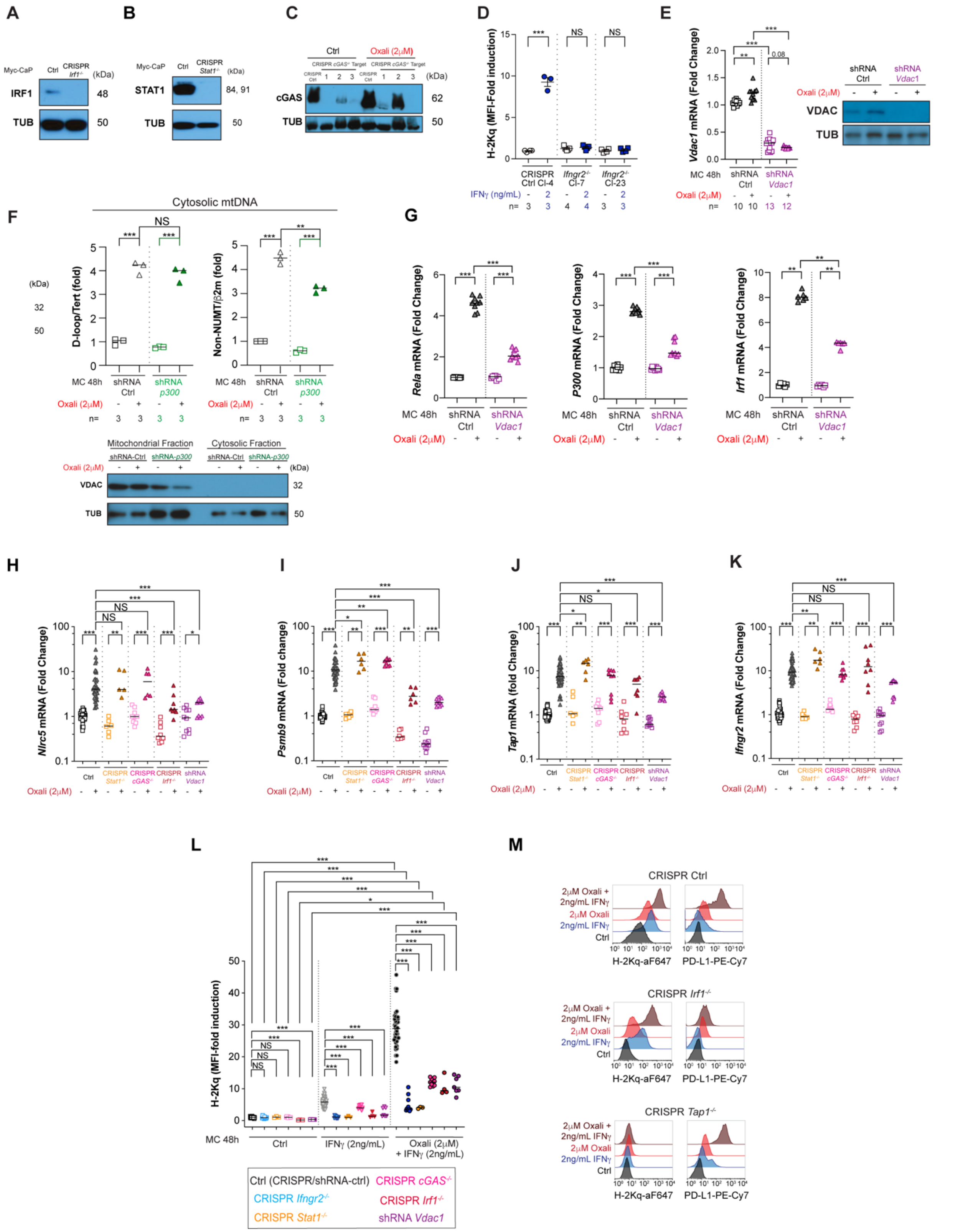
A
B
C
D
E
F
G
H
I
J
K
L
M
Myc-CaP
Ctrl
CRISPR Irf1-/-
(kDa)
IRF1
TUB
48
50
CRISPR Stat1-/-
STAT1
84, 91
Oxali (2μM)
CRISPR cGAS-/- Target
cGAS
62
H-2Kq (MFI-Fold induction)
CRISPR Ctrl Cl-4
Ifngr2-/- Cl-7
Ifngr2-/- Cl-23
IFNγ (ng/mL)
NS
Vdac1 mRNA (Fold Change)
MC 48h
shRNA Ctrl
shRNA Vdac1
VDAC
Cytosolic mtDNA
D-loop/Tert (fold)
Non-NUMT/β2m (fold)
shRNA p300
Mitochondrial Fraction
Cytosolic Fraction
shRNA-Ctrl
shRNA-p300
32
Rela mRNA (Fold Change)
P300 mRNA (Fold Change)
Irf1 mRNA (Fold Change)
Nlrc5 mRNA (Fold Change)
Psmb9 mRNA (Fold change)
Tap1 mRNA (fold Change)
Ifngr2 mRNA (Fold Change)
CRISPR Stat1-/-
CRISPR cGAS-/-
CRISPR Irf1-/-
shRNA Vdac1
H-2Kq (MFI-fold induction)
IFNγ (2ng/mL)
Oxali (2μM) + IFNγ (2ng/mL)
Ctrl (CRISPR/shRNA-ctrl)
CRISPR Ifngr2-/-
CRISPR Ctrl
CRISPR Tap1-/-
2μM Oxali + 2ng/mL IFNγ
2μM Oxali
2ng/mL IFNγ
H-2Kq-aF647
PD-L1-PE-Cy7

Fig. S11. Mitochondrial (mt)DNA, VDAC1 and MHC-I AgPPM induction. (A-C) *Irf1*, cGAS and *Stat1* ablated Myc-CaP cells were treated as indicated and IB analyzed for IRF1 (A), STAT1 (B), or cGAS (C) to confirm successful gene editing. (D) Control and *Ifngr2* ablated Myc-CaP cells (edited by transient CRISPR-Cas9) were treated with IFNγ and analyzed by flow cytometry for surface H-2Kq expression to confirm IFNγ non-responsiveness. (E) Control or *Vdac1* silenced Myc-CaP cells were treated with Oxali (2 μM) for 48 h and analyzed by qRT-PCR (left) and IB (right) to confirm knockdown. (F) Cytosolic mtDNA amounts in Oxali-treated control and *p300*-silenced Myc-CaP cells were determined by qRT-PCR (top). Mitochondrial and cytosolic fractions of the same cells were IB analyzed with a VDAC antibody to confirm no mitochondrial contamination of the cytosolic fractions (bottom). (G) RNAs from control and *Vdac1*-silenced Myc-CaP cells treated with Oxali (2 μM) for 48 h were analyzed by qRT-PCR for expression of indicated mRNAs. (H-K) Gene edited Myc-CaP cells were treated with or without Oxali and analyzed by qRT-PCR for *Nlrc5*, *Psmb9*, *Tap1* and *Ifngr2* mRNA expression. (L) Gene edited Myc-CaP cells were treated as indicated and analyzed for surface MHC-I (H-2Kq) expression. (M) Control and gene edited Myc-CaP cells were treated as indicated and analyzed by flow cytometry for surface H-2Kq and PD-L1 expression. Two-sided t-test (means ± s.e.m), and Mann–Whitney test (median) were used to determine significance. One-way ANOVA analysis and multiple comparison confirmed the results. *P < 0.05; **P < 0.01; ***P < 0.001; NS, not significant. Specific *n* values are shown in (D-F).

Fig. S12

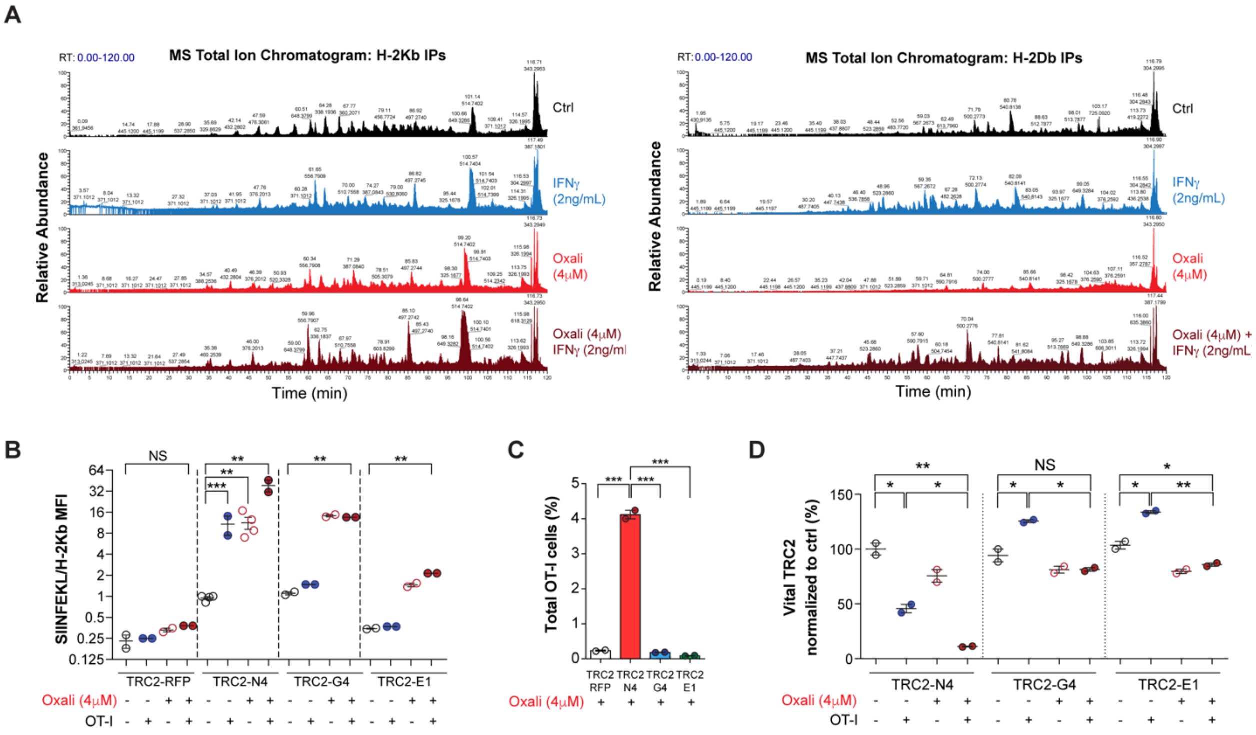

Fig. S12. Oxali induced functional antigen presentation. (A) MC-38 colon carcinoma cells were treated with Oxali and/or IFNγ for 48 h and 6.7E+08 cells were lysed per each condition and subjected to IP with H-2Kb or H-2Db antibodies. MHC-I bound peptides were extracted and analyzed by mass spectrometry. (B-D) TRC2 cells transfected with vectors expressing high, medium, and low affinity ovalbumin variants were incubated with 4 μM Oxali and/or CFSE-labeled OT-I cells for 72 h and analyzed by flow cytometry using an antibody against H-2Kb bound SIINFEKL (B). The number of OT-I cells in each culture (C) and the percentages of viable TRC2 cells (D) were determined by flow cytometry. Two-sided t-test (means ± s.e.m), and Mann–Whitney test (median) were used to determine significance. *P < 0.05; **P < 0.01; ***P < 0.001; NS, not significant.

Fig. S13

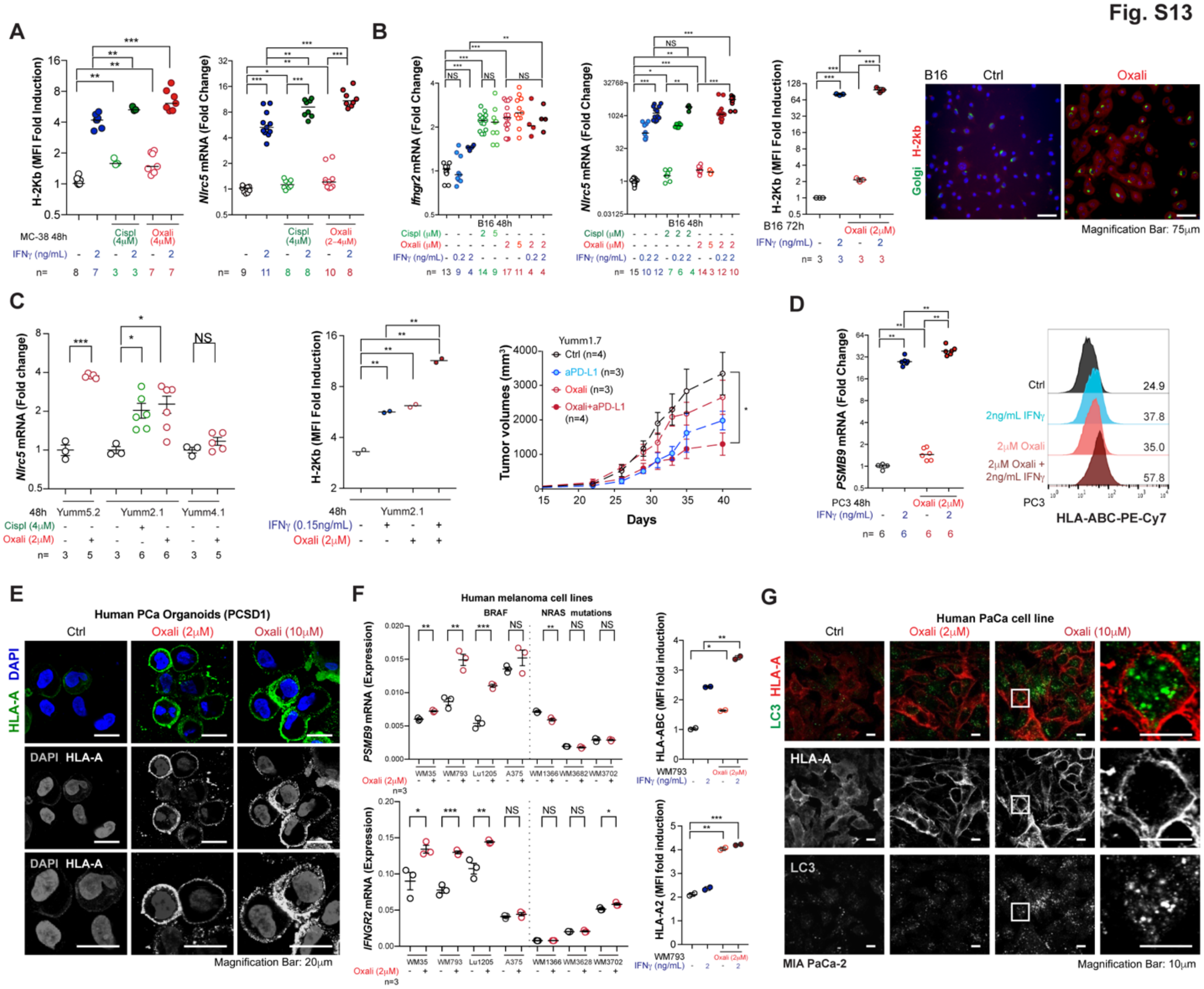

A
B
C
D
E
F
G
Human PCa Organoids (PCSD1)
Human melanoma cell lines
Human PaCa cell line
Magnification Bar: 75μm
Magnification Bar: 20μm
Magnification Bar: 10μm
MIA PaCa-2
HLA-ABC-PE-Cy7

Fig. S13. Chemotherapy induces MHC-I AgPPM genes in murine and human cancers. (A) MC-38 cells were treated as indicated and analyzed by flow cytometry for surface H-2Kb expression and by qRT-PCR for *Nlrc5* mRNA expression. (B) B16 melanoma cells were treated as indicated and analyzed by qRT-PCR for *Ifngr2* and *Nlrc5* mRNA expression, flow cytometry for H-2Kb surface expression, and IF for intracellular H-2Kb (red), Golgi apparatus (green) and DAPI (blue) (n = 3). Magnification bar: 75 μm. (C) Mouse melanoma Yumm2.1, Yumm4.1 and Yumm5.2 cell lines were treated as indicated and analyzed by qRT-PCR for *Nlrc5* mRNA expression (left). Surface H-2Kb expression on Yumm2.1 cells was analyzed by flow cytometry (middle). *C57BL/6* mice bearing s.c. Yumm1.7 tumors were treated as indicated. After three treatment cycles, mice were euthanized and analyzed (right). Significance was determined by Two-sided t-test. Each dot represents a treatment group mean ± s.e.m (n = 3-4). (D) Human PCa PC3 cells were treated as indicated and analyzed by qRT-PCR for *PSMB9* mRNA expression (left), or by flow cytometry for surface HLA-ABC expression (right). (E) PCSD1 organoids were treated with Oxali (2 and 10 μM) as indicated for 12 h and stained for HLA-A (green). Nuclei were counter stained with DAPI (blue) (n = 2-3). Magnification bar: 20 μm. (F) Human melanoma cell lines bearing *BRAF* (V600E) or *NRAS* mutations were treated with Oxali and analyzed by qRT-PCR for mRNA expression of *PSMB9* and *IFNGR2*. Human WM793 melanoma cells were treated as indicated and analyzed for surface MHC expression (HLA-ABC and HLA-A2) by flow cytometry. (G) Human MIA PaCa-2 cells were incubated with Oxali (2 and 10 μM) for 24 h and stained with HLA-A (red) and LC3 (green) antibodies and counterstained with DAPI. Stained cells were examined by indirect immunofluorescence. Magnification bar: 10 μm. Two-sided t-test (means ± s.e.m), and Mann–Whitney test (median) were used to determine significance. $^{*}P < 0.05$; $^{**}P < 0.01$; $^{***}P < 0.001$; NS, not significant. Specific *n* values are shown in (A-D, F).

Fig. S14

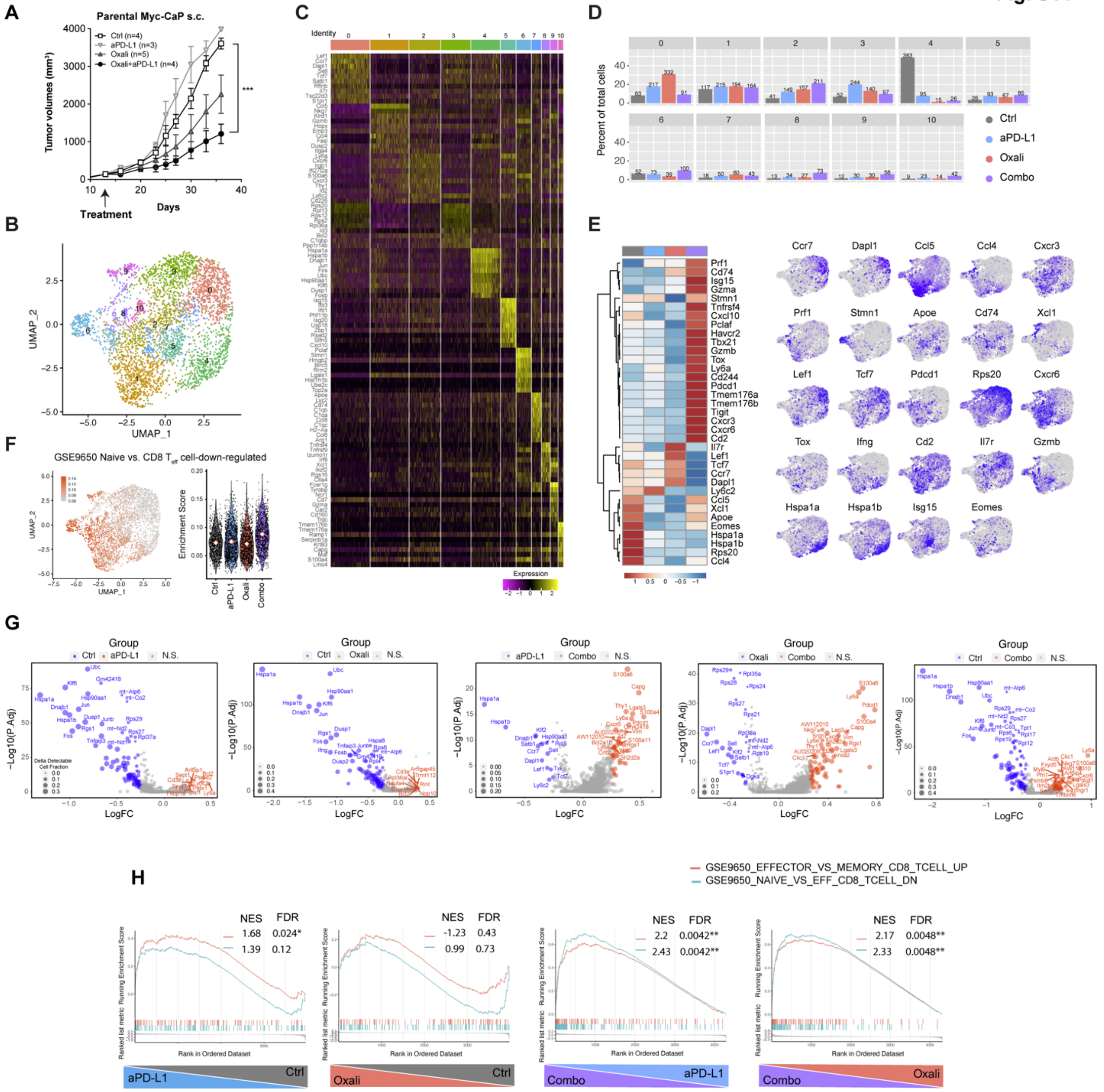

A
Parental Myc-CaP s.c.
Ctrl (n=4)
aPD-L1 (n=3)
Oxali (n=5)
Oxali+aPD-L1 (n=4)
Tumor volumes (mm³)
Days
Treatment
***
B
UMAP_1
UMAP_2
C
Identity
Expression
D
Percent of total cells
Ctrl
aPD-L1
Oxali
Combo
E
Prf1
Cd74
Isg15
Gzma
Stmn1
Tnfrsf4
Cxcl10
Pclaf
Havcr2
Tbx21
Gzmb
Tox
Ly6a
Cd244
Pdcd1
Tmem176a
Tmem176b
Tigit
Cxcr3
Cxcr6
Cd2
Il7r
Lef1
Tcf7
Ccr7
Dapl1
Ly6c2
Ccl5
Xcl1
Apoe
Eomes
Hspa1a
Hspa1b
Rps20
Ccl4
F
GSE9650 Naive vs. CD8 T_{eff} cell-down-regulated
Enrichment Score
Ctrl
aPD-L1
Oxali
Combo
G
Group
LogFC
−Log10(P.Adj)
N.S.
H
GSE9650_EFFECTOR_VS_MEMORY_CD8_TCELL_UP
GSE9650_NAIVE_VS_EFF_CD8_TCELL_DN
NES FDR
1.68 0.024*
1.39 0.12
-1.23 0.43
0.99 0.73
2.2 0.0042**
2.43 0.0042**
2.17 0.0048**
2.33 0.0048**
Running Enrichment Score
Ranked list metric
Rank in Ordered Dataset
aPD-L1
Ctrl
Oxali
Ctrl
Combo
aPD-L1
Combo
Oxali

Fig. S14. Combination chemo-immunotherapy increases Teff function. (A) *FVB/N* mice bearing s.c. Myc-CaP tumors were allocated into 4 treatment groups: (1) control (5% dextrose), (2) Oxali (6 mg/kg; weekly), (3) anti-PD-L1 (10 mg/kg; weekly), and (4) Oxali + anti-PD-L1 (weekly). After 4 cycles, during which tumor size was measured with a caliper, the mice were euthanized and analyzed. Each dot represents a treatment group average ± s.e.m. Two-sided t-test (means ± s.e.m), and two-way ANOVA were used to determine significance. (B) UMAP representation of total T cell populations profiled by scRNA-seq. Eleven distinct clusters were identified. (C) Heatmap representing the 10 most significant marker genes for each cluster. (D) Proportional contributions of each individual cluster to the overall T cell population in each sample is shown (Fig. 7B). Total cell numbers in each cluster are noted. (E) Heatmap depicting scaled mean expression of candidate genes across samples (left). Relative expression of candidate genes across all T cells (right; blue = maximum expression, gray = minimum expression). (F) Single cell pathway enrichment was applied for candidate pathways defining Tnaïve signatures. Enrichment scores are plotted across all cells (left) and distributions within samples (right). (G) Volcano plots depicting differential gene expression between T cell populations from each sample. Significant DEGs for each respective group are shown (red, blue). Top 20 DEGs associated with each sample are indicated. (H) Enrichment plots for candidate pathways defining $CD8^+$ Teff over Tmem and Tnaïve cells. Enrichment plots for each respective comparison are shown. $^*P < 0.05$; $^{**}P < 0.01$; $^{***}P < 0.001$; NS, not significant. Specific *n* values are shown in (A).

Fig. S15

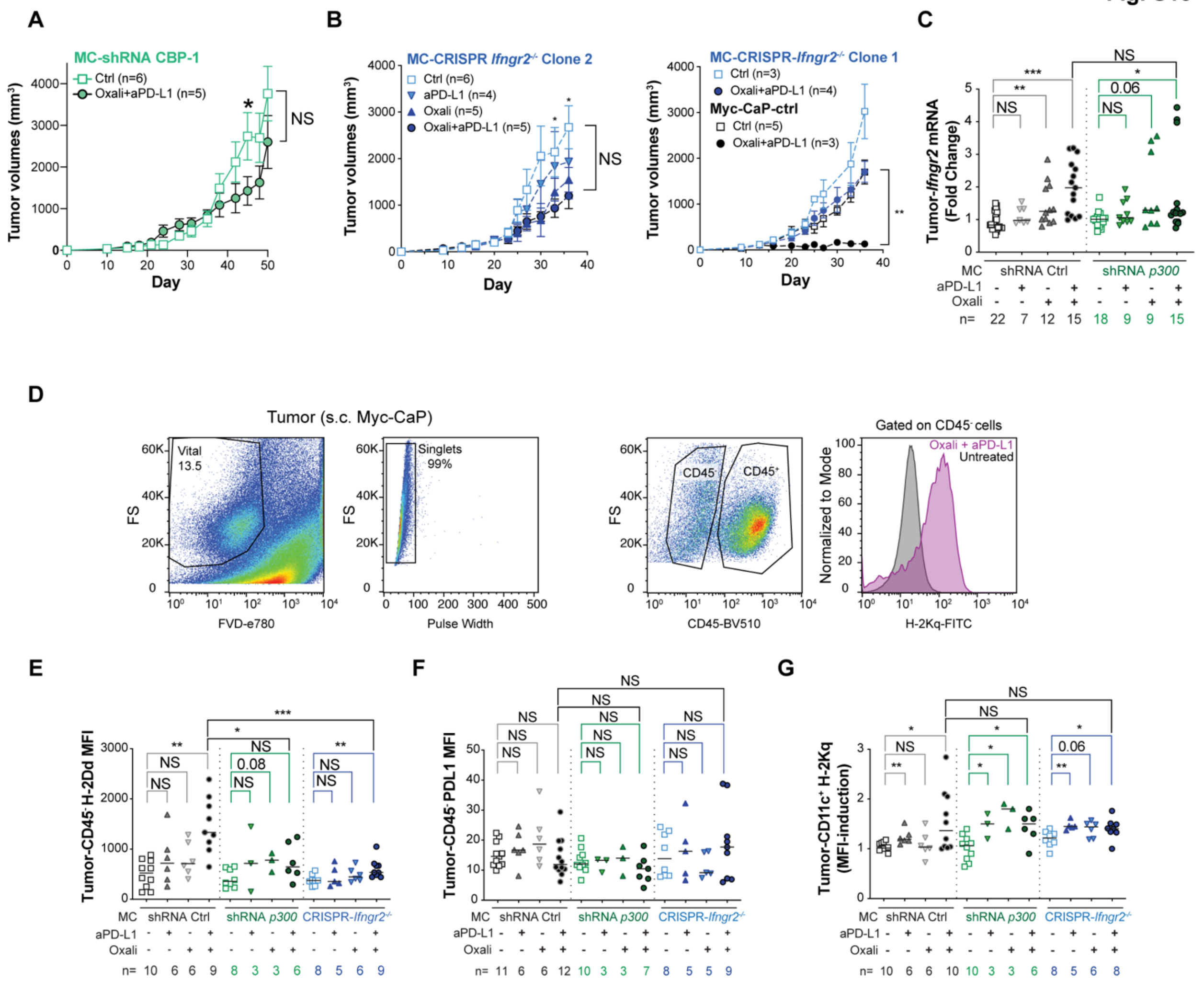
A
MC-shRNA CBP-1
Ctrl (n=6)
Oxali+aPD-L1 (n=5)
Tumor volumes (mm³)
Day
NS
B
MC-CRISPR Ifngr2-/- Clone 2
Ctrl (n=6)
aPD-L1 (n=4)
Oxali (n=5)
Oxali+aPD-L1 (n=5)
MC-CRISPR-Ifngr2-/- Clone 1
Ctrl (n=3)
Oxali+aPD-L1 (n=4)
Myc-CaP-ctrl
Ctrl (n=5)
Oxali+aPD-L1 (n=3)
C
Tumor-Ifngr2 mRNA (Fold Change)
MC shRNA Ctrl shRNA p300
aPD-L1
Oxali
n= 22 7 12 15 18 9 9 15
D
Tumor (s.c. Myc-CaP)
Vital 13.5
Singlets 99%
FVD-e780
Pulse Width
CD45-BV510
Gated on CD45- cells
Oxali + aPD-L1
Untreated
Normalized to Mode
H-2Kq-FITC
E
Tumor-CD45- H-2Dd MFI
F
Tumor-CD45- PDL1 MFI
G
Tumor-CD11c+ H-2Kq (MFI-induction)
MC shRNA Ctrl shRNA p300 CRISPR-Ifngr2-/-
n= 10 6 6 9 8 3 3 6 8 5 6 9
n= 11 6 6 12 10 3 3 7 8 5 5 9
n= 10 6 6 10 10 3 3 6 8 5 6 8

Fig. S15. Oxali + anti-PD-L1 synergy depends on p300/CBP and IFNγR2 expression. (A) Mice bearing s.c. tumors generated by *CBP*-silenced Myc-CaP cells were allocated into 2 treatment groups: (1) control (n=6), (2) Oxali + anti-PD-L1 (weekly) (n = 5) and analyzed as described in (A). Each dot represents a treatment group, mean ± s.e.m. Two-sided t-test (means ± s.e.m), and two-way ANOVA were used to determine significance. (B) Mice bearing s.c. tumors generated by control and *Ifngr2*-ablated (clone 1 and 2) Myc-CaP cells were treated as indicated. Treatment cycles and tumor measurements were performed as described in Fig. 7A. Each dot represents a treatment group mean ± s.e.m. Two-sided t-test (means ± s.e.m), and two-way ANOVA were used to determine significance. (C) Mice bearing s.c. Myc-CaP tumors generated by control and *p300*-silenced cells as described in Fig 7A. Total tumor RNA isolated from indicated treatment groups was analyzed by qRT-PCR for expression of the *Ifngr2*. (D) Single splenocyte or Myc-CaP tumor cell suspensions were stained for CD45, H-2Kq, H2-Dd, CD11b. The gating strategies are shown: all/lymphocyte gate, dead cell exclusion, doublet exclusion, and $CD45^+$ population gate. A histogram comparing the H-2Kq MFI of $CD45^-$ cancer cells isolated from Oxali + anti-PD-L1 treated and untreated tumor-bearing mice is shown. The H-2Kq analysis is shown in Fig. 7L. (E-G) Single cell tumor suspensions were analyzed by flow cytometry for H-2Dd (E) and PD-L1 (F) expression on $CD45^-$ cells and H-2Kq expression on $CD11c^+$ cells (G). $^*P < 0.05$; $^{**}P < 0.01$; $^{***}P < 0.001$; NS, not significant. Specific *n* values are shown in (A-C, E-G).

Fig. S16

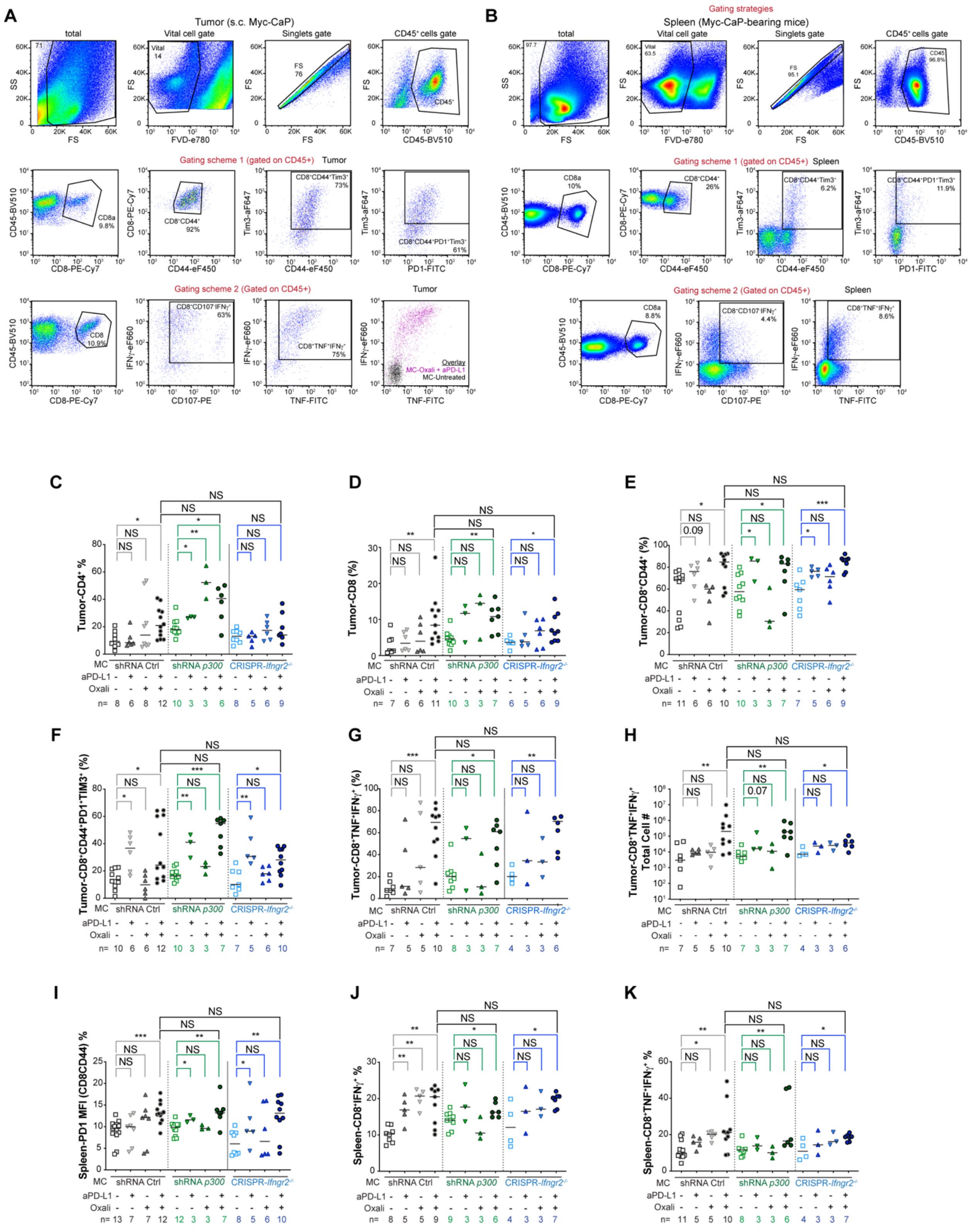
A
Tumor (s.c. Myc-CaP)
B
Gating strategies
Spleen (Myc-CaP-bearing mice)
total
Vital cell gate
Singlets gate
CD45⁺ cells gate
Gating scheme 1 (gated on CD45+)
Gating scheme 2 (Gated on CD45+)
Overlay
MC-Oxali + aPD-L1
MC-Untreated
C
D
E
F
G
H
I
J
K
shRNA Ctrl
shRNA p300
CRISPR-Ifngr2-/-
MC
aPD-L1
Oxali
NS

Fig. S16. Flow cytometric analysis of tumor and spleen T cells subsets**.** (A-B) Single splenocyte or Myc-CaP tumor cell suspensions were stained for CD45, CD8, CD44, PD-1 and Tim-3 or CD45, CD8, TNF, IFNγ and CD107a. FVD-eF780 was used to exclude dead cells. The gating scheme for tumor cells (A) and splenocytes (B) is shown. Plots depict overlay comparison the percentages of $CD8^+$ cells expressing TNF and IFNγ isolated from Oxali + anti-PD-L1-treated and untreated tumor-bearing mice is shown. The analyses of the corresponding groups are presented in Fig. 7M-7N, S16C-16K. (C-K) Single spleen and tumor cell suspensions were analyzed by flow cytometry for the percentage of TI-$CD4^+$ (C) and TI-$CD8^+$ (D), and effector $CD8^+$ T cell subsets in spleen and tumors as indicated (E-K). *$P < 0.05$; **$P < 0.01$; ***$P < 0.001$; NS, not significant. Specific *n* values are shown in (C-K).

Fig. S17

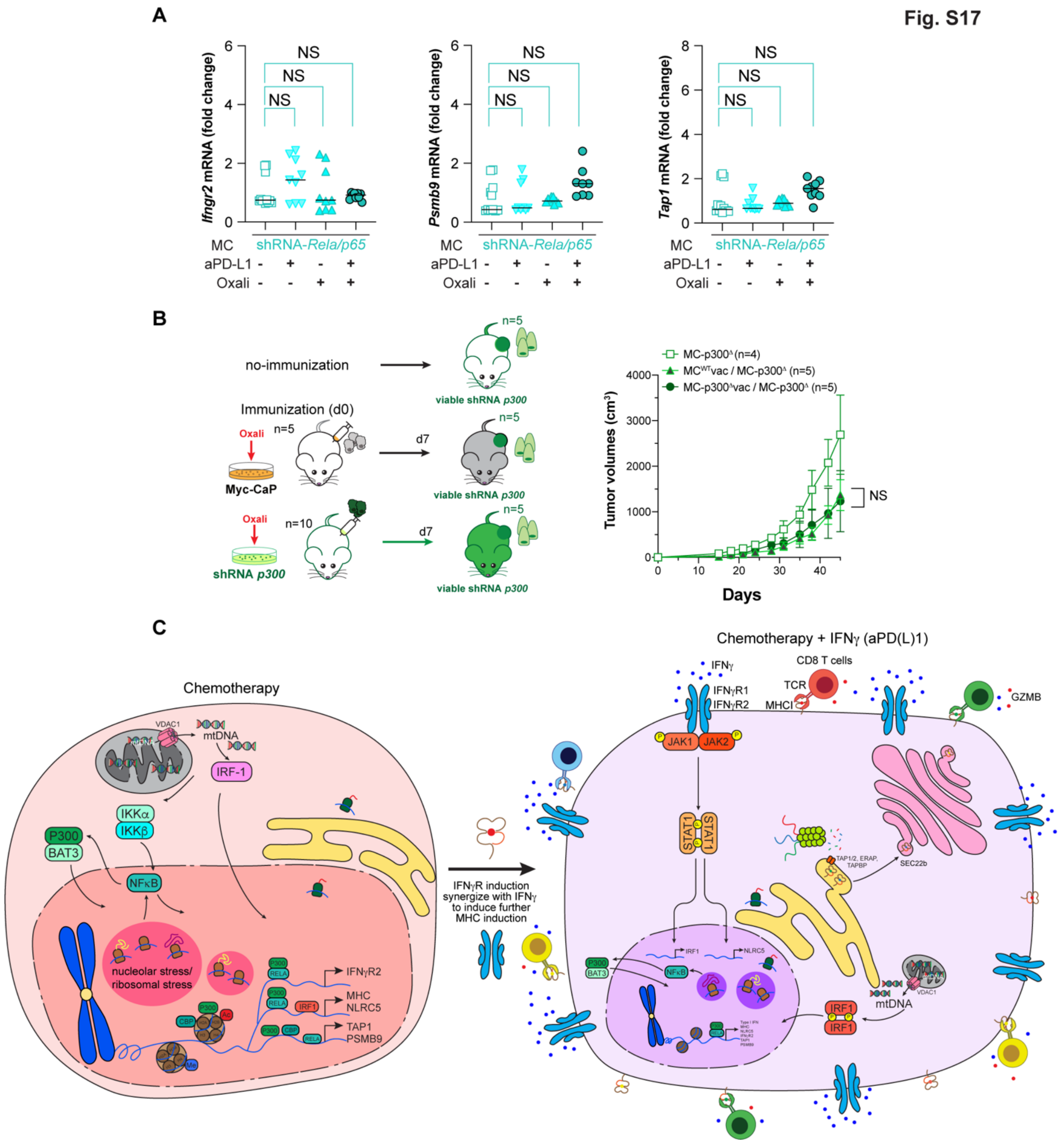
A
Ifngr2 mRNA (fold change)
Psmb9 mRNA (fold change)
Tap1 mRNA (fold change)
NS
MC
shRNA-Rela/p65
aPD-L1
Oxali
B
no-immunization
Immunization (d0)
Oxali
Myc-CaP
shRNA p300
n=5
n=10
d7
viable shRNA p300
MC-p300Δ (n=4)
MCWTvac / MC-p300Δ (n=5)
MC-p300Δvac / MC-p300Δ (n=5)
Tumor volumes (cm3)
Days
C
Chemotherapy
VDAC1
mtDNA
IRF-1
IKKα
IKKβ
P300
BAT3
NFκB
nucleolar stress/
ribosomal stress
IFNγR2
MHC
NLRC5
TAP1
PSMB9
IFNγR induction
synergize with IFNγ
to induce further
MHC induction
Chemotherapy + IFNγ (aPD(L)1)
IFNγ
IFNγR1
IFNγR2
CD8 T cells
TCR
MHCI
GZMB
JAK1
JAK2
STAT1
TAP1/2, ERAP,
TAPBP
SEC22b
IRF1
NLRC5
IRF1
mtDNA
VDAC1

Fig. S17. Oxali-enhanced immune rejection requires NF-κB signaling and summary. (A) Mice bearing s.c. Myc-CaP tumors generated by control and *Rela*-silenced cells were allocated into 4 treatment groups as indicated. Total tumor RNA was analyzed by qRT-PCR for expression of *Ifngr2*, *Psmb9*, and *Tap1* mRNAs. Mann–Whitney test (median) were used to determine significance. (B) Scheme of vaccination experiments (left). Two groups of mice were immunized with lysates of Oxali-killed control (MC^{wt}) and *p300*-silenced ($MC\text{-}p300^{\Delta}$) Myc-CaP cells. After 7 days, the mice were s.c. inoculated with live $MC\text{-}p300^{\Delta}$ Myc-CaP cells (n = 5 per group). Live $MC\text{-}p300^{\Delta}$ Myc-CaP cells were also implanted into non-immunized mice as a control. Tumor Growth curves are shown (right). Two-sided t-test (means ± s.e.m), and two-way ANOVA were used to determine significance. One-way ANOVA analysis and multiple comparison confirmed the results. $^{*}P < 0.05$; $^{**}P < 0.01$; $^{***}P < 0.001$; NS, not significant. Specific *n* values are shown in (A, B). (C) A schematic summarizing the different signaling responses triggered by low dose chemotherapy and leading to MHC-I AgPPM induction and increased IFNγ responsiveness.

SI Appendix Tables:

Table S1: Comparison of CNVs occurrence in MHC-I AgPPM genes between *EP300/CBP* LOF and non-LOF patients in PRAD and LIHC.

	**PRAD**				**LIHC**			
	EP300/CBP		Fisher's exact test	Pearson's chi^2 test	*EP300/CBP*		Fisher's exact test	Pearson's chi^2 test
	NON-LOF (n=410)	LOF (n=74)			NON-LOF (n=188)	LOF (n=161)		
**Group 1 (*HLA* / *PSMB* / *TAP*)**								
Gr-1 loss	19 (4.6%)	0 (0%)	9.45E-02 NS	**6.31E-05 *****	4 (2%)	4 (2.5%)	1.00E+00 NS	**8.40E-04 *****
Gr-1 No CNV	314 (76%)	44 (59%)	**3.66E-03 *****		97 (51%)	51 (32%)	**2.13E-04 *****	
Gr-1 gain	77 (19%)	30 (40%)	**1.04E-04 *****		87 (46%)	106 (66%)	**3.51E-04 *****	
**Group 2 (S*TAT1* / *IFNGR1* / *IFNGR2*)**								
Gr-2 loss	16 (4%)	8 (11%)	**1.92E-02 ***	**1.62E-06 *****	36 (19%)	28 (17%)	6.80E-01 NS	**1.13E-06 *****
Gr-2 No CNV	313 (76%)	35 (47%)	**1.09E-06 *****		89 (47%)	37 (23%)	**2.39E-06 *****	
Gr-2 gain	81 (20%)	31 (42%)	**8.16E-05 *****		63 (34%)	96 (60%)	**1.15E-06 *****	
B2M								
B2M loss	14 (3%)	4 (5%)	4.99E-01 NS	**3.88E-04 *****	7 (4%)	6 (4%)	1.00E+00 NS	**1.02E-03 ****
B2M no CNV	333 (81%)	45 (61%)	**2.13E-04 *****		128 (68%)	79 (49%)	**4.51E-04 *****	
B2M gain	63 (15%)	25 (34%)	**4.52E-04 *****		53 (28%)	76 (47%)	**3.54E-04 *****	
NLRC5								
NLRC5 loss	54 (13%)	15 (20%)	1.46E-01 NS	**9.33E-03 ***	28 (15%)	33 (20%)	2.03E-01 NS	**4.50E-03 ****
NLRC5 No CNV	299 (73%)	41 (55%)	**3.53E-03 ****		117 (62%)	72 (45%)	**1.23E-03 ****	
NLRC5 gain	57 (14%)	18 (24%)	**3.46E-02 ***		43 (23%)	56 (35%)	**1.71E-02 ***	

CNV: copy number variation, gain: gain one or more additional copies of a gene, loss: loss one or both copies of a gene, No CNV: the number of copies for the gene does not vary from expected (two copies), LOF: patients with loss of function in *CREBBP* (*CBP*) and/or *EP300* caused by gene alteration including deleterious single nucleotide variants and/or CNV deletion (Methods).

Table S2: Reagents and Materials

REAGENT or RESOURCE	SOURCE	IDENTIFIER
**Antibodies**		
Rat monoclonal anti-mouse CD366 (Tim3) AlexaF647 (clone B8.2C12)	Biolegend	Cat#: 134006; RRID: AB_1626175
Rat monoclonal anti-mouse CD8α PerCP-Cy5.5 (clone 53-6.7)	Thermo Fisher Scientific	Cat#: 45-0081-82; RRID: AB_1107004
Rat monoclonal anti-mouse CD8α PE-Cy7 (clone 53-6.7)	Thermo Fisher Scientific	Cat#: 25-0081-81; RRID: AB_1107004
Rat monoclonal anti-mouse CD45 Brilliant Violet 510 (clone 30-F11)	Biolegend	Cat#: 103138; RRID: AB_2563061
Rat monoclonal anti-mouse CD45 PE (clone 30-F11)	Thermo Fisher Scientific	Cat#: 12-0451-81; RRID: AB_465667
Rat monoclonal anti-mouse CD44 PE (IM7)	Thermo Fisher Scientific	Cat#: 12-0441-83; RRID: AB_465665
Rat monoclonal anti-mouse CD44 FITC (IM7)	Thermo Fisher Scientific	Cat#: 11-0441-85; RRID: AB_465046
Rat monoclonal anti-mouse CD44 eF450 (IM7)	Thermo Fisher Scientific	Cat#: 48-0441-82; RRID: AB_1272246
Rat monoclonal anti-mouse CD4 eF450 (clone RM4-5)	Thermo Fisher Scientific	Cat#: 48-0042-80; RRID: AB_1272231
Rat monoclonal anti-mouse CD4 FITC (clone GK1.5)	Thermo Fisher Scientific	Cat#: 11-0041-86; RRID: AB_464894
Rat monoclonal anti-mouse CD4 PE (clone GK1.5)	Thermo Fisher Scientific	Cat#: 12-0041-85; RRID: AB_465508
Rat monoclonal anti-mouse CD4 PE-Cy7 (clone GK1.5)	Thermo Fisher Scientific	Cat#: 25-0041-81; RRID: AB_469575
Rat monoclonal anti-mouse CD223 (Lag3) PE (clone C9B7W)	Thermo Fisher Scientific	Cat#: 12-2231-81; RRID: AB_494216
Hamster monoclonal anti-mouse CD3e (clone 145-2C11)	Thermo Fisher Scientific	Cat#: 17-0031-83; RRID: AB_469316
Rat monoclonal anti-mouse TNFα PE (clone MP6-XT22)	Thermo Fisher Scientific	Cat#: 12-7321-81; RRID: AB_466198
Rat monoclonal anti-mouse TNFα FITC (clone MP6-XT22)	Thermo Fisher Scientific	Cat#: 11-7321-81; RRID: AB_465417
Rat monoclonal anti-mouse IFNγ eF660 (clone XMG1.2)	Thermo Fisher Scientific	Cat#: 50-7311-82; RRID: AB_11217680
Rat monoclonal anti-mouse GrzB eF660 (clone NGZB)	Thermo Fisher Scientific	Cat#: 50-8898-82; RRID: AB_11219679
Rat monoclonal anti-mouse CD107a PE (clone eBio1D4B)	Thermo Fisher Scientific	Cat#: 12-1071-82; RRID: AB_657554
Hamster monoclonal anti-mouse PD-1 FITC (clone J43)	Thermo Fisher Scientific	Cat#: 11-9985-85; RRID: AB_465473
Mouse monoclonal anti-mouse H-2Kb eF450 (Clone AF6-88.5.5.3)	Thermo Fisher Scientific	Cat#: 48-5958-80; RRID: AB_10804025
Mouse monoclonal anti-mouse H-2Kq Alexa Fluor 647 (aF647, Clone KH114)	Biolegend	Cat#: 115106; RRID: AB_893562
Mouse monoclonal anti-mouse H-2Kq FITC (clone KH114)	Biolegend	Cat#: 115104; RRID: AB_313625
Mouse monoclonal anti-mouse H-2Kd/H-2Dd APC (Clone 34-1-2S)	Thermo Fisher Scientific	Cat#: 17-5998-82; RRID: AB_2573250
Rat monoclonal anti-mouse PD-1 APC (clone RMP1-30)	Thermo Fisher Scientific	Cat#: 17-9981-80; RRID: AB_10853186
Rat monoclonal anti-mouse PD-L1 PE-Cy7 (clone MIH5)	Thermo Fisher Scientific	Cat#: 25-5982-82; RRID: AB_2573509
Rat monoclonal anti-mouse PD-L1 PE (clone MIH5)	Thermo Fisher Scientific	Cat#: 12-5982-83; RRID: AB_466090
Mouse monoclonal anti-human PD-L1 PE-Cy7 (clone MIH1)	Thermo Fisher Scientific	Cat#: 25-5983-41; RRID: AB_1907369
Rat monoclonal anti-mouse Ki-67 eF450 (clone SolA15)	Thermo Fisher Scientific	Cat#: 48-5698-82; RRID: AB_11149124
Rat monoclonal anti-mouse CD11b PE-Cy7 (clone M1/70)	Thermo Fisher Scientific	Cat#: 25-0112-82; RRID: AB_469588

Hamster monoclonal anti-mouse CD11c eF450 (clone N418)	Thermo Fisher Scientific	Cat#: 48-0114-82; RRID: AB_1548654
Armenian hamster anti-mouse CD11c PE (clone N418)	Thermo Fisher Scientific	Cat#: 12-0114-82; RRID: AB_465552
Rat monoclonal anti-mouse MHC II FITC (clone M5/114.15.2)	Thermo Fisher Scientific	Cat#: 11-5321-85; RRID: AB_465233
Rat monoclonal anti-mouse MHC II PE (clone M5/114.15.2)	Thermo Fisher Scientific	Cat#: 12-5321-83; RRID: AB_465929
Rat monoclonal anti-mouse Gr-1 PerCP eF710 (clone 1A8-ly6g)	Thermo Fisher Scientific	Cat#: 46-9668-82; RRID: AB_2573893
Rat monoclonal anti-mouse Perforin FITC (clone eBioOMAK-D)	Thermo Fisher Scientific	Cat#: 11-9392-82; RRID: AB_465447
Mouse monoclonal anti-mouse SIINFEKL / H-2Kb APC	Thermo Fisher Scientific	Cat#: 17-5743-80; RRID: AB_1311288
Mouse monoclonal anti-mouse HMGB1 PE (clone 3E8)	Biolegend	Cat#: 651403; RRID: AB_2562220
Rat monoclonal anti-mouse F4/80 PE (clone BM8)	Thermo Fisher Scientific	Cat#: 12-4801-82; RRID: AB_465923
Rat monoclonal anti-mouse F4/80 FITC (clone BM8)	Thermo Fisher Scientific	Cat#: 11-4801-85; RRID: AB_2637192
Rabbit monoclonal anti-mouse/human phosphor-CREB (Ser133) (87G3) Alexa 647	Cell Signaling	Cat#: 14001; RRID: AB_2798359
Mouse monoclonal anti-human HLA-A2 APC (clone BB7.2)	eBioscience	Cat#: 17-9876-41 RRID: AB_11151522
Mouse monoclonal anti-human HLA-ABC PE-Cyanine7 (clone W6/32)	eBioscience	Cat#: 25-9983-41 RRID: AB_2573569
Anti-biotin PE (clone BK-1/39)	Thermo Fisher Scientific	Cat#: 12-9895-82; RRID: AB_10598675
Alexa 594 Donkey Anti-Rabbit IgG	Thermo Fisher Scientific	Cat#: A-21207; RRID: AB_141637
Alexa 647 Donkey Anti-Rabbit IgG	Thermo Fisher Scientific	Cat#: A-31573; RRID: AB_2536183
Alexa 488 Donkey Anti-Rabbit IgG	Thermo Fisher Scientific	Cat#: A-21206; RRID: AB_141708
Alexa 594 Donkey Anti-Mouse IgG	Thermo Fisher Scientific	Cat#: A-21203; RRID: 141633
Alexa 488 Donkey Anti-Mouse IgG	Thermo Fisher Scientific	Cat#: A-21202; RRID: 141607
Alexa Fluor 568 phalloidin	Life Technologies	Cat#: A12380
Rabbit monoclonal anti-human Prostate Specific Antigen (PSA) (1:1)	NeoMarkers	Cat#: RM-2104-R7
Rabbit polyclonal anti-mouse/human ARTS1 (ERAP1) (1:200)	Proteintech	Cat#: 13821-1-AP; RRID: AB_2293531
Rabbit polyclonal anti-human TAP1 (1:200)	Proteintech	Cat#: 11114-1-AP; RRID: AB_2200201
Rabbit polyclonal anti-human HLA-A (1:150)	Proteintech	Cat#: 15240-1-AP; RRID: AB_1557426
Mouse monoclonal anti-human HLA-ABC	Invitrogen	MA5-13582 RRID: AB_10979940
Mouse monoclonal anti-human CD45 (clone 4E9B2) (1:200)	Proteintech	Cat#: 60287-1-Ig
Rabbit polyclonal anti-mouse/human CREB1 (1:1000)	Proteintech	Cat#: 12208-1-AP RRID: AB_2245417
Rabbit monoclonal anti-mouse/human p300 (1:1000)	Cell Signaling	Cat#: 86377; RRID: AB_2800077
Rabbit polyclonal anti-mouse/human Acetyl-CBP (Lys1535) / p300 (Lys1499) (1:1000)	Cell Signaling	Cat#: 4771; RRID: AB_2262406
Rabbit monoclonal anti-mouse CBP (D6C5)	Cell Signaling	Cat#: 7389; RRID: AB_2616020
Mouse monoclonal anti-LC3	Cosmo Bio	Cat#: CAC-CTB-LC3-2-I
Mouse monoclonal anti-human αSMA Clone 1A4 (1:300)	DAKO	Cat#: M0851; RRID: AB_2223500
Rabbit polyclonal anti-mouse Beta2 microglobulin	Genetex	Cat#: GTX112815; RRID: AB_11175135
Mouse polyclonal anti-E2F1 (18E10)	GeneTex	Cat#: GTX70165; RRID: AB_370186
Rabbit polyclonal anti-mouse/human NFκB RELA/p65 (Acetyl K310)	GeneTex	Cat#: GTX86963; RRID: AB_10726079

Rabbit polyclonal anti-mouse/human NFκB RELA/p65 (Phospho Ser536)	GeneTex	Cat#: GTX50098; RRID: AB_11177842
Rabbit monoclonal anti-mouse/human NFκB RELA/p65 (D14E12) XP (1:1000)	Cell Signaling	Cat#: 8242; RRID: AB_10859369
Rabbit polyclonal anti-mouse/human Phospho-NFκB PELA/p65 (Ser536) (1:1000)	Cell Signaling	Cat#: 3031; RRID: AB_330559
Rabbit polyclonal anti-mouse/human NFkB RELA/p65 (C-20) (1:500)	Santa Cruz	Cat#: sc-372; RRID: AB_632037
Mouse monoclonal anti-mouse/human CHOP (9C8) (1:1000)	Thermo Fisher Scientific	Cat#: MA1-250; RRID: AB_2292611
Mouse monoclonal anti-mouse/human Phospho-ATM (Ser1981) Monoclonal (10H11) (1:1000)	Thermo Fisher Scientific	Cat#: MA1-2020; RRID: AB_1086244
Rabbit polyclonal anti-mouse/human Phospho-p53 (Ser15) (1:1000)	Cell Signaling	Cat#: 9284; RRID: AB_ 331464
Rabbit monoclonal anti-mouse/human p53 (1C12) (1:1000)	Cell Signaling	Cat#: 2524; RRID: AB_331743
Rabbit monoclonal anti-mouse/human ATF-3 (D2Y5W) (1:1000)	Cell Signaling	Cat#: 33593; RRID: AB_2799039
Rabbit monoclonal anti-mouse/human JunB (C37F9) (1:1000)	Cell Signaling	Cat#: 3753; RRID: AB_2130002
Rabbit monoclonal anti-mouse cGAS (D3080) (1:1000)	Cell Signaling	Cat#: 31659; RRID: AB_2799008
Rabbit monoclonal anti-mouse/human IRF-1 (D5E4) XP (1:1000)	Cell Signaling	Cat#: 8487; RRID: AB_10949108
Mouse monoclonal anti-mouse/human eIF2α (C-21)	Santa Cruz	Cat#: sc-133132; RRID: AB_1562699
Rabbit monoclonal anti-mouse/human Phospho-eIF2α (Ser51) (119A11) (1:1000)	Cell Signaling	Cat#: 3597; RRID: AB_390740
Rabbit monoclonal anti-mouse/human anti-H3K27Ac, Acetyl-Histone H3 (Lys27) (D5E4) XP (1:1000)	Cell Signaling	Cat#: 8173; RRID: AB_10949503
Rabbit monoclonal anti-mouse/human anti-H3K9Ac, Acetyl-Histone H3 (Lys9) (C5B11) (1:1000)	Cell Signaling	Cat#: 9649; RRID: AB_823528
Rabbit monoclonal anti-mouse/human Acetyl-Histone H3 (Lys14) (1:1000)	Cell Signaling	Cat#: 7627; RRID: AB_10839410
Rabbit Monoclonal Anti-mouse/human Phospho-Histone H2A.x (Ser139) (20E3) (1:1000)	Cell Signaling	Cat#: 9718; RRID: AB_2118009
Rabbit polyclonal anti-Normal Rabbit IgG (1:1000)	Cell Signaling	Cat#: 2729; RRID: AB_1031062
Rabbit monoclonal anti-mouse/human Lamin B1 (D9V6H)	Cell Signaling	Cat#: 13435; RRID: AB_2737428
Rabbit polyclonal anti-mouse Acetyl-p53 (Lys379) (1:200)	Cell Signaling	Cat#: 2570; RRID: AB_823591
Rabbit monoclonal anti-mouse VDAC (D73D12)	Cell Signaling	Cat#: 4661; RRID: AB_10557420
Rabbit monoclonal anti-mouse phospho-IRF-3 (Ser396) (4D4G)	Cell Signaling	Cat#: 4947; RRID: AB_823547
HRP linked anti-mouse IgG antibody	Cell Signaling	Cat#: 7046; RRID: AB_330924
HRP linked anti-rabbit IgG antibody	Cell Signaling	Cat#: 7074; RRID: AB_2099233
Rabbit polyclonal anti-mouse/human HDAC1 (H-51) (1:500)	Santa Cruz	Cat#: sc-7872; RRID: AB_2279709
Mouse monoclonal anti-mouse/human LMP2 (G-3)	Santa-Cruz	Cat#: sc-373996; RRID: AB_10918476
Rabbit polyclonal anti-mouse/human IκB-α (C-21)	Santa Cruz	Cat#: sc-371; RRID: AB_2235952
Rabbit polyclonal anti-mouse/human Stat1 p84/p91	Santa Cruz	Cat#: sc-346; RRID: AB_632435
Mouse monoclonal anti-mouse/human LMP7 (A-12)	Santa Cruz	Cat#: sc-365699; RRID: AB_10846323
Rabbit polyclonal anti-mouse/human phospho-STAT1 (Tyr701)	MilliporeSigma	Cat#: 07-307; RRID: AB_310507
Rabbit polyclonal anti-mouse/human Histone H3	ABclonal	Cat#: A-2348; RRID: AB_2631273
Mouse monoclonal anti-mouse/human Anti-α-Tubulin (1:2000)	Sigma-Aldrich	Cat#: T5168; RRID: AB_477579
Rabbit polyclonal anti-mouse/house anti-beta 2 microglobulin	GeneTex	Cat#: GTX112815; RRID: AB_11175135
Y3 hybridoma (H-2Kb)	ATCC	Cat# HB-176, RRID: CVCL_E990
Anti-Mouse H-2Db, (mouse IgG2a)	Yadav et al.	B22.249 clone

Mouse monoclonal anti-Myc tag	Abcam	Cat#: ab32; RRID: AB_303599
Mouse monoclonal anti-HA probe	Santa Cruz	Cat#: sc-7392; RRID: AB_627809
Rabbit polyclonal anti-mouse/human IRF7	Zymed	Cat#: 51-3300; RRID: AB_87191
Rabbit polyclonal anti-mouse TMEM173/STING	Proteintech	Cat#: 19851-1-AP; RRID: AB_10665370
Mouse monoclonal anti mouse/human p300 (F-4) (1:100)	Santa Cruz	Cat#: sc-48343; RRID: AB_628075
Rabbit monoclonal anti mouse BAT3/BAG-6 (EPR9223) (1:500)	Abcam	Cat#: ab137076
**Bacterial and Virus Strains**		
Max Efficiency Stbl2 Competent Cells	ThermoFisher Scientific	Cat#: C404003
**Chemicals, Peptides, and Recombinant Proteins**		
Mouse IL-7 Recombinant Protein	ebioscience	Cat#: 14-8071-62
Mouse IL-2 Recombinant Protein	ebioscience	Cat#: 14-8021-64
Mouse IFNγ Cytokine	GoldBio	Cat#: 1360-02-20
Human IFNγ Cytokine	GoldBio	Cat#: 1160-06-20
Ac-ANW-AMC	Boston Biochem	Cat#: S-320
Protein A agarose	Upstate	Cat#: 16-125
Protein A dynabeads	Invitrogen	Cat#: 10001D
Proteinase K	Thermo Fisher Scientific	Cat#: 26160
RNAse A	Thermo Fisher Scientific	Cat#: EN0531
IGEPAL CA-630	Sigma-Aldrich	Cat#: I8896
DTT	Sigma-Aldrich	Cat#: D9779
Protease inhibitor	Roche	Cat#: 05056489001
5% BSA	Sigma-Aldrich	Cat#: A7906
Tris-acetate	Thermo Fisher Scientific	Cat#: BP-152
K-acetate	Sigma-Aldrich	Cat#: P5708
Mg-acetate	Sigma-Aldrich	Cat#: M2545
16 % DMF	EMD Millipore	Cat#: DX1730
Molecular biology water	Corning	Cat#: 46000-CM
Tagmentation enzyme	Illumina	Cat#: FC-121-1030
AllPrep DNA/RNA Mini Kit	Qiagen	Cat#: 80204
MinElute PCR purification kit	Qiagen	Cat#: 28004
Mitochondria Isolation Kit for Cultured Cells	Thermo Fisher Scientific	Cat#: 89874
NEBNext High-Fidelity 2X PCR Master Mix	NEB	Cat#: M0541
SPRIselect bead	Beckman Coulter	Cat#: B23317
Protein A sepharose resin	Repligen	CA-PRI
DMP (dimethyl pimelimidate)	Thermo Fisher Scientific	Cat#: 21666
Luna C18	Phenomenex	Cat#: 00G-4094-E0
CD8a(Ly-2) Microbeads, mouse	Miltenyi Biotec	Cat#: 13-117-044
Anti-HA magnetic Beads	MedChemExpress	Cat#: HY-K0201
Glutaraldehyde	Ted Pella Inc	Cat#: 18426
Sodium cacodylate buffer	Ted Pella Inc	Cat#: 18851
Osmium tetroxide	Electron Microscopy Sciences	Cat#: 19150
Uranyl acetate	Electron Microscopy Sciences	Cat#: 22400
Durcupan ACM epoxy resin	Sigma-Aldrich	Cat#: 44610
**Cell culture**		
Dulbecco's phosphate buffered saline without Ca^2+, Mg^2+	Gibco	Cat#: 14190-144

PBS	Thermo Fisher Scientific	Cat#: 10010-23
0.5 M EDTA	Invitrogen	Cat#: 15575020
Heat Inactivated Fetal Bovine Serum (FBS)	Gibco	Cat#: 10437-028
Dulbecco's Modified Eagle Medium (DMEM)	Gibco	Cat#: 11965-092
DMEM/F12 Medium	Gibco	Cat#: 12364-010
RPMI 1640 Medium	Gibco	Cat#: 11875093
Trypan Blue 0.4%	Gibco	Cat#: 15250061
Trypsin-EDTA (0.25%)	Gibco	Cat#: 25200-056
L-Glutamine	Life Tech	Cat#: 25030-081
Penicillin/Streptomycin	Gibco	Cat#: 15140-122
Ampicillin, Sodium Salt	Sigma Aldrich	Cat#: A9518-100G
Puromycin	InvivoGen	Cat#: A11138-03
G-418 Sulfate	Gold Biotechnology	Cat#: G-418-25
Polybrene (Hexadimethrine bromide)	Sigma Aldrich	Cat#: H9268
Acutase	eBioscience	Cat#: 00-4555-56
NuSerum IV	Corning	Cat#: 355104
Dehydroisoandrosterone	Acros Organics	Cat#: AC154980025
Opti-MEM	Gibco	Cat#: 31985070
Bovine Insulin	Sigma-Aldrich	Cat#: 10516-5ML
Doxycycline hyclate	Sigma-Aldrich	Cat#: D9891-100G
EZ-PCR Mycoplasma Detection Kit	Biological Industries	Cat#: 20-700-20
**Flow cytometry**		
Cell Stimulation Cocktail	ebioscience	Cat#: 00-4970-03
4′,6-diamidino-2-phenylindole (DAPI)	ThermoFisher Scientific	Cat#: D1306
10x RBC Lysis Buffer	ebioscience	Cat#: 00-4300-54
10x BD Perm/Wash	BD Biosciences	Cat#: 554723
Cell staining buffer	Biolegend	Cat#: 420201
Collagenase I	Gibco	Cat#: 17100-017
Collagenase IV	Gibco	Cat#: 17104-019
DNase I	Stemcell	Cat#: 07900
Protein Transport Inhibitor	ThermoFisher Scientific	Cat#: 00-4980-03
Foxp3/Transcription Factor Staining Buffer Kit	ebioscience	Cat#: 00-5523-00
Transcription Factor Buffer Set	BD Biosciences	Cat#: 562574
Transcription Factor Phospho Buffer Set	BD Sciences	Cat#: 563239
Corning CellStripper Dissociation Reagent	Corning	Cat#: MT25026CI
Fixable Viability Dye eF780	eBioscience	Cat#: 65-0865-14
**Histology**		
Tissue-Tek OCT Compound	Sakura Finetek USA, Inc.	Cat#: 4583
PFA	Electron Microscopy Sciences	Cat#: 15710
CellLight Golgi-GFP, BacMam 2.0	Life Technologies	Cat#: C10592
ImmPRESS-AP REAGENT Anti-Rabbit IgG	Vector Laboratories	Cat#: MP-5401
ImmPRESS-AP REAGENT Anti-Mouse IgG	Vector Laboratories	Cat#: MP-5402
ImmPRESS-HRP REAGENT Anti-Rabbit IgG	Vector Laboratories	Cat#: MP-7451
DAB	Vector Laboratories	Cat#: SK-4100
ImmPACT Vector Red	Vector Laboratories	Cat#: SK-5105

Nunc Lab Tek 8-well Chamber Slide with Removable Wells	Thermo Fisher Scientific	Cat#: 177402PK
Gelatin from cold water fish skin	Sigma-Aldrich	Cat#: G7765
Vectashield Mounting Medium with DAPI	Vector Laboratories	Cat#: H-1200
**Immunoblot**		
Mini-Protean TGX Precast Gels	BIO-RAD	Cat#: 456-9033
Trans-Blot Turbo Transfer Pack	BIO-RAD	Cat#: 170-4156
0.45 μm pore size, Hydrophobic PVDF Transfer Membrane	Millipore	Cat#: IPVH00010
Halt Protease Inhibitor Cocktail	Pierce Chemicalms	Cat#: PI78439
Halt Phosphatase Inhibitor Cocktail	Pierce Chemicalms	Cat#: PI78427
Sodium Deoxycholate	Sigma-Aldrich	Cat#: D6750
4x Laemmli Sampler Buffer	Bio-Rad	Cat#161-0747
**RNA**		
SsoAdvanced™ Universal SYBR® Green Supermix	Bio-Rad	Cat#: 172-5274
RNeasy Mini Kit	Qiagen	Cat#: 74104
SuperScript VILO cDNA Synthesis Kit	Invitrogen	Cat#: 11754-050
RTScript cDNA Synthesis Kit	Empirical Bioscience	Cat#: RT-CSK-100
TRIzol Reagent	Ambion by Life Technologies	Cat#: 15596026
Chloroform	Fischer Chemical	Cat#: C606-1
2-Propanol	Optima	Cat#: A464-4
Water, RNase-Free DEPC Treated Ultrapure MB Grade	USB Corporation	Cat#: 70783.1
Ethanol, 200 Proof Pure	Koptec	Cat#: V1016
**Cloning**		
Lipofectamine 3000	Invitrogen	Cat#: L3000015
QIAprep Spin Miniprep Kit	Qiagen	Cat#: 27104
Qiagen Plasmid Maxi Kit	Qiagen	Cat#: 12162
QIAquick Gel Extraction Kit	Qiagen	Cat#: 28704
T4 DNA Ligase Buffer	NEB	Cat#: B0202S
T4 DNA Ligase	NEB	Cat#: M0202S
BsmbI Restriction Enzyme	NEB	Cat#: R0580S
BbsI Restriction Enzyme	NEB	Cat#: R0539S
NEBuffer 3.1	NEB	Cat#: B7203S
NEBuffer 2.1	NEB	Cat#: B7202S
psPAX2	Addgene	Cat#: 12260
pMD2.G	Addgene	Cat#: 12259
Millex-HV Low Protein Binding Durapore Membrane	Millipore	Cat#: SLHV033RB
**Treatment**		
Carboplatin	Hospira Inc.	NDC: 61703-339-22
Cisplatin	Teva Pharmaceuticals	NDC: 0703-5747-11
Oxaliplatin	Teva Pharmaceuticals	NDC: 0703-3986-01
Mitoxantrone	Teva Pharmaceuticals	NDC: 00703-4686-01
Dihydroeponemycin (LMP2 inhibitor)	ApexBio	Cat#: A8172
ONX-0914 (PR-957) (LMP7 inhibitor)	Selleckchem	Cat#: s7172
Ikk2 inhibitor IV	Calbiochem	Cat#: 401483
Ikk2 inhibitor ML120B	Sigma-Aldrich	Cat#: SML1174

Anti-PD-L1	Genentech	Shalapour et al.
C646 HAT inhibitor	Selleckchem	Cat#: S7152
KU-55933 ATM Kinase Inhibitor	Selleckchem	Cat#: S1092
VE-821	Selleckchem	Cat#: S8007
Panobinostat (LBH589) HDAC inhibitor	Selleckchem	Cat#: S1030
**Critical Commercial Assays**		
EpiQuik Nuclear Extraction Kit I	Epigentek	Cat#: OP-0002
EpiQuik HAT Activity/Inhibition	Epigentek	Cat#: P-4003
NE-PER™ Nuclear and Cytoplasmic Extraction Kit	ThermoFisher	Cat#: 78835
**Deposited Data**		
Liver RNA-seq (*in vivo*)	GSE90497, Shalapour et al.	https://www.ncbi.nlm.nih.gov/geo/query/acc.cgi?acc=GSE90497
Mouse ATAC-seq (*in-vitro*)	GSE126288 (GSE126287)	https://www.ncbi.nlm.nih.gov/geo/query/acc.cgi?acc=GSE126287
Mouse RNA-seq (*in-vitro*)	GSE126288 (GSE126274)	https://www.ncbi.nlm.nih.gov/geo/query/acc.cgi?acc=GSE126274
Mouse Single Cell RNA-seq (*in vivo*)	GSE151611	https://www.ncbi.nlm.nih.gov/geo/query/acc.cgi?acc=GSE151611
**Experimental Models: Cell Lines**		
Mouse: Myc-CaP PCa	ATCC	Cat#: CRL-3255<br>RRID: CVCL_J703
Mouse: TRAMP-C2 PCa	ATCC	Cat#: CRL-2731<br>RRID: CVCL_3615
Mouse: TRAMP-C2-N4	Shalapour et al.	This Manuscript
Mouse: TRAMP-C2-G4	Shalapour et al.	This Manuscript
Mouse: TRAMP-C2-E1	Shalapour et al.	This Manuscript
Mouse: MC38 colon cancer	Genentech	RRID: CVCL_B288
Mouse: B16-Ova melanoma	ATCC	Cat#: CRL-6475; RRID: CVCL_0159
Mouse: Yumm1.7 melanoma	M. Bosenberg	Meeth et al. RRID: CVCL_JK16
Mouse: Yumm2.1 melanoma	M. Bosenberg	(4) RRID: CVCL_JK30
Mouse: Yumm3.3 melanoma	M. Bosenberg	(4) RRID: CVCL_JK36
Mouse: Yumm4.1 melanoma	M. Bosenberg	Meeth et al. RRID: CVCL_JK38
Mouse: Yumm5.2 melanoma	M. Bosenberg	(4) RRID: CVCL_JK43
Human: MIA PaCa-2	ATCC	Cat#: CRL-1420; RRID: CVCL_0428
Human: PC3	ATCC	Cat#: CRL-1435; RRID: CVCL_0035
Human: WM35	ATCC	Cat#: CRL-2807; RRID: CVCL_0580
Human: WM1366	Rockland	Cat#: WM1366-01-0001; RRID: CVCL_6789
Human: WM3682	Rockland	Cat#: WM3682-01-0001; RRID: CVCL_AP78
Human: WM3702	Rockland	Cat# WM3702-01-0005; RRID: CVCL_0B77
Human: Lu1205	ATCC	Provided by Dr. Ronai<br>RRID: CVCL_5239
Human: A375	ATCC	Provided by Dr. Ronai<br>RRID: CVCL_0132
Human: WM793	ATCC	Cat#: CRL-2806; RRID: CVCL_8787
PCSD1 human PCa 3D organoid	Godebu et al.	C. Jamieson

Experimental Models: Organisms/Strains		
C57BL/6	Charles Rivers Laboratories	Cat#: C57BL/6NCrl
FVB/N	Charles Rivers Laboratories	Cat#: FVB/NCrl
OT-I mice	Hogquist et al.	Taconic Bioscience
Cd8a$^{-/-}$ C57BL/6	Jackson Laboratory	B6.129S2-Cd8atm1Mak/J
**Oligonucleotides**		
Crispr/Cas9 gRNA targeting sequence: Ifngr2 GACGAGGGAACCTTTTCCTG	This Manuscript	http://chopchop.cbu.uib.no
Crispr/Cas9 gRNA targeting sequence: Stat1 GTGAGAGGAGGTCATGGAAG	This Manuscript	http://chopchop.cbu.uib.no
Crispr/Cas9 gRNA targeting sequence: Irf1 CTGTGTGAATGGCCCAGCTC	This Manuscript	http://chopchop.cbu.uib.no
Crispr/Cas9 gRNA targeting sequence: Tap1 GGGTGAACGTCAGCCCCTGG	This Manuscript	http://chopchop.cbu.uib.no
Crispr/Cas9 gRNA targeting sequence: cGAS GAAAGCTGCGGCCCGCAAAG	This Manuscript	http://chopchop.cbu.uib.no
Crispr/Cas9 gRNA targeting sequence: p300 CACCGAACCTCAACATGGGAGTCGG	This Manuscript	http://chopchop.cbu.uib.no
shRNA targeting sequence: p300 CCGGCCCCTGGATTAAGTTTGATAAACTCGAGTTTATCAAACTTAATCCA GGGTTTTTG	LJI-Functional Genomics Lab	Gene ID: 328572 Clone ID: TRCN0000071203
shRNA targeting sequence: Cbp-1 CCGGCGGAGTCATCTAGTCCATAAACTCGAGTTTATGGACTAGATGACT CCGTTTTT	LJI-Functional Genomics Lab	Gene ID: 12914 Clone ID: TRCN0000012724
shRNA targeting sequence: Cbp-4 CCGGCCAACCTCAGACGACAATTTCCTCGAGGAAATTGTCGTCTGAGG TTGGTTTTTG	LJI-Functional Genomics Lab	Gene ID: 12914 Clone ID: TRCN0000231204
shRNA targeting sequence: Rela-1 CCGGCTGTCCTCTCACATCCGATTTCTCGAGAAATCGGATGTGAGAGG ACAGTTTTTG	LJI-Functional Genomics Lab	Gene ID: 19697 Clone ID: TRCN0000055346
shRNA targeting sequence: Rela-2 CCGGGCTCAAGATCTGCCGAGTAAACTCGAGTTTACTCGGCAGATCTT GAGCTTTTTG	LJ- Functional Genomics Lab	Gene ID: 19697 Clone ID: TRCN0000235834
shRNA targeting sequence: Rela-3 CCGGGCATGCGATTCCGCTATAAATCTCGAGATTTATAGCGGAATCGCA TGCTTTTTG	LJI-Functional Genomics Lab	Gene ID: 19697 Clone ID: TRCN0000244319
shRNA targeting sequence: Vdac1 CCGGGTTGGCTATAAGACGGATGAACTCGAGTTCATCCGTCTTATAGCC AACTTTTT	LJI-Functional Genomics Lab	Gene ID: 22333 Clone ID: TRCN0000012391
**Primers for ChIP**		
mChIP_Tap1_1_F1: GCTAGGCAGAACTCCAACTAC mChIP_Tap1_1_R1: GCTGGCGTTTAGAGGAAGAA	This Manuscript	NM_013683
mChIP_Psmb8_F1: CCTAGGTGGATCTGCTCAATAC mChIP_Psmb8_R1: GAACAGCGGAGGACTGAATAG	This Manuscript	NM_010724
mPsmb9_ChIP_F1: TGAAGCTGTAGTTGGAGTTCTG mPsmb9_ChIP_R1: AATTCACGCAAGCAAGTTAAGG	This Manuscript	NM_013585
m18sChip_F: CAGTCGGCATCGTTTATGGTT m18sChip_R: CGGTTCTATTTTGTTGGTTTTCG	This Manuscript	CT010467.1
mIfngr2_ChIP_NFKb_F3: ATGGGTCCTTAAATCGTACGTG mIfngr2_ChIP_NFKb_R3: AGATCTCTAGTACCATCCTTCTGG	This Manuscript	NM_008338
**Primers for qRT-PCR**		
mGapdh F: AGCTTGTCATCAACGGGAAG *mGapdh* R: TTTGATGTTAGTGGGGTCTCG	This manuscript	NM_008084
mActb F: AGTGTGACGTTGACATCCGT *mActb* R: GCAGCTCAGTAACAGTCCGC	This manuscript	NM_007393
mTap1 F: CCCAGCAGGTTCCATCACAT *mTap1* R: GAAAAAGCAGGGGCAGGTTG	This manuscript	NM_013683
mIfngr2 F: CTCGCCAGACTCGTTTTCCC *mIfngr2* R: GTTCGGCTCCAGCAACCTAT	This manuscript	NM_008338

mErap1 F: GTTGAACACAACGGAGCTGA<br>*mErap1* R: GGCTCATCGTCCATAGCTCA	This manuscript	NM_030711
mPsmb9 F: GAAGAAGTCCACACCGGGAC<br>*mPsmb9* R: GAGGGGAGAGCTTGTCGAAC	This manuscript	NM_013585
mNlrc5 F: GACGCTGGGGTTAACAGGAA<br>*mNlrc5* R: CAGCTCCACAAGACTCAGCA	This manuscript	NM_001033207
mTapbp F: TTATGGGTGAGGACGGTCAG<br>*mTapbp* R: CCAGCACTCTCTTCAGCCTC	This manuscript	NM_001025313
mIl1b F: AGTTGACGGACCCCAAAAG<br>*mIl1b* R: AGCTGGATGCTCTCATCAGG	This manuscript	NM_008361
mNfkbia F: GTCTCCCTTCACCTGACCAA<br>*mNfkbia* R: CAGCAGCTCACGGAGGAC	This manuscript	NM_010907.2
mIfna F: ACCCAGCAGATCCTGAACAT<br>*mIfna* R: AATGAGTCTAGGAGGGTTGTA	This manuscript	NM_010502.2
mP300 F: GAGGTGTTGGGGTTCAGACG<br>*mP300* R: GCCACACCAGCATTTTCACT	https://www.ncbi.nlm.nih.gov/tools/primer-blast/index.cgi	NM_177821.6
mCbp F: TGGAAGAACTGCACACGACA<br>*mCbp* R: GGAAGTGGCATTCTGTTGCC	https://www.ncbi.nlm.nih.gov/tools/primer-blast/index.cgi	NM_001025432.1
mH2kq F: CGCCCTGAACGAAGACCTGAAA<br>*mH2kq* R: CACCACCACAGATGCCCACTTC	https://www.ncbi.nlm.nih.gov/tools/primer-blast/index.cgi	MF352193.1
mIrf1 F: AATTCCAACCAAATCCCAGG<br>*mIrf1* R: AGGCATCCTTGTTGATGTCC	https://www.ncbi.nlm.nih.gov/tools/primer-blast/index.cgi	NM_008390.2
mSec22b F: CTGACGATGATCGCCCGTG<br>*mSec22b* R: TGCTTAGCCTGACTCTGATACTG	https://www.ncbi.nlm.nih.gov/tools/primer-blast/index.cgi	NM_011342.4
mBat3 F: CAACAGCACCAACTCGGGT<br>*mBat3* R: TCTGGGCCAATGAAGTGTTTG	https://www.ncbi.nlm.nih.gov/tools/primer-blast/index.cgi	NM_057171.3
mRela F: AGGCTTCTGGGCCTTATGTG<br>*mRela* R: TGCTTCTCTCGCCAGGAATAC	https://www.ncbi.nlm.nih.gov/tools/primer-blast/index.cgi	NM_009045.5
mVdac1 F: ACTAATGTGAATGACGGGACA<br>*mVdac1* R: GCATTGACGTTCTTGCCAT	https://www.ncbi.nlm.nih.gov/tools/primer-blast/index.cgi	NM_001362693.1
mD-loop F: AATCTACCATCCTCCGTGAAACC<br>*mD-loop* R: TCAGTTTAGCTACCCCCAAGTTTAA	Zhong et al.	NC_005089.1
mTert F: CTAGCTCATGTGTCAAGACCCTCTT<br>*mTert* R: GCCAGCACGTTTCTCTCGTT	Zhong et al.	NM_009354.2
mB2m F: ATGGGAAGCCGAACATACTG<br>*mB2m* R: CAGTCTCAGTGGGGGTGAAT	Zhong et al.	NM_009735.3
non-NUMT F: CTAGAAACCCCGAAACCAAA<br>*non-NUMT* R: CCAGCTATCACCAAGCTCGT	Zhong et al.	NC_005089.1
hGAPDH F: AATGGGCAGCCGTTAGGAAA<br>*hGAPDH* R: GCCCAATACGACCAAATCAGAG	This manuscript	NM_001256799
hACTB F: ACAGAGCCTCGCCTTTGCC<br>*hACTB* R: GATATCATCATCCATGGTGAGCTGG	This manuscript	NM_001101
hTAP1 F: TGCCCCGCATATTCTCCCT<br>*hTAP1* R: CACCTGCGTTTTCGCTCTTG	This manuscript	NM_000593
hIFNGR2 F: AACAATGGCAGATGCCTCCA<br>*hIFNGR2* R: GGATGCTTGGTGGAGTGTGA	This manuscript	NM_005525
hPSMB9 F: CGCTTCACCACAGACGCTAT<br>*hPSMB9* R: CCACACCGGCAGCTGTAATA	This manuscript	NM_002800
hNLRC5 F: GCTCGGCAACAAGAACCTGT<br>*hNLRC5* R: GGTCCAAGGTCTCGTTCCT	This manuscript	NM_032206
hEP300 F: GCTTCAGACAAGTCTTGGCAT<br>*hEP300* R: ACTACCAGATCGCAGCAATTC	This manuscript	NM_001429
**Recombinant DNA**		
lentiCRISPR v2	Sanjana et al.	RRID: Addgene_52961
pSpCas9(BB)-2A-GFP (PX458)	Ran et al.	RRID: Addgene_48138
pCMVβ-p300-myc	Addgene	RRID: Addgene_30489
pSG5-HA-p300	Addgene	RRID: Addgene_89094
pSG5-HA-p300-DY1399	Addgene	RRID: Addgene_89095
Stat1 alpha Flag pRc/CMV	Addgene	RRID: Addgene_8691
Stat1 beta Flag pRc/CMV	Addgene	RRID: Addgene_8704
**Software and Algorithms**		
Image J 1.49v	NIH	Ran et al.<br>RRID: SCR_003073

FlowJo	FlowJo	https://www.flowjo.com/<br>RRID:SCR_008520
GraphPad Prism	GraphPad	https://www.graphpad.com<br>RRID: SCR_002798
NDP.scan	Hamamatsu Photonics	https://www.hamamatsu.com
NDP.view2	Hamamatsu Photonics	https://www.hamamatsu.com<br>U12388-01
Integrated Genomics Viewer		http://software.broadinstitute.org/software/igv/<br>RRID:SCR_011793
Plotly	Plotly	https://plot.lyd<br>RRID:SCR_013991
Morpheus	Broad Institute	https://software.broadinstitute.org/morpheus
AxioVision Release	Zeiss	https://www.zeiss.com<br>RRID: SCR_002677
Beckman Flow Cytometer Software	Beckman Coulter Life Sciences	https://www.beckman.com/coulter-flow-cytometers/software<br>RRID:SCR_008940
DMi8 Leica Microscope	Leica	https://www.leica-microsystems.com/products/light-microscopes/inverted-microscopes/details/product/leica-dmi8/
Zeiss LSM 880 Confocal with FAST Airyscan	Zeiss	https://www.zeiss.com/microscopy/us/products/confocal-microscopes/lsm-880-with-airyscan-.html
STAR (v2.5.2b)	Dobin et al.	http://code.google.com/p/rna-star/
HOMER		http://biowhat.ucsd.edu/homer/<br>RRID:SCR_010881
DESeq2	Love et al.	http://www.bioconductor.org/packages/release/bioc/html/DESeq2.html<br>RRID:SCR_015687
Cluster 3.0		http://bonsai.hgc.jp/~mdehoon/software/cluster/software.htm<br>RRID: SCR_013505
Java TreeView		http://jtreeview.sourceforge.net/<br>RRID:SCR_016916
cutadapt (1.16)		https://cutadapt.readthedocs.io<br>RRID:SCR_011841
bowtie2 (2.2.8)		http://bowtie-bio.sourceforge.net/bowtie2/index.shtml<br>RRID:SCR_005476
samtools (1.7)		http://samtools.sourceforge.net/<br>RRID:SCR_002105
picard (2.8.12)		https://broadinstitute.github.io/picard/<br>RRID:SCR_006525
awk (3.1.7)	GNU	https://www.gnu.org/software/gawk/
Mouse reference genome mm10		https://www.genome.ucsc.edu/<br>RRID:SCR_005780
Bioconductor 3.6		https://bioconductor.org/<br>RRID: SCR_006442
R Project for Statistical Computing 3.4.3		https://www.R-project.org/<br>RRID:SCR_001905
Adobe Illiustrator CC 2019	Adobe	Version 23.0.2 x64
Cell Ranger v3.1.0		https://support.10xgenomics.com/single-cell-gene-expression/software/pipelines/latest/installation<br>RRID: SCR_017344

Seurat v3.1.5	Butler et al	https://satijalab.org/seurat/
SingleR v.1.0.6	Aran et al.	https://bioconductor.org/packages/release/bioc/html/SingleR.html
Scrublet	Wolock et al.	https://github.com/AllonKleinLab/scrublet
LISA	Qin et al.	http://lisa.cistrome.org/
GIGGLE	Layer et al.	https://github.com/ryanlayer/giggle
MACS v2.1.0	Zhang et al.	https://github.com/macs3-project/MACS/wiki/Install-macs2
ENCODE ATAC-seq pipeline v1.7.0	ENCODE Project Consortium	https://github.com/ENCODE-DCC/atac-seq-pipeline
Serial EM software v3.1.1a		https://bio3d.colorado.edu/SerialEM/<br>RRID:SCR_017293
Adobe Photoshop CS5 Extended version 12.0X64	Abode	https://helpx.adobe.com/photoshop/kb/security-update-photoshop.html<br>RRID:SCR_014199
MaxQuant v1.5.3.17		https://www.maxquant.orgRRID:SCR_014485
Python 2.7		https://www.python.org/download/releases/2.7/<br>RRID: SCR_008394
Seaborn	Python library	https://seaborn.pydata.org/<br>RRID: SCR_018132
Pandas	Python library	http://pandas.pydata.org/<br>RRID: SCR_002511
Biopython	Python library	http://biopython.org/wiki/Seq<br>RRID: SCR_007173
NumPy	Python library	https://numpy.org/<br>RRID: SCR_008633
Aspera connect v3.8.3	IBM	https://downloads.asperasoft.com/connect2/
SRA Toolkit v2.9.2	NCBI	https://github.com/ncbi/sra-tools
**Other**		
ENCODE blacklist file for mm10	(21)	https://sites.google.com/site/anshulkundaje/projects/blacklists
Gene Ontology		http://geneontology.org/<br>RRID:SCR_002811
Mouse Cell Depletion Kit	Miltenyi Biotec	Cat#: 130-104-694
Matrigel	Corning/Fisher	Cat#: CB40234B
Cistrome Database	Zheng et al.	http://cistrome.org/db/
0.22μm Millex 33mm PES sterile filter	Sigma-Aldrich	Cat#: SLGSR33RS

REFERENCES

1. S. Shalapour, *et al.*, Immunosuppressive plasma cells impede T-cell-dependent immunogenic chemotherapy. *Nature* **521**, 94–98 (2015).
2. J. Humeau, S. Lévesque, G. Kroemer, J. G. Pol, "Gold Standard Assessment of Immunogenic Cell Death in Oncological Mouse Models" in *Cancer Immunosurveillance*, Methods in Molecular Biology., A. López-Soto, A. R. Folgueras, Eds. (Springer New York, 2019), pp. 297–315.
3. S. Shalapour, *et al.*, Inflammation-induced IgA^+ cells dismantle anti-liver cancer immunity. *Nature* **551**, 340–345 (2017).
4. K. Meeth, J. Wang, G. Micevic, W. Damsky, M. W. Bosenberg, The YUMM lines: a series of congenic mouse melanoma cell lines with defined genetic alterations. *Pigment Cell Melanoma Res.* **29**, 590–597 (2016).
5. K. A. Hogquist, *et al.*, T cell receptor antagonist peptides induce positive selection. *Cell* **76**, 17–27 (1994).
6. P. Cascio, C. Hilton, A. F. Kisselev, K. L. Rock, A. L. Goldberg, 26S proteasomes and immunoproteasomes produce mainly N-extended versions of an antigenic peptide. *EMBO J.* **20**, 2357–2366 (2001).
7. T. Muchamuel, *et al.*, A selective inhibitor of the immunoproteasome subunit LMP7 blocks cytokine production and attenuates progression of experimental arthritis. *Nat. Med.* **15**, 781–787 (2009).
8. S. K. Chakrabarti, J. C. James, R. G. Mirmira, Quantitative assessment of gene targeting in vitro and in vivo by the pancreatic transcription factor, Pdx1. Importance of chromatin structure in directing promoter binding. *J. Biol. Chem.* **277**, 13286–13293 (2002).
9. D. Dhar, *et al.*, Liver Cancer Initiation Requires p53 Inhibition by CD44-Enhanced Growth Factor Signaling. *Cancer Cell* **33**, 1061-1077.e6 (2018).
10. N. E. Sanjana, O. Shalem, F. Zhang, Improved vectors and genome-wide libraries for CRISPR screening. *Nat. Methods* **11**, 783–784 (2014).
11. F. A. Ran, *et al.*, Genome engineering using the CRISPR-Cas9 system. *Nat. Protoc.* **8**, 2281–2308 (2013).
12. Z. Zhong, *et al.*, New mitochondrial DNA synthesis enables NLRP3 inflammasome activation. *Nature* **560**, 198–203 (2018).
13. E. Godebu, *et al.*, PCSD1, a new patient-derived model of bone metastatic prostate cancer, is castrate-resistant in the bone-niche. *J. Transl. Med.* **12** (2014).
14. J. Drost, *et al.*, Organoid culture systems for prostate epithelial and cancer tissue. *Nat. Protoc.* **11**, 347–358 (2016).
15. A. Butler, P. Hoffman, P. Smibert, E. Papalexi, R. Satija, Integrating single-cell transcriptomic data across different conditions, technologies, and species. *Nat. Biotechnol.* **36**, 411–420 (2018).
16. S. L. Wolock, R. Lopez, A. M. Klein, Scrublet: Computational Identification of Cell Doublets in Single-Cell Transcriptomic Data. *Cell Syst.* **8**, 281-291.e9 (2019).
17. C. Hafemeister, R. Satija, Normalization and variance stabilization of single-cell RNA-seq data using regularized negative binomial regression. *Genome Biol.* **20**, 296 (2019).
18. D. Aran, *et al.*, Reference-based analysis of lung single-cell sequencing reveals a transitional profibrotic macrophage. *Nat. Immunol.* **20**, 163–172 (2019).
19. S. Andrews, *et al.*, FastQC: a quality control tool for high throughput sequence data (2010).
20. A. Dobin, *et al.*, STAR: ultrafast universal RNA-seq aligner. *Bioinformatics* **29**, 15–21 (2013).
21. ENCODE Project Consortium, An integrated encyclopedia of DNA elements in the human genome. *Nature* **489**, 57–74 (2012).
22. M. I. Love, W. Huber, S. Anders, Moderated estimation of fold change and dispersion for RNA-seq data with DESeq2. *Genome Biol.* **15**, 550 (2014).

23. G. Yu, L.-G. Wang, Y. Han, Q.-Y. He, clusterProfiler: an R package for comparing biological themes among gene clusters. *Omics J. Integr. Biol.* **16**, 284–287 (2012).
24. S. Hänzelmann, R. Castelo, J. Guinney, GSVA: gene set variation analysis for microarray and RNA-seq data. *BMC Bioinformatics* **14**, 7 (2013).
25. A. Liberzon, *et al.*, Molecular signatures database (MSigDB) 3.0. *Bioinforma. Oxf. Engl.* **27**, 1739–1740 (2011).
26. Q. Qin, *et al.*, Lisa: inferring transcriptional regulators through integrative modeling of public chromatin accessibility and ChIP-seq data. *Genome Biol.* **21**, 32 (2020).
27. B. Langmead, S. L. Salzberg, Fast gapped-read alignment with Bowtie 2. *Nat. Methods* **9**, 357–359 (2012).
28. Y. Zhang, *et al.*, Model-based analysis of ChIP-Seq (MACS). *Genome Biol.* **9**, R137 (2008).
29. H. M. Amemiya, A. Kundaje, A. P. Boyle, The ENCODE Blacklist: Identification of Problematic Regions of the Genome. *Sci. Rep.* **9**, 9354 (2019).
30. S. Anders, P. T. Pyl, W. Huber, HTSeq--a Python framework to work with high-throughput sequencing data. *Bioinforma. Oxf. Engl.* **31**, 166–169 (2015).
31. R. M. Layer, *et al.*, GIGGLE: a search engine for large-scale integrated genome analysis. *Nat. Methods* **15**, 123–126 (2018).
32. R. Zheng, *et al.*, Cistrome Data Browser: expanded datasets and new tools for gene regulatory analysis. *Nucleic Acids Res.* **47**, D729–D735 (2019).
33. The Cancer Genome Atlas Research Network, *et al.*, The Cancer Genome Atlas Pan-Cancer analysis project. *Nat. Genet.* **45**, 1113–1120 (2013).
34. J. Liu, *et al.*, An Integrated TCGA Pan-Cancer Clinical Data Resource to Drive High-Quality Survival Outcome Analytics. *Cell* **173**, 400-416.e11 (2018).
35. S. A. Shukla, *et al.*, Comprehensive analysis of cancer-associated somatic mutations in class I HLA genes. *Nat. Biotechnol.* **33**, 1152–1158 (2015).
36. I. Hoof, *et al.*, NetMHCpan, a method for MHC class I binding prediction beyond humans. *Immunogenetics* **61**, 1–13 (2009).
37. V. Jurtz, *et al.*, NetMHCpan-4.0: Improved Peptide-MHC Class I Interaction Predictions Integrating Eluted Ligand and Peptide Binding Affinity Data. *J. Immunol. Baltim. Md 1950* **199**, 3360–3368 (2017).
38. R. Marty, *et al.*, MHC-I Genotype Restricts the Oncogenic Mutational Landscape. *Cell* **171**, 1272-1283.e15 (2017).
39. D. E. Larson, T. E. Abbott, R. K. Wilson, Using SomaticSniper to Detect Somatic Single Nucleotide Variants. *Curr. Protoc. Bioinforma.* **45**, 15.5.1–8 (2014).
40. M. Yadav, *et al.*, Predicting immunogenic tumour mutations by combining mass spectrometry and exome sequencing. *Nature* **515**, 572–576 (2014).
